# Hypertensive Disorders of Pregnancy and Cardiovascular Disease Risk Across Races and Ethnicities: A Review

**DOI:** 10.3389/fcvm.2022.933822

**Published:** 2022-06-28

**Authors:** Renée J. Burger, Hannelore Delagrange, Irene G. M. van Valkengoed, Christianne J. M. de Groot, Bert-Jan H. van den Born, Sanne J. Gordijn, Wessel Ganzevoort

**Affiliations:** ^1^Department of Obstetrics and Gynaecology, Amsterdam UMC Location University of Amsterdam, Amsterdam, Netherlands; ^2^Amsterdam Reproduction and Development, Pregnancy and Birth, Amsterdam, Netherlands; ^3^Department of Obstetrics, University Medical Center Groningen, University of Groningen, Groningen, Netherlands; ^4^Department of Public and Occupational Health, Amsterdam UMC Location University of Amsterdam, Amsterdam, Netherlands; ^5^Amsterdam Public Health, Health Behaviors & Chronic Diseases, Amsterdam, Netherlands; ^6^Department of Obstetrics and Gynaecology, Amsterdam UMC Location Vrije Universiteit Amsterdam, Amsterdam, Netherlands; ^7^Department of Vascular Medicine, Amsterdam UMC Location University of Amsterdam, Amsterdam, Netherlands; ^8^Amsterdam Cardiovascular Sciences, Atherosclerosis and Ischemic Syndromes, Amsterdam, Netherlands

**Keywords:** hypertensive disorders of pregnancy, preeclampsia, cardiovascular disease, hypertension, diabetes, ethnicity, chronic kidney disease, dyslipidemia

## Abstract

Pregnancy is often considered to be a “cardiometabolic stress-test” and pregnancy complications including hypertensive disorders of pregnancy can be the first indicator of increased risk of future cardiovascular disease. Over the last two decades, more evidence on the association between hypertensive disorders of pregnancy and cardiovascular disease has become available. However, despite the importance of addressing existing racial and ethnic differences in the incidence of cardiovascular disease, most research on the role of hypertensive disorders of pregnancy is conducted in white majority populations. The fragmented knowledge prohibits evidence-based targeted prevention and intervention strategies in multi-ethnic populations and maintains the gap in health outcomes. In this review, we present an overview of the evidence on racial and ethnic differences in the occurrence of hypertensive disorders of pregnancy, as well as evidence on the association of hypertensive disorders of pregnancy with cardiovascular risk factors and cardiovascular disease across different non-White populations, aiming to advance equity in medicine.

## Introduction

Cardiovascular diseases (CVD) are the number one cause of death globally, with 17.9 million deaths in 2016, representing 31% of all global deaths (1). There are significant differences between women and men in terms of prevalence, presentation, treatment, effects, and prognosis of CVD (2). CVD are diagnosed less often and treated less aggressively in women than in men, likely in part due to practitioners missing the knowledge on the specific risks of women (3, 4). An important non-traditional risk factor for CVD, unique to women, is a history of pregnancy complications, especially hypertensive disorders of pregnancy (HDP) (5).

HDP complicate up to 6–8% of all pregnancies and are a leading cause of maternal and perinatal mortality and morbidity worldwide (6). Registration studies and systematic reviews have consistently shown that women with a history of HDP are at increased risk of subsequent CVD (7–15). HDP are often part of a placental syndrome that is associated with endothelial dysfunction, insulin resistance, oxidative stress, inflammatory activation, and dyslipidemia, all of which may remain in the postpartum period and contribute to an increase in CVD risk (16). Alternatively, it is hypothesized that HDP and future CVD risk are caused by common underlying factors and pregnancy can be seen as a cardiometabolic stress test, potentially identifying those at high CVD risk later in life (16). At highest risk of future CVD are those after the early onset of HDP, with severe and/or recurrent disease (16). The increased CVD risk may be present immediately after pregnancy and persist for more than 20 years (17).

There is substantial heterogeneity in the burden of HDP and CVD across different racial and ethnic (sub) populations, with some disproportionally affected compared to others (18–20). Yet, by far most of the research on the association between HDP and CVD has been conducted in white majority populations (18). A recent review identified that similar to the male-female disparity in research, a disparity exists in the attention to ethnicity: White women are heavily overrepresented in current studies, while there is limited and heterogeneous reporting of race and ethnicity information. Additionally, the potential interaction between race and ethnicity and other sociodemographic variables is not investigated in most studies (18). The few studies that were conducted in multi-ethnic populations and investigated how race and ethnicity interact with HDP on the CVD risk after pregnancy showed contradictory results (21–24).

The lack of research on and understanding of the role of race and ethnicity in HDP-related CVD risk prohibits evidence-based targeted prevention, monitoring, and intervention strategies in multi-ethnic populations and maintains the gap in health outcomes. The aim of this review is to present an overview of the evidence on racial and ethnic differences in the occurrence of HDP, as well as evidence on the association between HDP, cardiovascular risk factors, and CVD later in life in different racial and ethnic (sub) population, aiming to advance equity in medicine.

## Race and Ethnicity: Definitions and Limitations

Important sensitivities and controversies related to use of the terms race, ethnicity and associated nomenclature exist in medical and health research, clinical practice, and society. We agree with Flanagin et al. that “terminology, usage, and word choice are critically important, especially when describing people and when discussing race and ethnicity” (25). In this review, we follow the JAMA guidance for Reporting Race and Ethnicity in Research Articles (26). We chose to use the aggregated “race and ethnicity,” acknowledging that there are numerous subcategories within race and ethnicity (26). When addressing race and ethnicity, we refer to it as a social construct, that is applied to compare different groups based on a given socio-cultural or physical characteristic. When describing and comparing the results of included original studies, we use racial and ethnic categories as they have been applied in the original articles.

## Search Strategy, Selection Criteria, and Data Extraction

**FIGURE 1:**
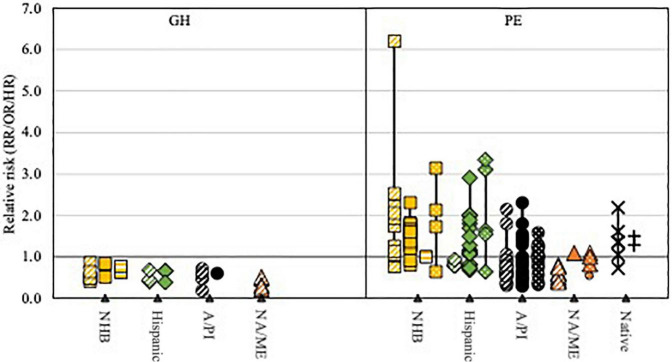
Visual representation of point estimates for relative GH and PE risk reported in the included studies among different racial and ethnic groups compared to non-Hispanic White women. 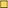 Non-Hispanic Black, African American or Black women; 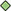 Latina or Hispanic women; 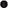 Asian or Pacific Islander women; 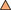 North African or Middle Eastern women (NA/ME); × American Indian/Alaska Native women; + Aboriginal/Torres Strait Islander or Maori women; 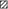 living in Europe; 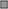 living in the US; 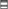 living in South Africa; 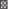 living in another predominantly White country. Study quality and precision of the estimates were not accounted for in the figure, and it should thus be interpreted as an overview of the available evidence, not as a formal statistical summary.

**FIGURE 2:**
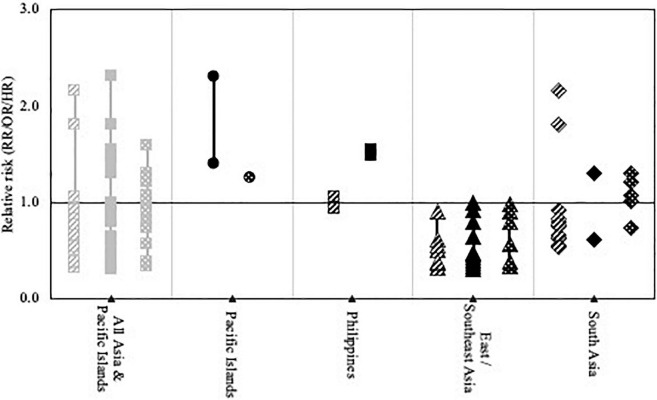
Visual representation of point estimates for relative PE risk reported in the included studies among women of Asian and Pacific Islander origin compared to non-Hispanic White women. 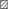 Asian or Pacific Islander women living in Europe; 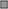 living in the US; 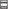 living in South Africa; 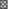 living in another predominantly White country. Study quality and precision of the estimates were not accounted for in the figure, and it should thus be interpreted as an overview of the available evidence, not as a formal statistical summary.

**FIGURE 3:**
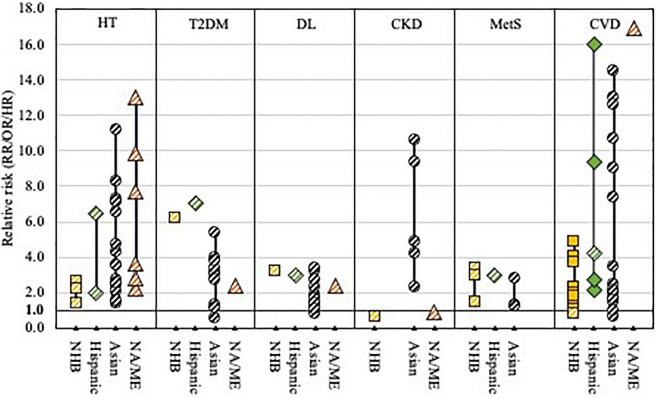
Visual representation of point estimates for CVD risk and CVD risk factors reported in the included studies across races and ethnicities. 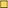 Non-Hispanic Black, African American or Black women; 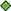 Latina or Hispanic women; 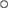 Asian women; 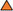 North African or Middle Eastern women (NA/ME); 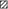 living in country of origin; 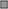 living in the US. Note: study quality and precision of the estimates were not accounted for in the figure, and it should thus be interpreted as an overview of the available evidence, not as a formal statistical summary. HT, hypertension; T2DM, type 2 diabetes mellitus; DL, dyslipidemia; CKD, chronic kidney disease; MetS, metabolic syndrome; CVD, cardiovascular disease.

An extensive systematic literature review was conducted to identify all relevant studies reporting on HDP and CVD risk following HDP in non-White subgroups and populations. We systematically searched PubMed and Embase from inception to February 2022. The full search strategy is available in Supplementary Tables 1, 2. Reference tracing was performed to identify additional studies of interest. Titles and abstracts of all identified studies were screened, after which potentially useful records were reviewed in full. Studies were included if they met the following inclusion criteria: (i) original research, (ii-a) reporting on the incidence, prevalence, or risk of HDP, HDP severity, or HDP-related complications, or (ii-b) reporting on the incidence, prevalence, or risk of CVD and CVD risk factors at least 6 weeks after a pregnancy complicated by HDP, (iii-a) in at least two different racial or ethnic groups, or (iii-b) in non-White (sub) populations. Data on study characteristics and outcomes were extracted from the included studies. Supplementary Tables 3, 4 provide an overview of all included studies and relevant characteristics. Figures 1–3 provide visual representations of point estimates for relative risk (RR, OR, or HR) of different HDP, CVD risk factors and CVD reported in the included studies among different racial and ethnic groups. Studies that did not report a measure of relative risk are not included in the figures. Study quality and precision of the estimates were not accounted for in the figure, and it should thus be interpreted as an overview of the available evidence, not as a formal statistical summary.

## Definitions of Hypertensive Diseases in Pregnancy

Over the years and across countries, many different definitions and criteria have been used for HDP, although the cutoff for blood pressure to classify hypertension has been consistent. For this review, we followed the ISSHP 2018 classification (27). Where possible, we converted terminology used by original authors to fit the ISSHP classification. Hypertension is defined as a systolic blood pressure (BP) ≥140 mmHg and/or diastolic BP ≥ 90 mmHg. Chronic hypertension refers to high BP predating the index pregnancy or recognized before 20 weeks of gestation. Transient gestational hypertension is *de novo* hypertension that develops at any gestation that resolves without treatment during pregnancy. Gestational hypertension (GH) is persistent *de novo* hypertension that develops at or after 20 weeks’ gestation in the absence of features of preeclampsia. Preeclampsia (PE) is GH accompanied by ≥1 of the following new-onset conditions at or after 20 weeks’ gestation: (i) abnormal proteinuria (urine protein/creatinine ratio ≥30 mg/mmol), (ii) maternal organ dysfunction, or (iii) uteroplacental dysfunction (e.g., fetal growth restriction). PE superimposed on chronic hypertension (superimposed PE) is diagnosed if a woman with chronic hypertension develops *de novo* proteinuria or organ dysfunction consistent with preeclampsia. Eclampsia and HELLP syndrome are considered part of the preeclampsia spectrum and not a separate disorder. In this review, we use HDP as an umbrella term for all the above-defined disorders.

## Hypertensive Disorders of Pregnancy Across Racial and Ethnic Groups

We identified *n* = 53 studies that reported on the prevalence of HDP, or on HDP severity and HDP-related complications across different racial and ethnic groups (Supplementary Figure 1 and Supplementary Table 3) (28–80). Different classifications were used to describe race and ethnicity. To demonstrate this, for black women in America, classifications used the term non-Hispanic Black, African American, and Black. Race and ethnicity were determined in 28% by self-report, in 4% by researcher, in 39% based on database/electronic health reports, and in 30% based on maternal country of birth. Definitions of different types of HDP were not always provided and varied across studies (Supplementary Table 3).

### Hypertensive Disorders of Pregnancy

Six studies reported on the overall prevalence of HDP (Supplementary Table 5) (40, 45, 54, 69, 72, 76). HDP prevalence among non-Hispanic Black, African American, and Black women in the United States of America (US) varied from 3.3 to 15.8% (40, 45, 54, 72). Two studies reported significantly increased HDP prevalence among non-Hispanic Black women compared to non-Hispanic White women (OR 1.3, OR other study NR), while two other studies reported no significant difference compared to White women and women of other race or ethnicity. The prevalence of HDP was higher among US-born non-Hispanic Black women (10.3%) than among foreign-born non-Hispanic Black women (7.1%) (40). Among women with Sub-Saharan African origin in Finland, HDP prevalence was 4.2%, which was significantly lower than among Finish women (4.6%; adjusted RR 0.84) (76).

Among Hispanic women in the US HDP prevalence was 4.5–9.1%, which was lower than among US non-Hispanic White women in both studies (40, 54). The prevalence of HDP was higher among US-born Hispanic women (5.3–6.2%) than among foreign-born Hispanic women (4.4–5.9%), except in Puerto Rican women (40). Similarly, HDP prevalence among Latin American women in Finland (2.2%) was significantly lower than among Finnish women (4.6%; adjusted RR 0.52) (76).

Among Chinese, Japanese, Korean, Vietnamese, and Asian-Indian women in the US and/or Australia, significantly lower HDP prevalence was found compared to non-Hispanic White US or Australian-born women (40, 69). Prevalence was higher in those born in the US compared to foreign-born women living in the US (40). Similarly, women from South and East Asian in Finland had significantly lower HDP prevalence (adjusted RR 0.33–0.63) (76). Among Filipino, Samoan and American Indian/Alaska Native (AI/AN) women in the US HDP prevalence was significantly higher than among non-Hispanic White US women (40, 69).

Prevalence of HDP was significantly lower among women from the Middle East and North African in Finland compared to Finish women (76).

### Chronic Hypertension Before or in Pregnancy

Sixteen studies reported on the prevalence of chronic hypertension (Supplementary Table 5) (36, 38, 40, 42, 43, 45, 47–49, 51, 61, 67, 71, 72, 75, 78). Eleven of these studies reported on the prevalence of chronic hypertension among non-Hispanic Black, Black, or African American women in the US (36, 40, 42, 43, 45, 49, 67, 71, 72, 75, 78). In most studies, the prevalence of chronic hypertension in these women ranged between 0.8 and 3.3% and was 1.4–2.3-fold higher than among non-Hispanic White women (36, 40, 43, 45, 49, 67, 71, 72, 78). In one high-risk cohort that was oversampled with women who delivered preterm, a higher chronic hypertension prevalence was found among non-Hispanic Black women (7.8%). However, chronic hypertension prevalence among non-Hispanic White women was similarly increased, resulting in a relative risk for non-Hispanic Black women that was comparable to the other studies (75). The prevalence of chronic hypertension was higher among US-born non-Hispanic Black women than among foreign-born non-Hispanic Black women (43, 75). Among African Caribbean women living in the United Kingdom the incidence of chronic hypertension was 3.3%, significantly higher than among Caucasian women in the United Kingdom (adjusted OR 3.1) (48). In South Africa, the prevalence of chronic hypertension was higher in Black (1.1%) and Colored (1.7%) women compared to White women (0.6%) (61).

Six studies reported on the prevalence of chronic hypertension among Latina or Hispanic women in the US (36, 40, 43, 67, 71, 75). The chronic hypertension prevalence ranged from 0.7 to 1.6%; in a high-risk cohort, oversampled with women who delivered preterm, CH prevalence was 2.5–2.8% (36, 40, 43, 67, 71, 75). The prevalence of chronic hypertension in most Latina/Hispanic groups was similar to, or lower than in non-Hispanic Black and non-Hispanic White women. One study reported lower rates of chronic hypertension among foreign-born compared to US-born Latina or Hispanic women in the US, except in Puerto Rican women; a second study reported no significant difference (43, 75).

Five studies reported the prevalence of chronic hypertension among women from Asian and Pacific Islander (A/PI) origin living in the US (40, 43, 47, 67, 71). Chronic hypertension prevalence ranged from 0.1 to 2.3%. Rates were generally lower than among non-Hispanic White women, except among Filipino and Samoan women, who had increased rates of chronic hypertension. Lowest rates were described among Chinese, Korean, and Asian Indian women. Rates were lower among A/PI women who were born outside of the US compared to US-born A/PI women (43). Lower chronic hypertension rates were reported among Vietnam-born women living in Australia (38). One study found increased rates of chronic hypertension among South Asian women in the United Kingdom compared to Caucasian women (OR 1.9) (48). Rates among A/PI, Native Hawaiian, and White women living in Hawaii were low and similar across groups (0.1–0.3%) (51).

Among AI/AN women in the US, chronic hypertension prevalence was 1.4–1.5-fold increased in two studies compared to non-Hispanic White women (40, 71).

### Gestational Hypertension and/or Preeclampsia

Eleven studies reported on the combined prevalence of GH and PE (Supplementary Table 5) (31, 32, 40, 43, 45, 46, 53, 59, 62, 66, 71). Among US non-Hispanic Black or African American women, GH/PE prevalence ranged from 2.9 to 10.5% (40, 43, 45, 53, 59, 62, 66, 71). In most studies, GH/PE prevalence was slightly higher among these women than among non-Hispanic White women (2.9–9.2%). Both studies that statistically tested the difference found a significantly higher rate of GH/PE among non-Hispanic Black or African American women (adjusted RR/OR 1.3), although in one study no difference was found when the analysis was limited to overweight and obese women (59, 62). Similar to all HDP, the prevalence of GH/PE was higher among US-born non-Hispanic Black women (10.5%) than among foreign-born non-Hispanic Black women (7.1%) (43).

GH/PE prevalence was lower among Hispanic women (1.3–7.8%) than among non-Hispanic White women in the US (31, 40, 43, 53, 59, 66, 71). The difference was statistically significant in two studies, although in one study only in overweight or obese women (59, 66). The rate of GH/PE was lower among foreign-born than among US-born Hispanic or Latina women, expect in Puerto Rican women, where high rates of GH/PE were found in both foreign and US-born women (43).

The three studies comparing GH/PE prevalence between A/PI women and non-Hispanic White women in the US showed lower rates among A/PI women (2.4–6.0%), except in Filipino (5.9–8.1%) and Samoan women (6.8%) (40, 43, 71). Foreign-born A/PI women had lower GH/PE rates than US-born A/PI women (43). Two other studies compared rates of GH/PE among different Asian subgroups (32, 46). They reported the lowest GH/PE rates among East Asian and Southeast Asian women (1.1–1.8%), and higher rates among Filipino (2.9–6.3%), South Asian (1.8–3.3%), and Pacific Island women (2.3–4.8%) (32, 46).

One study reported higher rates of GH/PE among non-Hispanic American Indian women, compared to non-Hispanic White women in the US (5.3% vs. 4.5%) (71).

### Gestational Hypertension

Nine studies reported on the prevalence of GH (Supplementary Table 5) (33, 36, 39, 45, 61, 67, 72, 74, 76). Figure 1 provides a visual overview of the GH risk among different non-White populations compared to non-Hispanic White women reported in these studies. In three US studies, lower rates of GH were reported in non-Hispanic Black or Black women (1.3–3.6%) compared to non-Hispanic White or White women (2.0–4.5%) (36, 67, 72). Among women from Sub-Saharan origin in Finland and Norway, GH rate was significantly lower than among Finish or Norwegian women (ORs 0.5) (39, 76). Women from Surinamese-Creoles (3.2%), Cape Verdean (3.2%), and Antillean origin (2.9%) in the Netherlands had lower rates of GH than Dutch women (5.2%), but the difference was not statistically significant (74). In South Africa, the overall prevalence of GH was high, with highest rates among White women (14.1%) and lowest rates among Black women (8.8%) (61).

The prevalence of GH varied from 1.2 to 2.4% among Hispanics in the US, significantly lower than among non-Hispanic White or Caucasian women in two studies (adjusted OR 0.6, adjusted RR 0.4) (33, 36, 67). GH prevalence was also significantly lower among Latin American/Caribbean women in Finland and Norway compared to Finish and Norwegian women (adjusted RR 0.4, adjusted OR 0.5–0.7) (39, 76).

The prevalence of GH among A/PI women was 1.7% (one study), significantly lower than among non-Hispanic White women (adjusted OR 0.6) (20, 67). Prevalence of GH was also significantly lower among in South Asian (1.1–1.6%, adjusted OR 0.6–0.7) and East Asians (0.3–1.2%, adjusted OR 0.5–0.6) compared to Finish (2.3%) and Norwegian women (1.5–2.4%) (39, 76). Women of Oceanian origin in Norway had similar GH rates as Norwegian women (39). Among Surinamese-Hindustani women in the Netherlands, GH prevalence was 3.4%, compared to 5.2% in Dutch women (not significant) (74).

Women from Middle Eastern or North African origin in Finland (0.6%) and Norway (0.8–1.1%) had significantly lower GH rates compared to Finish (adjusted RR 0.2) and Norwegian women (adjusted OR 0.5) (39, 76). Turkish (1.7%) and Moroccan (1.5%) women in the Netherlands had significantly lower GH rates than Dutch women (adjusted ORs 0.3) (74).

### Preeclampsia

Thirty-four studies reported on the prevalence of PE (Supplementary Table 5) (29, 30, 33–39, 41, 42, 44, 45, 47, 49–52, 55, 56, 58, 60, 61, 63, 65, 67, 70, 72–77, 80). Figure 1 provides a visual overview of the PE risk among different non-White populations compared to non-Hispanic White women reported in these studies. The prevalence of PE was significantly higher among non-Hispanic Black, Black, and African American women compared to non-Hispanic White or White women in the US in most studies (adjusted OR 1.2–2.3), ranging from 2.5 to 8.3% (29, 36, 42, 45, 49, 55, 56, 60, 63, 65, 67, 72, 73, 75). In one high-risk cohort, oversampled with women who delivered preterm, a higher PE prevalence was found among non-Hispanic Black women (9.2–12.2%), but PE prevalence among non-Hispanic White women was similarly increased, so that the relative risk for non-Hispanic Black women was comparable to the other studies (75). One study reported higher PE prevalence among US-born non-Hispanic Black women than among foreign-born non-Hispanic Black women, although after 10 years of residence in the US, the difference was no longer statistically significant (75). Another study found no difference between US-born and foreign-born non-Hispanic Black women (65). Among women from Sub-Saharan Africa in Canada, risk of severe PE was significantly increased (0.7%, adjusted OR 3.1) compared to women from industrialized countries (34). In Israel, Ethiopian women had significantly higher rates of mild and severe PE compared to Israeli women (44). Prevalence of PE was significantly increased among women of Sub-Saharan African origin in Finland (3.0%, adjusted RR 1.8), in France (severe PE 1.6%, adjusted OR 2.5) and Australia, Canada, Spain, US, Denmark, and Sweden (2.8%, adjusted OR 1.7) compared to White native populations (35, 41, 76). Three Norwegian studies showed no significant difference in PE risk among women of Sub-Saharan African origin compared to Norwegian women, except in women from Burundi (5.9%, adjusted OR 1.8), Congo (5.9%, adjusted OR 1.9), Tanzania (7.4%, adjusted OR 2.2) and Somalia (4.0%, adjusted OR 1.3) (39, 50, 52). Among Cape Verdean women in the Netherlands, PE rate was significantly increased (4.2%, adjusted OR 2.1), while no significant difference was seen between Surinamese Creole (2.4%) and Dutch women (1.9%) (74). Among Sub-Saharan African women in Australia, prevalence of PE was significantly lower than among Australian or New Zealand born women (3.5% vs. 4.8%, adjusted OR 0.6) (80). In South Africa, no difference was seen in PE prevalence among White, Black or Colored women (2.9%) (61).

The prevalence of PE among Hispanic and Haitian women in the US ranged from 2.6 to 5.9%; in one high-risk cohort oversampled with women who delivered preterm PE prevalence was 7.9–9.1% (33, 36, 42, 49, 56, 60, 63, 65, 67, 75). In most US studies, PE rates were higher in Hispanic women than in non-Hispanic White women (adjusted OR/HR 1.1–2.9), but lower than in non-Hispanic Black women (33, 36, 56, 60, 63, 65, 67, 75). No difference was reported in PE rate among US-born and foreign-born Hispanic women in the US (65, 75). Severe PE was more prevalent among Hispanic and Caribbean women in Canada (0.6 and 0.7%; adjusted OR 2.0 and 3.3) (35). No significant difference was found in PE rate among Latin American and Caribbean women in Finland and Norway compared to Finish and Norwegian women, except for multiparous women in Norway (adjusted OR 0.8) (39, 50, 76). PE prevalence among Antillean women in the Netherlands was 3.7% compared to 1.9%, but the difference was not statistically significant (74). Prevalence of PE was significantly increased among women of Latin American and Caribbean origin in Australia, Canada, Spain, US, Denmark and Sweden (2.8% vs. 1.8%, adjusted OR 1.6) compared to White native populations (35). Another study reported 3.4% PE among Latin American and Caribbean women in Australia, which was significantly lower than among Australian or New Zealand born women (4.8%; adjusted OR 0.6) (80).

The prevalence of PE among A/PI women in the US and Hawaii ranged from 1.5 to 6.8% (47, 51, 56, 63, 65, 67, 70). Figure 2 provides a visual overview of the PE risk among different A/PI populations compared to non-Hispanic White women reported in these studies. PE risk (1.4–3.7%) was lowest among East Asian women and significantly lower than among non-Hispanic White women in most studies (adjusted OR 0.6–0.9) (47, 51, 65, 67, 70). Among women from South Central Asia, prevalence of PE was increased (2.2%, adjusted OR 1.3) compared to non-Hispanic White women (65). Similarly, Philippine women in the US and Hawaii had significantly higher PE rates (4.0–6.8%; adjusted OR 1.6–2.8) (47, 51, 65). Among other Southeast Asian women in the US prevalence of PE (1.7–2.8%) was not significantly different from non-Hispanic White women (70). Foreign-born Southeast Asian and Pacific Island women had higher PE risks compared to US-born Southeast Asian and Pacific Island women (65). In Canada, the risk of severe PE was significantly increased in one study among women of East Asian and Pacific origin (adjusted OR 1.6), but not in South Asian women (34). Among East Asian and Southeast Asian women in Finland, Norway, New Zealand, Australia, Canada, Spain, US, Denmark, and Sweden, PE prevalence was significantly lower than among the White populations in most studies (adjusted OR/RRs 0.3–0.9) (35, 39, 50, 52, 76, 77, 80). No significant difference was found in PE prevalence among Filipino, Indian, Myanmarese, or Oceanian women in Norway, Indian women in New Zealand, and South Asian women in Finland, Australia, Canada, Spain, US, Denmark, and Sweden, compared with White populations (35, 50, 52, 76, 77). Two other studies found significantly decreased PE rates among South Asian women in Norway and Australia (adjusted OR 0.6-0.8) (39, 80). PE prevalence was 3.8% among Surinamese-Hindustani women in the Netherlands compared to 1.9% among Dutch women, but the difference was not statistically significant (74). In Singapore, women from Malay origin had significantly higher risk of PE (4.2%) and severe PE (0.4%) than Chinese (3.5 and 0.3%) and Indian women (2.6 and 0.2%) (37, 58).

Among women of North African and Middle Eastern origin in Finland, Norway, the Netherlands, Australia, Canada, Spain, US, Denmark, and Sweden the prevalence of PE (0.6–2.7%) was similar to or lower (adjusted OR 0.3–0.6) than the PE prevalence among non-Hispanic White women (34, 35, 39, 41, 50, 52, 65, 74, 76, 80).

Significantly increased risk of PE was found among AI/AN, Native American, and Native Hawaiian women in most studies (4.0–8.9%, adjusted OR 1.1–1.4) (30, 51, 60). Among Maori women in New Zealand PE rate was significantly increased (4.7%, adjusted OR 1.5), while among Aboriginal and Torres Strait Islanders women, no difference in PE risk was found (77, 80).

### Eclampsia

Nine studies reported on eclampsia prevalence separately (Supplementary Table 5) (28, 29, 32, 35, 40, 66–68, 71). Eclampsia occurred in 0.1–0.7% of non-Hispanic Black, Black, or African American women in the US, compared to 0.1–0.3% among non-Hispanic White women (29, 40, 66–68, 71). In two studies the risk of eclampsia was significantly higher in non-Hispanic Black women than in non-Hispanic White women; one study showed no significant difference (66–68). Among Sub-Saharan women living in the Netherlands (RR 6.2) and Australia, Canada, Spain, US, Denmark, or Sweden (0.1%, adjusted OR 2.1), risk of eclampsia was significantly elevated compared to White populations (28, 35).

Eclampsia was observed in 0.1–0.4% of Hispanic women in the US (40, 66–68, 71). Results were mixed: one study reported significantly lower rates of eclampsia among Hispanic women compared to non-Hispanic White women; one study reported higher rates of eclampsia (adjusted OR 1.3); one study reported no significant difference (66–68). A significantly higher risk of eclampsia was also described in women with Surinamese or Antillean origin in the Netherlands (RR 2.5) and in Latin American or Caribbean women (adjusted OR 1.6) in Australia, Canada, Spain, US, Denmark or Sweden (28, 35).

The prevalence of eclampsia among AP/I women in the US ranged from < 0.1 to 0.5% (32, 40, 67, 68, 71). The risk of eclampsia among A/PI women did not differ significantly from non-Hispanic White women (67, 68). Among A/PI women, lowest eclampsia prevalence was seen in East Asian (0.1–0.2%), Southeast Asian (0.1%) and Asian Indian women (0.1%), while Filipino (0.2–0.3%, adjusted OR 3.0) and Pacific Island women (0.3–0.5%, adjusted OR 4.2–6.1) had significantly higher eclampsia risk compared to Chinese women (33, 40). No difference was found in eclampsia prevalence among South Asian and Southeast women in Australia, Canada, Spain, US, Denmark, or Sweden compared to White populations (≤ 0.1%) (35).

Among AI/AN or Native American women, eclampsia prevalence was 0.1–0.6%, not significantly different from non-Hispanic White women in one study (40, 68, 71).

Among Moroccan and Turkish women in the Netherlands, and among North African and Middle Eastern women in Australia, Canada, Spain, US, Denmark, or Sweden, eclampsia prevalence (1.0%) was comparable to the White populations (28, 35).

### Superimposed Preeclampsia

The prevalence of PE superimposed on chronic hypertension was reported in five US studies (Supplementary Table 5) (29, 36, 56, 67, 72). Among non-Hispanic Black, non-Hispanic African American, and Black women, superimposed PE prevalence ranged from 0.4 to 1.0% compared to 0.1–0.3% among non-Hispanic White and White women (29, 36, 56, 67, 72). One study reported a statistically significant difference (OR 2.0) (67). Among Hispanic (0.3–0.4%) and A/PI women (0.2–0.4%) in the US, superimposed PE prevalence was not significantly different from non-Hispanic White women (36, 56, 67). Non-Hispanic Black women with chronic hypertension in the UK were less likely to develop superimposed PE compared to White women with chronic hypertension in one study (13% vs. 17%) (57). Indo-Asian women with chronic hypertension were at a similar risk of developing superimposed PE as White women in the UK (19% vs. 17%) (57).

### Severity and Hypertensive Disorders of Pregnancy-Related Complications

Among hypertensive women, pregnancy outcomes differed by race, with non-Hispanic Black women having the poorest outcome (Supplementary Table 5) (29, 42, 45, 52, 53, 55, 57, 61, 64, 70, 72, 79). Non-Hispanic Black women with PE were significantly more likely to suffer severe maternal morbidity (9.8%, adjusted OR 1.4, definition study-specific) and eclampsia (1.7%) (64). Non-Hispanic Black women with HDP or PE had 3–5-fold increased risk of maternal mortality compared to non-Hispanic White women with HDP or PE (42, 64, 79). Also, African American, Black of African Caribbean women with HDP were at significantly higher risk for intrauterine fetal death (IUFD; adjusted OR 2.5), perinatal mortality (3.8% vs. 1.6%), and neonatal morbidity (adjusted OR 1.1) (42, 55, 57). Preterm birth (PTB), low birthweight (LBW), and delivery of an infant small for gestational age (SGA) were more prevalence among non-Hispanic Black, African American, or African Caribbean women in the US and Europe compared to White women with HDP (29, 52, 53, 57).

The risk of severe maternal morbidity (7.7% vs. 6.1%) or eclampsia (1.6% vs. 1.3%) was slightly higher in Hispanic women with PE than in non-Hispanic White women with PE, but lower than among non-Hispanic Black women (64). No differences were found in HDP-related mortality, perinatal mortality, and PTB risk in Hispanic women with HDP compared to non-Hispanic White women with GH or PE (42, 53). Hispanic women with GH or PE significantly more often had an LBW infant (adjusted OR 1.5) (53).

Severe maternal morbidity occurred in 7.5% of A/PI with PE in the US, significant more often than among non-Hispanic White women (adjusted OR 1.2) (64). One study among Indo-Asian women with chronic hypertension in the UK reported a very high perinatal mortality risk compared to White women (10% vs. 2%) (57). Two studies reported higher PTB rates among Southeast and South Asian women with PE and chronic hypertension (32.45%) compared to White women with PE or chronic hypertension; another study found no significant difference (52, 57, 70). Delivery of an LBW or SGA infant and neonatal admission >72 h was more prevalent among Southeast Asian women with PE or chronic hypertension compared to White women with PE or chronic hypertension (57, 70).

No significant difference in severe maternal morbidity was found between Native American women and non-Hispanic White women with PE (64). Prevalence of PTB among women with PE was increased in women from Afghanistan and Iraq in Norway compared to Norwegian women (52).

## Cardiovascular Disease Risk Following Hypertensive Disorders of Pregnancy Across Racial and Ethnic (Sub) Populations

We identified *n* = 62 studies that reported on the incidence, prevalence, or risk of CVD and CVD risk factors after HDP in non-White subgroups and populations (Supplementary Figure 2 and Supplementary Table 4) (22–24, 81–139). Most of the studies came from the Asian continent (*n* = 30), followed by Sub-Saharan Africa (*n* = 13), North Africa and the Middle East (*n* = 9), and South and Middle America (*n* = 4). One European study and five North American studies reported on Black, Non-Hispanic Black, Hispanic, and African American women. Figure 3 provides a visual overview of the different CVD risk factors and CVD risk among different non-White populations compared to non-Hispanic White women reported in these studies.

### Hypertension

The majority of the studies reported on the risk of hypertension after a pregnancy complicated by HDP (*n* = 42; Supplementary Table 6A) (81–83, 86, 87, 89–94, 97, 98, 101–106, 109–120, 122–124, 128, 132–134, 138, 139). All but one of the comparative studies showed a significantly increased risk of hypertension after pregnancy complicated by HDP, although follow-up time, absolute prevalence/incidence, and risk ratios differed substantially across different studies. Five studies among Chinese, Sudanese, Nigerian, South African, and Ugandan women reported a substantial prevalence of hypertension 6 weeks after pregnancy complicated by PE (28–36%) and GH/PE (26%) (90, 109, 113, 117, 119). Three months to 1 year after pregnancy, the prevalence of hypertension among Indian, Cameroonian, Ugandan, Cuban, and Black Dutch women with PE was 15–38%; among Kenyan women with GH/PE prevalence of hypertension was 24% (91, 101, 102, 112, 114, 120). Prevalence of hypertension was 22% 1 year after pregnancy in Nigerian women with GH, and 61% in women with PE (98).

Studies with longer follow-up times (mean 5–35 years) among Japanese (adjusted OR 2.6–7.1), Korean (RR 2.1, adjusted OR 1.53), Singaporean (adjusted RR 3.6), and Taiwanese women (adjusted HR 8.3–11.2) reported a significantly increased risk of hypertension after pregnancy complicated by GH/PE or HDP compared to women without a history of GH/PE or HDP (86, 97, 104, 106, 110, 118, 128, 133, 139). Risk of hypertension was similarly increased after GH and PE in two Taiwanese studies (97, 139). Among Brazilian women, risk of hypertension was 2–6-fold increased on average 13–15 years after GH/PE or HDP (89, 94). Significantly higher rates of hypertension (mean follow-up time 6–10 years) were also reported among Iranian women with a history of PE (adjusted HR 3.6) or GH/PE (adjusted RR 2.8), Jordanian women with history of PE (RR 13.0) or GH (RR 7.7), Pakistani women with a history of HDP (adjusted OR 2.2) and Turkish women with a history of PE (RR NR) (81, 83, 90, 93, 122–124). Among Tanzanian women, prevalence of hypertension was increased (29% vs. 13%) 5–7 years after pregnancy complicated by PE (111). Among US women, higher rates of hypertension after PE were reported in non-Hispanic Black women (21%) compared to Hispanic and Non-Hispanic White women on average 3 years postpartum (105).

### Type 2 Diabetes Mellitus and Prediabetes Mellitus

Fourteen studies reported on type 2 diabetes mellitus (T2DM), four on T2DM or prediabetes (Supplementary Table 6B) (83, 86, 88, 92–94, 97, 103, 104, 110, 118, 120, 126, 129, 131, 133, 138, 139). Mean length of follow-up varied from 2.6 to 30.7 years. Most studies (*n* = 11) showed significantly increased rates of T2DM or prediabetes after HDP. Five studies from Taiwan reported significantly higher incidence rates of T2DM after GH/PE (adjusted HR 2.7–3.4), after PE (adjusted HR 3.1–5.4), and after GH (adjusted HR 3.3) compared to normotensive pregnancies (97, 103, 129, 131, 139). Studies from India (33%) and Indonesia (16% after early onset PE; 23.5% after late-onset PE) showed high rates of T2DM 5–10 years after PE, but no comparison group was available (88, 92). Studies from Brazil (RR 7.1) and Iran (adjusted RR 2.4) showed significantly higher T2DM rates after GH/PE (93, 94). Among Japanese (4 studies) and Turkish women (1 study), both with and without HDP, the reported rates of T2DM were substantially lower and no significant association between HDP and T2DM was reported (83, 104, 110, 118, 133). Four studies among Korean (adjusted HR 1.1), Thai (RR 4.0), and Kenyan (adjusted RR 6.2) women reported significantly increased rates of prediabetes after PE/GH (86, 120, 126, 138).

### Dyslipidemia

Fourteen studies reported on dyslipidemia (Supplementary Table 6C) (86, 88, 92–94, 103, 104, 110, 118, 120, 129, 133, 138, 139). Overall, the prevalence of dyslipidemia differed largely over the different study population. High rates of dyslipidemia were reported in Indian (33%) and Indonesian women (58% high triglycerides after early-onset PE, 40% after late-onset PE) 5–10 years after PE (88, 92). Even higher rates (87% after GH/PE; 66% after normotensive pregnancy; *p* = 0.01) were reported in a population-based cohort of Irani women 10 years after pregnancy (93). Four studies were conducted among Japanese women with (dyslipidemia 9.9–42.4%) and without a history of HDP (dyslipidemia 2.6–14.2%); two reported significantly higher rates of dyslipidemia after GH/PE (adjusted OR 3.2 and 1.4) (104, 110, 118, 133). Prevalence (1.5% vs. 0.5% and 4.5% vs. 2.8%) and incidence rates (15.0 vs. 4.4 per 1,000 person-years) in Taiwanese women were overall low, but significantly higher after a pregnancy complicated by PE (HR 3.4), HDP (RR 1.6) and GH/PE (adjusted OR 2.29) compared to women without a history of HDP (103, 129, 139). Among Korean women, dyslipidemia was significantly more prevalent on average 10 years after HDP (RR 1.3) than among women without HDP, while no association between PE and dyslipidemia 23 years after pregnancy was noted (86, 138). Among Kenyan women, dyslipidemia was significantly more prevalent among women with a history of GH/PE (adjusted RR 3.25) than among women without a history of GH/PE (120). No significant association was found between HDP and dyslipidemia 10–20 years after pregnancy in Brazilian women (20% vs. 6.7% in women with and without a history of HDP) (94).

### Chronic Kidney Disease

Ten studies reported on the association between HDP and chronic kidney disease (CKD; Supplementary Table 6D) (84, 97, 100, 101, 110, 118, 123, 130, 136, 137). Among Japanese women, no association was found between GH/PE and CKD 5 years after pregnancy, while significantly higher rates of CKD were reported in women with HDP compared to normotensive pregnancies on average 31 years after pregnancy (adjusted OR 4.85) (110, 118). Three of four studies in Taiwanese women reported significantly higher incidence rates of CKD after GH/PE (adjusted HR 4.3), GH (adjusted HR 5.8), PE (adjusted HR 9.5), chronic hypertension (adjusted HR 16.0), and superimposed PE (adjusted HR 44.7), and increased rates of ESRD after GH (adjusted HR 12.4) and PE (adjusted HR14.0), 6–9 years after pregnancy compared to women without a history of HDP (97, 130, 136, 137). In Iranian women, one study found higher rates of proteinuria after PE compared to normotensive pregnancy (20% vs. 0%) on average 6 years after pregnancy; another study did not find an association between PE and CKD (84, 123). In women from Cameroon, proteinuria was reported in 1.8% of women with severe PE 6 months after pregnancy (101). In Nigerian women, 3.5% of women had CKD at 1 year after pregnancy complicated by HDP (100).

### Metabolic Syndrome

The association between HDP and metabolic syndrome was reported in 11 studies, all using slightly different definitions of metabolic syndrome (Supplementary Table 6E) (86, 88, 89, 92, 99, 106, 108, 110, 117, 120, 138). Studies among Korean (adjusted OR 1.2 and 1.3), Brazilian (RR 2.9), and Kenyan women (adjusted RR 3.0) showed significantly increased risks of metabolic syndrome after HDP (86, 89, 120, 138). Among Singaporean and South African women, rates of metabolic syndrome were increased after GH/PE, but the difference did not reach statistical significance (106, 117). In Japanese women, no difference in metabolic syndrome prevalence was reported (110). Increased rates of metabolic syndrome 1 year after HDP compared to normotensive pregnancy were reported among Nigerian women (99).

### Cardiovascular Diseases

CVD risk after a pregnancy complicated by HDP was investigated in 17 studies (Supplementary Tables 6F–H) (22–24, 85, 95–97, 103, 107, 121, 124, 125, 127, 129, 135, 136, 139). Three Taiwanese studies reported significantly increased incidence rates of combined CVD after GH/PE (adjusted HR 2.0), GH (HR 2.0), and PE (HR 3.0 and adjusted HR 6.4) (107, 137, 139). The incidence rate of congestive heart failure (HF) was also significantly increased after PE (HR 7.4) among Taiwanese women (103). Among Brazilian women, prevalence of CVD was increased fourfold (*p* = 0.002) (125). Among non-Hispanic Black women in the US, the incidence rate of HF was significantly increased after GH/PE (adjusted HR 3.74) and superimposed PE (adjusted HR 4.88), but not after chronic hypertension (22). Although overall rates of HF were higher among non-Hispanic Black women with a history of PE/GH or superimposed PE than among non-Hispanic White women with a history of GH/PE (2.28 vs. 0.96 per 1,000 persons-years) or superimposed PE (4.30 vs. 1.22 per 1,000 person-years), the hazard ratios for HF were similar in both groups, and no significant interaction between HDP and race for incident HF was found (22). One study among Cameroonian women showed a significantly decreased risk of CVD after PE, but the authors conclude that this unexpected result was potentially attributable due to selection bias among the control group (127).

The incidence rate of stroke was significantly higher among Korean women with a history of PE (adjusted OR 1.6) and non-Hispanic Black women in the US with a history of GH/PE (adjusted HR 1.7) or superimposed PE (adjusted HR 4.0) (23, 121). Although overall rates of stroke were higher among non-Hispanic Black women in the US with a history of PE/GH than among non-Hispanic White and Hispanic women in the US with a history of GH/PE (0.32 vs. 0.20 vs. 0.15 per 1,000 persons-years), no significant interaction between HDP and race for incident stroke was found (23). In non-Hispanic Black women stroke risk was significantly increased in women with history of superimposed PE (adjusted HR 4.0), while among non-Hispanic White women the difference was not statistically significant (adjusted HR 1.9). However, no significant interaction between superimposed PE and race for incident stroke was found (23). Among Taiwanese women, a significantly increased risk of stroke after pregnancy complicated by HDP (adjusted HR 1.7–2.1), GH/PE (adjusted HR 2.0), GH (adjusted HR 1.7), PE (adjusted OR 1.6–2.1, HR 2.0–3.5), and superimposed PE (adjusted HR 3.1–3.9) were noted (95, 96, 103, 107, 129). Two Taiwanese studies found no significant association of GH and GH/PE with stroke (95, 97).

Ischemic heart disease (IHD) was significantly increased among Taiwanese women with a history of (superimposed) PE (adjusted HR 13.0), Iranian women with a history of PE (adjusted HR 16.9), and non-Hispanic Black and Hispanic women in the US with a history of GH/PE (adjusted HR 2.3 and 2.7, respectively) and superimposed PE (adjusted HR 4.0 and 9.4, respectively) (23, 85, 107). Although overall rates of IHD were higher among non-Hispanic Black women in the US with a history of PE/GH or superimposed PE than among non-Hispanic White and Hispanic women in the US with a history of GH/PE (1.52 vs. 0.88 vs. 0.34 per 1,000 persons-years) or superimposed PE (3.51 vs. 1.18 vs. 1.57 per 1,000 person-years), no significant interaction between GH/PE or superimposed PE and race for IHD was found (23). Additionally, the same study also did not find evidence for interaction between chronic hypertension and race for incident IHD (23). Two other studies among Taiwanese women, and one study among Black women in the US did not find a significant association between GH/PE and IHD (97, 129, 135).

CVD-related mortality was significantly increased among Taiwanese women with a history of PE/GE (adjusted HR 2.0) and (superimposed) PE (adjusted HR 6.4) (107, 137). Among US women a significant interaction between race and GH was found: African American women with a history of GH had an increased CVD mortality risk (adjusted HR 1.8), while among non-African Americans with a history of GH, no significant increase in CVD mortality risk was found (adjusted HR 0.9) (24).

## Discussion

Our review identified evidence on the risk of HDP and on the risk of CVD and CVD risk factors after a pregnancy complicated by HDP in non-White populations. It serves as an overview of the current evidence, and of gaps in the literature that need additional attention.

Compared to non-Hispanic White women in the US, prevalence of chronic hypertension, (superimposed) PE and eclampsia, but not GH, seemed increased among non-Hispanic Black women. Women from Sub-Saharan African origin in Europe mostly had lower rates of HDP, but higher rates of PE in part of the studies than White women. Combined HDP prevalence was lower among Hispanic US women than among non-Hispanic White women, but PE prevalence was increased. Women from East Asian and Southeast Asian origin both in the US and in Europe, and North African or Middle Eastern women in Europe seemed at decreased risk for HDP compared to non-Hispanic White women. In most studies, rates of HDP were lower among those born in their country of origin compared to women of the same origin born in the host country, and risks converged toward that of the host population with increasing duration of residence. While most studies accounted in their analyses for common confounders (i.e., maternal age, parity, socio-economic status, education level, BMI), understanding of sociodemographic, economic, or health behavioral factors underlying these differences is limited. It has to be noted that a fairly large part of the studies we identified used maternal country of birth as proxy for race and ethnicity, potentially misclassifying part of their population.

The results from the included articles on CVD risk after HDP among different racial and ethnic groups presented in this review are generally in line with results of in the previously published, systematic reviews and meta-analyses that have included a predominantly White population. The current review was designed to extend this work with an overview of the evidence on CVD risk after HDP in different racial and ethnic groups. The articles on CVD risk after HDP included in this review are almost exclusively published in the last decade. For that reason, most of these results are not included in the large meta-analyses that were published on this topic and suffer from overrepresentation of White women (8, 12–14, 16). It is important to ensure a racially and ethnically diverse study population in individual studies and systematic reviews, representative of the real-word diversity, to improve generalizability of outcomes and clinical recommendations. Therefore, updates of these systematic reviews and meta-analysis, and subsequently the guidelines based on this evidence, is needed in the future. Moreover, the studies identified in this current topical review are primarily from the Asian continent, and African women are still underrepresented. Better studies, especially on long-term CVD risk, are needed among these women.

We find contradicting evidence on differential CVD risk after HDP across racial and ethnic groups. Only five of the 62 identified studies reported on CVD risk after a pregnancy complicated by HDP in more than one racial or ethnic group (22–24, 105, 114). Three of these studies formally tested for interaction between the exposure variable and race for the studied CVD outcome. Two studies found no evidence of interaction between HDP (GH/PE, chronic hypertension, or superimposed PE) and race for incident HF, IHD, and stroke (22, 23). A third study did find a significant interaction between GH and race, with GH being a significant marker for CVD risk only for African American women (24). Further studies in multi-ethnic populations are needed to study the potential influence of race and ethnicity on the association between HDP and CVD risk in more detail, taking into account other relevant socio-economic parameters.

Another topic of interest, largely outside the scope of is this review, that needs to be taken into account is the role of migration on the risk of HDP and CVD after HDP among different racial and ethnic groups. Women with a migration history form a distinct group because their health is influenced both by the situation and presence of risk factors in the homeland and in the host country. This review showed higher rates of HDP among non-White women born in the host country compared to women born in their country of origin who migrated to the host country. A better understanding of factors underlying these differences and targets for prevention of this increase in HDP risk could improve the overall health of non-White women. This is of particular interest as migration is expected to rise further in most contexts over the next years (39, 52).

It is remarkable that the risk of GH in non-White women is lower or similar compared to that found in non-Hispanic White women, while chronic hypertension, (superimposed) PE, and eclampsia risk in increased among most non-White populations. Further research on mechanisms underlying the racial and ethnic differences, including pre-existing cardiovascular risk profile, access to health care, interventions (e.g., iatrogenic delivery), and other obstetric characteristics, is needed to provide an explanation for this observation, and other differences identified in this review.

In conclusion, this review highlights that there are racial and ethnic differences in the prevalence of all types of HDP but that the body of literature is yet insufficient to draw firm conclusions. HDP is associated consistently with increased CVD risk across racial and ethnic groups, but further studies on potential differences and their etiology are required. Meta-analyses and guidelines should be updated and based on evidence from more racially and ethnically diverse study populations. This may contribute to a better understanding of the pathogenesis of HDP and subsequent CVD risk, improve monitoring strategies and allow timely interventions to reduce the unequal burden of HDP and CVD across races and ethnicities.

## Author Contributions

WG conceptualized the review. RB and HD performed the literature search and data extraction and wrote the manuscript. All authors critically reviewed the manuscript and gave final approval for publication.

## Conflict of Interest

WG reported holding government funding (ZonMW 843002825) and (unrestricted) free of charge test kits from Roche Diagnostics. SG reported holding government funding (ZonMW 852002034) and (unrestricted) free of charge test kits from Roche Diagnostics. The remaining authors declare that the research was conducted in the absence of any commercial or financial relationships that could be construed as a potential conflict of interest.

## Publisher’s Note

All claims expressed in this article are solely those of the authors and do not necessarily represent those of their affiliated organizations, or those of the publisher, the editors and the reviewers. Any product that may be evaluated in this article, or claim that may be made by its manufacturer, is not guaranteed or endorsed by the publisher.
