# Supplementary material for: Hypertensive Disorders of Pregnancy and Cardiovascular Disease Risk Across Races and Ethnicities: A Review
Source: Front Cardiovasc Med. 2022 Jun 28;9:933822. doi: 10.3389/fcvm.2022.933822 (PMC9273843; doi:10.3389/fcvm.2022.933822)
Supplement: Supplementary file 1 [file Data_Sheet_1.pdf]

SUPPLEMENTARY MATERIAL

Hypertensive disorders of pregnancy and cardiovascular disease risk across races and ethnicities, a review

R.J. Burger[1,2†], H. Delagrange[3†] I.G.M. van Valkengoed[4,5], C.J.M. de Groot[2,6], B.J.H. van den Born[7,8], S.J. Gordijn[3], W. Ganzevoort[1,2]

[1] Amsterdam UMC location University of Amsterdam, Department of Obstetrics and Gynaecology, Meibergdreef 9, Amsterdam, The Netherlands.
[2] Amsterdam Reproduction and Development, Pregnancy and Birth, Amsterdam, The Netherlands.
[3] University Medical Center Groningen, University of Groningen, Department of Obstetrics, Groningen, the Netherlands.
[4] Amsterdam UMC location University of Amsterdam, Department of Public and Occupational Health, Meibergdreef 9, Amsterdam, The Netherlands.
[5] Amsterdam Public Health, Health Behaviors & Chronic Diseases, Amsterdam, The Netherlands.
[6] Amsterdam UMC location Vrije Universiteit Amsterdam, Department of Obstetrics and Gynaecology, Meibergdreef 9, Amsterdam, The Netherlands.
[7] Amsterdam UMC location University of Amsterdam, Department of Vascular Medicine, Meibergdreef 9, Amsterdam, The Netherlands.
[8] Amsterdam Cardiovascular Sciences, Atherosclerosis and Ischemic Syndromes, Amsterdam, the Netherlands.
[†] These authors share first authorship.

Table of content

Table S1a: Search strategy A, Ovid Embase page 3

Table S1b: Search strategy A, Ovid Medline page 4

Table S2a: Search strategy B, Ovid Embase page 5

Table S2b: Search strategy B, Ovid Medline page 6

Figure S1: Flowchart A page 7

Figure S2: Flowchart B page 8

Table S3: Studies on prevalence / incidence and outcome of HDP in non-White populations identified in the systematic search. page 9

Table S4: Studies on CVD and CVD risk factors after HDP in non-White populations identified in the systematic search. page 21

Table S5: Prevalence / incidence and outcome of HDP in non-White populations. page 37

Table S6a: Risk of hypertension after HDP in non-White populations. page 74

Table S6b: Risk of type 2 diabetes mellitus and prediabetes after HDP in non-White populations. page 80

Table S6c: Risk of dyslipidemia after HDP in non-White populations. page 83

Table S6d: Risk of chronic kidney disease after HDP in non-White populations. page 86

Table S6e: Risk of metabolic syndrome after HDP in non-White populations. page 89

Table S6f: Risk of cardiovascular disease after HDP in non-White populations. page 92

Table S6g: Risk of cerebrovascular accidents (stroke) after HDP in non-White populations. page 94

Table S6h: Risk of ischemic heart disease after HDP in non-White populations. page 97

Table S1a: Search strategy A, Ovid Embase, 1947 to 2022 January 26		
1	(ethnic* or ethno* or race* or racial* or ancestr* or minorit*).ti,kf.	133211
2	(migrant* or immigrant* or emigrant* or displaced* or refugee* or asylum seeker*).ti,kf.	41152
3	(Asian* or African* or Caribbean* or Antillean* or Hindustani* or Afro* or Black* or Hispanic* or Latino* or Mexican* or Latina* or Latinx* or Pacific Island* or Australoid* or Indigenous* or Amerindian* or ((American* or Canadian* or Alaska* of Australian* or Hawaiian*) adj3 (Indian* or Native* or Indigenous* or Aborigin* or First Nation* or Filipino*)) or Creol* or Turkish* or Moroccan* or Surinamese* or Antill* or Arab* or Indian* or Pakistani* or Bangladeshi* or Chinese* or Middle East* or Mediterranean* or non-White* or non-European or non-Caucasian or non-Western* or BAME* or BIPOC* or colo?red or "of colo?r" or creol* or mestiz* or metis* or mulatto* or multiracial* or biracial*).ti,kf.	596223
4	exp "ethnic or racial aspects"/ or exp ethnic group/ or exp minority group/ or ancestry group/ or exp asian american/ or exp asian continental ancestry group/ or exp australoid/ or exp black person/ or exp british asian/ or exp hispanic/ or exp indigenous people/ or exp migrant/ or exp mongoloid/ or exp multiracial person/ or exp oceanic ancestry group/	643797
5	or/1-4	1177549
6	exp eclampsia/ep, et or exp preeclampsia/ep, et or (eclamp* or pre?clamp* or gestosis or ((pregnancy or gestation* or gravid*) adj2 (tox?emi* or toxicos*))).ti,kf. or exp maternal hypertension/ep, et or ((pregnancy or gestation* or maternal) adj4 hypertensi*).ti,kf. or ("h?emolys* elevated liver enzyme* and low platelet*" or hellp*).ti,kf.	49860
7	5 and 6	2045
8	(animal experiment or animal model or rat or rats or mouse or mice).ti,kf,od.	4950936
9	(drug safety or pharmacogenetic testing or pharmacogenomics).od.	488910
10	case report.ti.	355645
11	7 not (8 or 9 or 10)	1989
**12**	**limit 11 to English language**	**1933**

Table S1b: Search strategy A, Ovid Medline, 1947 to 2022 January 26		
1	(ethnic* or ethno* or race* or racial* or ancestr* or minorit*).ti,kf.	103001
2	(migrant* or immigrant* or emigrant* or displaced* or refugee* or asylum seeker*).ti,kf.	38075
3	(Asian* or African* or Caribbean* or Antillean* or Hindustani* or Afro* or Black* or Hispanic* or Latino* or Latina* or Latinx* or Pacific Island* or Australoid* or Indigenous* or Amerindian* or ((American* or Canadian* or Alaska* of Australian*) adj3 (Indian* or Native* or Indigenous* or Aborigin* or First Nation* or Filipino*)) or non-White* or non-European or non-Caucasian or Middle East* or Mediterranean* or BAME* or colo?red or "of colo?r" or creol* or mestiz* or metis* or mulatto* or multiracial* or biracial*).ti,kf.	259284
4	exp "emigrants and immigrants"/ or exp ethnic groups/ or exp refugees/	188287
5	or/1-4	477710
6	exp Hypertension, Pregnancy-Induced/ep, et or (eclamp* or pre?clamp* or gestosis or ((pregnancy or gestation* or gravid*) adj2 (tox?emi* or toxicos*))).ti,kf. or ((pregnancy or gestation* or maternal) adj4 hypertensi*).ti,kf. or ("h?emolys* elevated liver enzyme* and low platelet*" or hellp*).ti,kf.	37876
7	5 and 6	522
8	(animal experiment or animal model or rat or rats or mouse or mice).ti,kf.	1444480
9	case report.ti.	272249
10	7 not (8 or 9)	519
**11**	**limit 10 to English language**	**497**

Table S2a: Search strategy B, Ovid Embase, 1947 to 2022 February 08		
1	exp eclampsia/ or exp preeclampsia/ or (eclamp* or pre?clamp* or gestosis or ((pregnancy or gestation* or gravid*) adj2 (tox?emi* or toxicos*))).ti,kf. or ((pregnancy or gestation* or maternal) adj4 hypertensi*).ti,kf. or exp maternal hypertension/ or ("h?emolys* elevated liver enzyme* and low platelet*" or hellp*).ti,kf.	90161
2	exp animal/ not exp human/	5690597
3	case report.ti.	356880
4	review.ti.	695490
5	exp abstract report/ or exp book/ or exp editorial/ or exp letter/ or exp note/ or exp "review"/ or exp short survey/	6277353
6	(wom?n* or mother* or maternal*).ti,ab.	2260354
7	cardiovascular disease/ or heart disease/ep, et or exp heart death/ep, et or exp heart failure/ep, et or exp ischemic heart disease/ep, et or exp major adverse cardiac event/ep, et or exp myocardial disease/ep, et or exp vascular disease/ep, et or exp cardiovascular mortality/ep, et or (cardiovascular* or cerebrocardiovascular* or cardiocerebrovascular* or cardiometabolic* or metabol*).ti,kf. or ((myocard* adj2 infact*) or (isch?emi* adj2 (event* or attack* or cardi* or myocardi* or brain* or cerebr*)) or atherosclero* or arteriosclero* or "vascular calcification*" or (coronar* adj3 (disease* or syndrome*)) or "angina pectoris").ti,kf. or (stroke or CVA or TIA or cerebrovascular*).ti,kf. or (((cardiovascular* or heart* or cardiac* or myocardi* or ventric*) adj3 (dysfunction* or failure* or decompens* or insufficien* or hypertroph*)) or "decompensatio cordis").ti,kf.	2030299
8	hypertension/ep, et or exp essential hypertension/ep, et or exp systolic hypertension/ep, et or (hypertensi* not ("pulmonary hypertens*" or "gestation?? hypertens*" or "maternal hypertens*" or "pregnan?? hypertens*" or "hypertension in pregnan*" or "hypertension of pregnan*" or "hypertension during pregnanc*" or "pregnancy induced hypertension" or "hypertensi?? disorder? of pregnan*" or "hypertensi?? disease? of pregnan*" or "hypertensi?? disorder? of gestation*" or "hypertensi?? disease? of gestation*" or "non hypertensi*" or "non-hypertensi*")).ti,kf. or exp non-insuline dependent diabetes mellitus/ep, et or ((diabet* or T2DM) not ("gestation?? diabet*" or "pregnan?? diabet*" or "diabet?? in pregnanc*" or "diabet?? during pregnanc*" or "diabetes of pregnan*" or "diabet?? in gestation*" or "diabet?? during gestation*" or "diabetes of gestation*or diabet?? mellitus in pregnanc*" or "diabet?? mellitus during pregnanc*" or "diabetes mellitus of pregnan*" or "diabet?? mellitus in gestation*" or "diabet?? mellitus during gestation*" or "diabetes mellitus of gestation*or non diabet*" or "non-diabet*")).ti,kf. or exp dyslipidemia/ep, et or exp hyperlipidemia/ep, et or (dyslipid?em* or hyperlipid?em* or hypercholester?em* or hypercholesterol?em* or hypercholesterin?em* hypertriglycerid?em*).ti,kf. or exp metabolic syndrome X/ep, et or ((metabol* or "insuline resistence") adj2 syndrome*).ti,kf. or exp chronic kidney failure/ep, et or ((renal or kidney) adj3 (insufficienc* or failure* or dysfuction*)).ti,kf.	1037710
9	7 or 8	2845732
10	9 and 1	14299
11	10 not (2 or 3 or 4 or 5)	9745
12	limit 11 to english language	8532
13	12 and 6	6734
14	(animal experiment or animal model or rat or rats or mouse or mice).ti,kf,od.	4958924
**15**	**13 not 14**	**6535**

Table S2b: Search strategy B, Ovid Medline, 1946 to February 08, 2022		
1	cardiovascular diseases/ep, et or heart diseases/ep, et or exp cardiac output, low/ep, et or exp cardiomegaly/ep, et or exp cardiomyopathies/ep, et or exp heart arrest/ep, et or exp heart failure/ep, et or exp heart rupture/ep, et or exp myocardial ischemia/ep, et or exp myocardial stunning/ep, et or exp post-cardiac arrest syndrome/ep, et or exp ventricular dysfunction/ep, et or vascular diseases/ep, et or exp aneurysm/ep, et or exp aortic diseases/ep, et or exp arterial occlusive diseases/ep, et or cerebrovascular disorders/ep, et or exp basal ganglia cerebrovascular disease/ep, et or exp brain ischemia/ep, et or exp carotid artery diseases/ep, et or exp cerebral small vessel diseases/ep, et or exp cerebrovascular trauma/ep, et or exp intracranial arterial diseases/ep, et or exp "intracranial embolism and thrombosis"/ep, et or exp intracranial hemorrhages/ep, et or exp stroke/ep, et or exp "embolism and thrombosis"/ep, et or exp peripheral vascular diseases/ep, et	476019
2	(cardiovascular* or cerebrocardiovascular* or cardiocerebrovascular* or cardiometabolic* or metabol*).ti,kf. or ((myocard* adj2 infact*) or (isch?emi* adj2 (event* or attack* or cardi* or myocardi* or brain* or cerebr*)) or atherosclero* or arteriosclero* or "vascular calcification*" or (coronar* adj3 (disease* or syndrome*)) or "angina pectoris").ti,ab. or (stroke or CVA or TIA or cerebrovascular*).ti,kf. or (((cardiovascular* or heart* or cardiac* or myocardi* or ventric*) adj3 (dysfunction* or failure* or decompens* or insufficien* or hypertroph*)) or "decompensatio cordis").ti,kf.	1395828
3	hypertension/ep, et or exp essential hypertension/ep, et or diabetes mellitus/ep, et or exp diabetes complications/ep, et or exp diabetes mellitus, type 2/ep, et or lipid metabolism disorders/ep, et or exp dyslipidemias/ep, et or metabolic diseases/ep, et or exp metabolic syndrome/ep, et or renal insufficiency/ep, et or exp renal insufficiency, chronic/ep, et	178661
4	((hypertensi* not ("pulmonary hypertens*" or "gestation?? hypertens*" or "maternal hypertens*" or "pregnan?? hypertens*" or "hypertension in pregnan*" or "hypertension of pregnan*" or "hypertension during pregnanc*" or "pregnancy induced hypertension" or "hypertensi?? disorder? of pregnan*" or "hypertensi?? disease? of pregnan*" or "hypertensi?? disorder? of gestation*" or "hypertensi?? disease? of gestation*" or "non hypertensi*" or "non-hypertensi*")) or ((diabet* or T2DM) not ("gestation?? diabet*" or "pregnan?? diabet*" or "diabet?? in pregnanc*" or "diabet?? during pregnanc*" or "diabetes of pregnan*" or "diabet?? in gestation*" or "diabet?? during gestation*" or "diabetes of gestation*or diabet?? mellitus in pregnanc*" or "diabet?? mellitus during pregnanc*" or "diabetes mellitus of pregnan*" or "diabet?? mellitus in gestation*" or "diabet?? mellitus during gestation*" or "diabetes mellitus of gestation*or non diabet*" or "non-diabet*")) or (dyslipid?em* or hyperlipid?em* or hypercholester?em* or hypercholesterol?em* or hypercholesterin?em* hypertriglycerid?em*) or ((metabol* or "insuline resistence") adj2 syndrome*) or ((renal or kidney) adj3 (insufficienc* or failure* or dysfuction*))).ti,kf.	689768
5	or/1-4	2272968
6	exp Hypertension, Pregnancy-Induced/ep, et	8370
7	(eclamp* or pre?clamp* or gestosis or ((pregnancy or gestation* or gravid*) adj2 (tox?emi* or toxicos*)) or ((pregnancy or gestation* or maternal) adj4 hypertensi*) or ("h?emolys* elevated liver enzyme* and low platelet*" or hellp*)).ti,kf.	35095
8	or/6-7	37955
9	5 and 8	6575
10	case report.ti.	273225
11	review.ti.	585300
12	exp Animals/ not exp Humans/	4956216
13	(wom?n* or mother* or maternal*).ti,ab.	1600846
14	9 not (10 or 11 or 12)	6083
15	14 and 13	4106
16	(animal experiment or animal model or rat or rats or mouse or mice).ti,kf.	1445918
17	15 not 16	4074
18	limit 17 to english language	3722
19	(review or systematic review or meta analysis or comment or editorial or letter).pt.	5068604
**20**	**18 not 19**	**3121**

Figure S1: Flowchart A

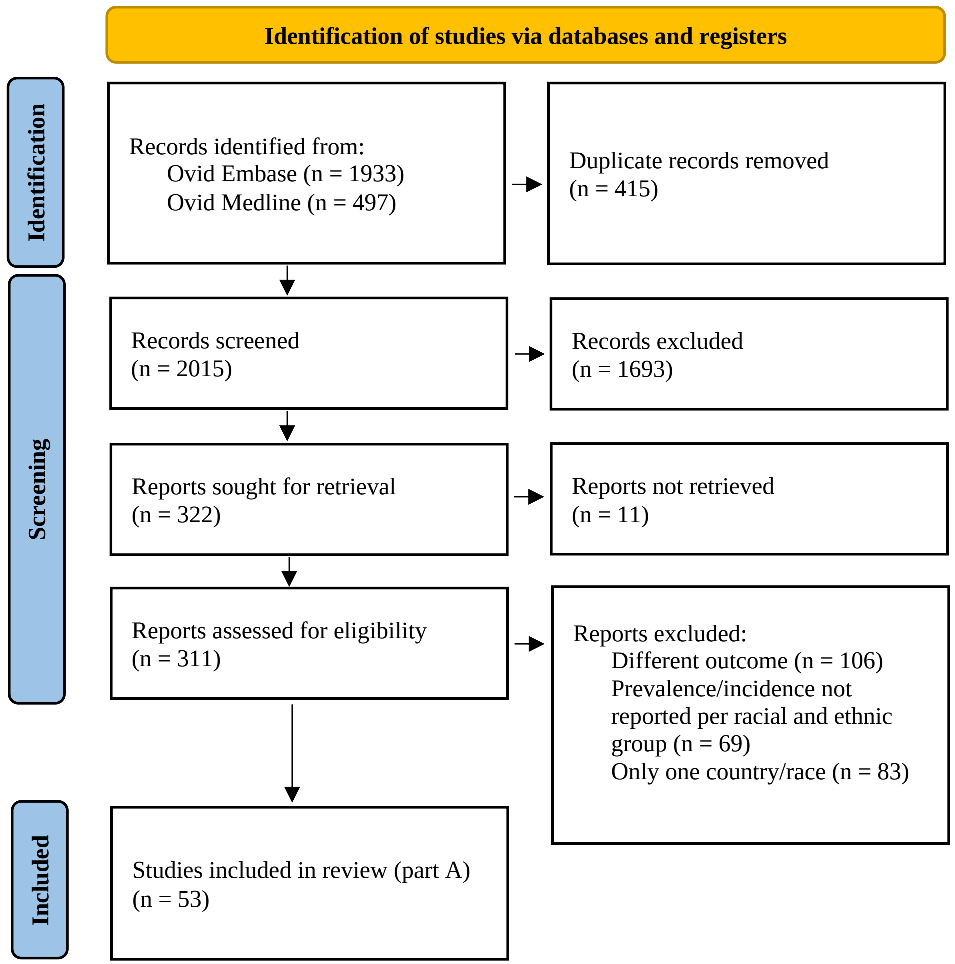

From: Page MJ, McKenzie JE, Bossuyt PM, Boutron I, Hoffmann TC, Mulrow CD, et al. The PRISMA 2020 statement: an updated guideline for reporting systematic reviews. BMJ 2021;372:n71. doi: 10.1136/bmj.n71. For more information, visit: http://www.prisma-statement.org/

Figure S2: Flowchart B

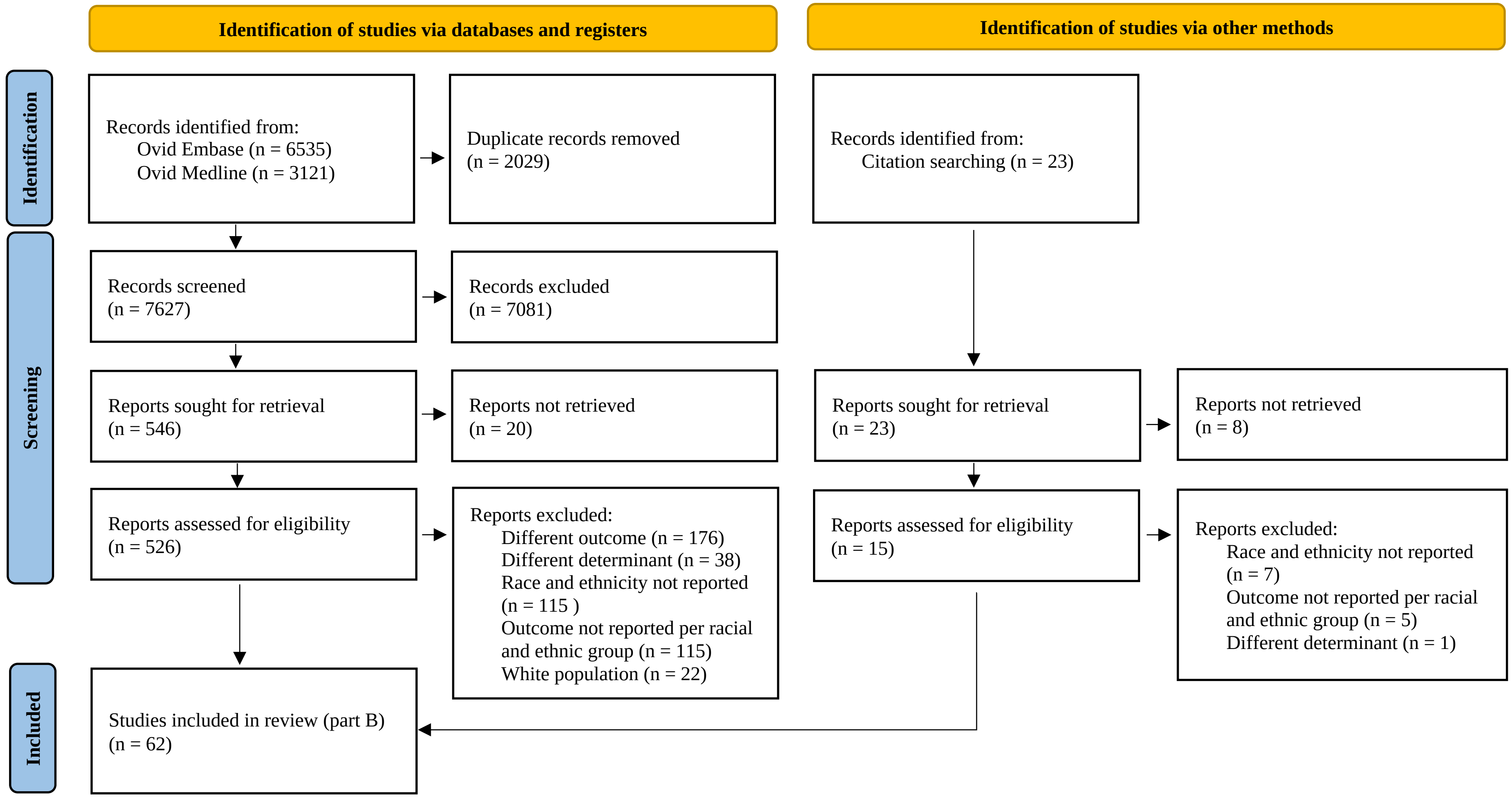

From: Page MJ, McKenzie JE, Bossuyt PM, Boutron I, Hoffmann TC, Mulrow CD, et al. The PRISMA 2020 statement: an updated guideline for reporting systematic reviews. BMJ 2021;372:n71. doi: 10.1136/bmj.n71. For more information, visit: http://www.prisma-statement.org/

Table S3: Studies on prevalence / incidence and outcome of HDP in non-White populations identified in the systematic search.

Author (year)	Country	Race, ethnicity or origin	Study period	Study design	Inclusion criteria	Exclusion criteria	Type of HDP	Definitions of HDP	Definition of race and ethnicity.	Analysis	Adjustment factors
**ASIA**											
Loi (2007)	Singapore	Chinese, Malay, Indian, others	2000-2001	Retrospective cohort study	All deliveries in KK hospital.	NR	Sev PE	Sev PE: BP ≥ 170/110 mmHg with proteinuria ≥ 0.3 g/day or 2 + on urine dipstick, and/or severe biochemical abnormalities. Cases with E were also included.	Not provided.	Chi-squared or Fisher's exact tests.	N/a.
Tan (2006)	Singapore	Chinese, Malay, Indian, others	1999-2003	Retrospective cohort study	All deliveries in KK hospital.	NR	PE	ICD-9-CM.	Not provided.	Chi-squared tests.	N/a.
**EUROPE**											
Bastola (2022)	Finland	Finland, Western high income, Eastern Europe, Russia, South Asia, East Asia Sub-Saharan, Middle East / North Africa, Latin America / Caribbean	2004-2014	Retrospective study	Most recent singleton birth of women who delivered between 2004 and 2014.	Multiple births, women with unknown country of origin.	HDP, GH, PE	ICD-10.	Race and ethnicity based on maternal country of origin.	Generalized linear models.	Maternal age, socioeconomic position, smoking in pregnancy, parity, pre-pregnancy BMI, preexisting diabetes, delivery year.
Siddiqui (2020)	France	Europe, North Africa, Sub-Saharan Africa, other	2010-2011	Prospective cohort study	Women who delivered during the study period in one of the four participating hospitals in Paris, located in predominantly low-income neighborhoods.	Age < 18y, termination of pregnancy < 20 weeks, lost to follow up, missing data.	Sev PE	Severe PE: BP > 160/110 mmHg, proteinuria > 3.5g/24h, serum creatinine >100 μmol/l, urine output < 20 ml/h, hemolysis, liver transaminitis >3 times upper limit of normal, thrombocytopenia < 100.000/mm3, or gestational age < 32 weeks at onset of disease.	Race and ethnicity based on country of birth.	Multivariate logistic regression analysis.	Age, parity, social deprivation.
Sole (2018)	Norway	Norway, Europe (EEA), Europe (non-EEA), North America, Latin	1999-2014	Population-based cohort study	Singleton deliveries ≥ 23 wk and <44 gestational weeks	Major fetal anomalies.	GH, PE	PE: BP ≥ 140/90 after 20 weeks of gestation with proteinuria ≥ 0.3g/24h or	Race and ethnicity based on country of birth.	Logistic regression analysis.	Education, age, consanguinity, diabetes, smoking status.

		America / Caribbean, Middle East / North Africa, Sub-Saharan Africa, Transcaucasia / Central Asia, South Asia, East Asia Pacific and Oceania			during the study period.			PCR > 0.3 or ≥1+ on urine dipstick test.<br>GH: BP ≥ 140/90 after 20 weeks of gestation without proteinuria, or unspecified maternal hypertension not diagnosed as CH, pre-eclampsia, eclampsia or HELLP syndrome.			
Nilsen (2018)	Norway	Norway, Vietnam, China, Iran, Morocco, Great Britain, Lithuania, Thailand, Germany, Netherlands, US, Ukraine, Iraq, Afghanistan, Romania, Former Yugoslavia, Latvia, Turkey, Sri Lanka, Denmark, Pakistan, Poland, Russia, Estonia, Eritrea, Brazil, India, Finland, Myanmar, Chile, Kenya, Sweden, Ethiopia, Somalia, Philippines, Ghana, Iceland, Nigeria, Burundi, Congo, Bangladesh, Tanzania	1990-2013	Population-based study	Norwegian women and first-generation immigrant women with a singleton pregnancy during the study period in Norway.	Second-generation immigrants, adoptees, mixed-ethnic parents, missing data on immigration reason.	PE	ICD-8/9.	Race and ethnicity based on country of birth. Data on immigration reason recorded (refuge, family (reunion or establishment), labour, education, and unspecified reasons).	Logistic regression analysis.	Year of birth, maternal age at birth, parity, marital status at birth, CH, pre-pregnancy diabetes, maternal income, education.
Panaitescu (2017)	UK	Caucasian, African Caribbean, South Asia, East Asia, Mixed	2007-2015	Prospective study	Pregnant women attending their first routine hospital visit at King's College Hospital, London, UK (between March 2006 and July 2015) and Medway Maritime Hospital, Gillingham, UK (between February	Fetal aneuploidy or major defects, pregnancies ending in miscarriage < 16 weeks' gestation, those ending in termination for psychosocial reasons.	CH	ISSHP 2001 classification.	Self-reported.	Multivariable logistic regression analysis.	Maternal factors, medical history, obstetric history.

					2007 and November 2015).						
Naimy (2015)	Norway	Norway, Pakistan, Vietnam, Somalia, Sri Lanka, Philippines, Iraq, Thailand, Afghanistan	1986-2005	Population-based cohort study	Women who delivered > 20 weeks of gestation during the study period.	Missing data on age, birthweight, GA at delivery.	PE	PE: BP ≥ 140/90 after 20 weeks of gestation with proteinuria ≥ 0.3g/24h or ≥1+ on urine dipstick test.	Race and ethnicity based on country of birth.	Logistic regression analysis.	Maternal age at delivery, parity (in all women), multifetal pregnancy, year of delivery, diabetes status.
Bouthoorn (2012)	The Netherlands	Dutch, Surinamese-Creoles, Surinamese-Hindustani, Turkish, Moroccan, Cape Verdean, Antillean	2002-2006	Population-based cohort study	Live singleton births in the Generation R study.	Missing data on ethnic background.	GE, PE	GH: BP ≥ 140/90 mmHg after 20 weeks of gestation in previously normotensive women.<br>PE: BP ≥ 140/90 mmHg after 20 weeks of gestation in previously normotensive women, with presence of proteinuria (≥ 2 dipstick readings of ≥ 2+, 1 catheter sample reading of ≥1+, or a 24-hour urine collection containing ≥ 300 mg of protein).	Race and ethnicity defined by country of birth of the mother and her parents.	Logistic regression analysis.	GA at visit, education, age, parity, smoking during pregnancy, alcohol use during pregnancy, caffeine intake during pregnancy, sodium intake, energy intake, folium acid use, BMI, maternal stress index.
Zwart (2008)	The Netherlands	Netherlands, Morocco / Turkey, Surinam / Dutch Antilles, Other Non-Western, other Western	2004-2006	Population-based cohort study	All pregnancies in the Netherlands during the study period.	Unknown BP on admission, seizures caused by another illness than E.	E	Eclampsia was defined as the occurrence of convulsions superimposed on PE and not attributable to other causes.	Not provided.	Chi-squared test.	N/a.
Lydakis (1998)	UK	White, Black / African Caribbean, Indo-Asian	1980-1997	Retrospective cohort study	Women referred to Birmingham City Hospital due to CH, GH or PE in a previous pregnancy, or high BP in first weeks of current pregnancy.	DM, renal diseases, secondary forms of HT, GA <20 weeks.	CH, sPE	ISSHP 1993 classification.	Not provided.	Numbers and percentages, RR.	N/a.
**NORTH AFRICA AND THE MIDDLE EAST**											

Segal (1996)	Israel	Ethiopian, Israeli	1981-1988	Retrospective cohort study	All singleton births among Ethiopian immigrant women and control group of Israeli women.	NR	PE	NR	Race and ethnicity based on maternal country of origin.	Chi squared tests.	N/a.
**NORTH AMERICA**											
Boakye (2021)	US	Hispanic, NHB, NHW	1998-2016	Retrospective cohort study	Live singleton births at the Boston Medical Center.	Multiple gestations, pregnancies resulting from IVF, neonates with chromosomal abnormalities, and major birth defect.	CH, PE	ICD-9/10.	Self -reported.	Chi-squared test, Fischer's exact test, logistic regression analysis.	Age, educational level, marital status, stress, CH, pre-existing DM, GDM, parity, smoking, BMI.
Gyamfi-Bannerman (2021)	US	NHW, NHB, Hispanic, Asian or Pacific Islander, Native American, other	2012-2014	Retrospective cohort study	All nationwide deliveries of women aged 15-54 years with a diagnosis of PE.	NR	PE	ICD 9.	Self-reported maternal race and ethnicity, categorized by the Agency for Healthcare Research and Quality.	Chi-squared test, log-linear regression model.	Year, bed size, insurance status, hospital location, income quartile, hospital region, hospital teaching status, maternal age, race.
Shah (2021)	US	NHW, NHB, Asian Indian, Chinese, Filipino, Japanese, Korean, Vietnamese, Mexican, Puerto Rican, Cuban, Central / South American	2014-2019	Population based cohort study	All live singleton births to women in the US within the study period.	Age < 15y or > 44y, non-US residents, missing data.	CH, GH/PE	CH and GH/PE not defined.	Self-reported.	Numbers, percentages, and age-standardized rate ratios.	Age.
Ananth (2021)	US	White, Black	1979-2018	Population-based cohort study	Live births in the US during the study period.	Age <15y or >49y.	HDP	ICD-9/10.	Not provided.	Rates.	N/a.
Maric (2019)	US	NHW, NH African American, Asian, Hispanic	2007-2011	Retrospective cohort study	California singleton live births.	GA < 20 or > 41 weeks, pre-pregnancy weight < 75 or > 450 lbs, height < 52 or > 76 inches, missing GA, pre-pregnancy weight, or height information.	PE, E	ICD-9-CM.	Not provided.	Logistic regression	Age, pre-pregnancy BMI, education, parity, prenatal care, medical payer, smoking, maternal birth country.

Ananth (2019)	US	Black, White	1970-2010	Retrospective cohort study	In-hospital deliveries with selected NHDS data in the US.	Age <15y or ≥ 50y.	CH	ICD-8/9.	Not provided.	Numbers, percentages, RR.	Obesity, smoking.
Hu (2019)	US	Black, White	2005-2014	Retrospective cohort study	All registered live births in Florida.	Births with maternal residential addresses outside Florida.	GH/PE	GH: development of hypertension after 20 weeks of pregnancy.<br>PE: new onset of hypertension and proteinuria after 20 weeks of pregnancy.	Not provided.	Number, percentages, RR.	Age, education, smoking during pregnancy, pre-pregnancy BMI, year of conception.
Singh (2018)	US	NHW, NHB, American, AI/AN, Chinese, Japanese, Filipino, Hawaiian, Asian Indian, Korean, Vietnamese, Samoan, Other Asian / Pacific Islander, Mexican, Puerto Rican, Cuban, Central & South American, other Hispanic	2014-2015	Population-based cohort study	All women who delivered in the US during the study period with information on HDP available.	NR	HDP, GH/PE, CH, E	Maternal hypertension: BP ≥ 140/90 mmHg during pregnancy.	Race and ethnicity based on country of origin / nativity.	Multivariable logistic regression analysis.	Maternal age, marital status, plurality, maternal education, place and region of residence, GDM, pre-pregnancy BMI, GWG.
Zamora (2016)	US	White, American Indian / Alaska Native	2003-2013	Retrospective cohort study	Singleton live births to first-time mothers in Washington State.	NR	PE	ICD-9.	Self -reported.	Logistic regression analysis.	Birth year, maternal age, educational attainment, marital status, Medicaid insurance, WIC participation, prenatal smoking, BMI.
Haghigh at 2016	US	Asian, Black, Hispanic, NHW, other, unknown	2001-2006	Retrospective cohort study	All deliveries in four hospitals in Los Angeles, Orange Counties and California Hospital based data matched to birth certificate.	NR	PE	Onset of hypertension and the presence of protein in the urine at >20 weeks' gestation in a previously normotensive woman.	Not provided.	Chi-squared test.	N/a.
Masho (2016)	US	NHW, NHB, Hispanic, other	2004-2011	Retrospective cohort study	Singleton live births national.	NR	HDP	Survey based. Definition of HDP not provided.	Self-reported.	Logistic regression and analysis.	Socio-demographic, biologic, behavioral risk factors.

		non-Hispanic									
Nakagawa (2016)	Hawaii	Chinese, Filipino, Native Hawaiian, Japanese, Other Asian, Other Pacific Islanders, other White	1995-2013	Retrospective cohort study	All-in hospital deliveries in Hawaii.	Non-Hawaii residents giving birth in Hawaii, missing race/ethnicity.	CH, PE	ICD-9.	Self-reported.	Multivariable logistic regression analysis.	Age, insurance type, location of residence, multiple gestation, multiparity, CH, pre-gestational DM, smoking, obesity.
Shahul (2015)	US	White, Black, Hispanic	2004-2012	Retrospective cohort study	All patients with a diagnosis of PE/E.	NR	PE	ICD-9-CM.	Self-reported maternal race and ethnicity, categorized by the Agency for Healthcare Research and Quality.	Multivariate logistic regression analysis.	Age, parity, PTB, delivery type, diabetes, GDM, obesity, household income, payer type, region, hospital type.
Chang (2014)	US	NHW, NHB, NH American Indian, NH Asian / Pacific Islander, Hispanic	2010	Retrospective cohort study	Singleton pregnancies between 20 and 44 weeks of gestation.	Major fetal anomalies.	GH/PE, CH, E	GH/PE: hypertension after the 20th week of gestation that resulted in an increase in SBP ≥ 30 mmHg or DBP ≥ 15 mmHg on two measurements taken 6 hours apart. Eclampsia: occurrence of convulsions and/or coma unrelated to other cerebral conditions in women with signs and symptoms of PE.	Self-reported.	Numbers, percentages.	N/a.
Ghosh (2014)	US	NHW, NHB, Hispanic, Asian / Pacific Islander, Multiracial/other	2002-2008	Retrospective cohort study	Singleton births from nulliparous women.	Missing data on race, BMI, marital status, maternal age.	CH, GH, PE, E, sPE	ICD-9.	Race and ethnicity based and standards for federal statistics and administrative reporting.	Multivariable logistic regression analysis.	Maternal age, pre - pregnancy BMI, insurance status, smoking during pregnancy, marital status.
Liu (2014)	US	NHW, NHB, Hispanic	2004-2006	Retrospective cohort study	Live, singleton births between 20-44 weeks with a birthweight > 500 grams to mothers without CH. (South Carolina)	Missing data on race, pre-pregnancy weight, height or BMI, pre-pregnancy BMI <10 or > 80; missing data on GWG, GWG > 97 pounds,	GH/PE	Not defined.	Not provided.	Multivariable logistic regression analysis.	Parity, maternal education, smoking during pregnancy, month prenatal care started, maternal age, marital status.

						missing information for other covariates.					
Marshall (2014)	US	African American, Caucasian	2000-2006	Retrospective cohort study	All liveborn singleton > 37 weeks with BMI > 30.	Major congenital anomalies, diabetes, CH.	PE	NR	Race and ethnicity determined from Missouri Vital Records data.	Multivariable logistic regression analysis.	Maternal age, education, parity Medicaid status, adequate prenatal care, smoking status, married versus single, prior CS, primary elective CS, infant gender.
Gold (2014)	US	Hispanic, NHW, NHB	2004-2008 (GH/PE) 1997-2009 (E)	Retrospective cohort study	All pregnancies in the US.	CH, DM.	GH/PE, E	NR	Not provided.	Spearman's rank correlation test.	N/a.
Breathett (2014)	US	African American, Caucasian	1979-2006	Retrospective study	All deliveries in African American and Caucasian women.	Other racial groups.	PE	ICD-9.	Not provided.	Poisson regression analysis.	Age, geographic region, DM, HT, MI, HF, transient HT, GDM.
Lisonkova (2013)	US	NHW, African American, Hispanic, Native American, other	2003-2008	Retrospective cohort study	All singleton deliveries in Washington State.	GA <20 weeks, missing data on GA, unsuccessful data-linkage.	PE	ICD-9.	Not provided.	Cox proportional hazards regression analysis.	Race, parity, maternal age, maternal education, infant's sex, marital status, infertility treatment, CH, DM, congenital anomalies
Fong (2013)	US	Caucasian, Black, Native American / Eskimo / Aleut, Asian / Pacific Islander, Hispanic	2001-2007	Retrospective cohort study	All deliveries in California.	Age < 15y or > 55y, missing data on race and ethnicity or age.	E	ICD-9.	Not provided.	Multivariable logistic regression analysis.	Age, insurance type, year of delivery, obesity, DM, cardiac disease, asthma, renal disease, urinary tract infection, multiple gestations, tobacco use.
Cripe (2012)	US	Cambodian, Laotian, Vietnamese, Japanese, White	1993-2006	Retrospective cohort study	All singleton deliveries in Washington State.	Missing data on race, mothers with White or Black race born in any of these four Asian countries, cases	PE	ICD-9.	Race and ethnicity based on maternal country of birth.	Chi-square test.	N/a.

						with both GDM and PE, those with missing information for GDM and PE, incomplete linkage to birth certificates.					
Gong (2012)	US	NHW, African American, North Africa, Sub-Saharan Africa, East Asia, Southeast Asia and Pacific Islands, South Central Asia, NH Caribbean, Hispanic Caribbean, Mexico, South America, Central America, other Hispanic	1995-2003	Retrospective cohort study	Singleton births ≥ 20 weeks of gestation in New York City during the study period.	CH, pre-existing DM, chronic renal disease, missing variables.	PE	PE: BP ≥ 140/90 after 20 weeks of gestation with proteinuria.<br>ICD-9 codes.	Race and ethnicity based on maternal ancestry and maternal country of birth.	Logistic regression analysis.	Maternal age, education, parity, self-reported pre-pregnancy weight, smoking during pregnancy, year of delivery.
Miranda (2010)	US	NHW, NHB, Hispanic	1994-2003	Retrospective study	Singleton live births ≥24 weeks of gestation and birthweight ≥400 grams in North Carolina.	Congenital anomalies, any other maternal medical complications (i.e. DM, CH).	GH/PE	Not defined.	Self-reported.	Multivariable logistic regression analysis.	Maternal age, race.
Wong (2008)	US	Chinese, Japanese, Filipino, Indian, Korean, Vietnamese, Samoan, Guamanian, Hawaiian	2003	Retrospective cohort study	All deliveries in women from the defined racial or ethnic groups that were documented by US birth certificates during the year 2003.	Individuals who identified themselves as “other Asian/Pacific Islanders” or “combined other Asian/Pacific Islander” and those who identified themselves of both Hispanic and Asian race.	GH/PE, E	NR	Self-reported, based on 1997 revisions to the Standards for the classification of Federal Data on Race and Ethnicity.	Multivariable logistic regression analysis.	Maternal age, parity, marriage status, foreign status, education, alcohol, tobacco use, GA diabetes, previous infant with birthweight > 4000 gram, previous SGA, DM, CH, HDP.
Yeo (2007)	US	Hispanic, NH	1998	Retrospective study	All hospital deliveries in Detroit.	NR	GH/PE	GH/PE: BP ≥ 140/90 mmHg after 20 weeks GA in woman with previously normal BP with or without proteinuria.	Not provided.	Chi-squared test.	N/a.

Tanaka (2007)	US	White, Hispanic, Black, other	1993-2002	Retrospective cohort study	All hospital deliveries in New York State.	Age < 15y or > 54y, missing data on age or residential information, miscarriage, spontaneous or induced abortion, HIV, AIDS, DM.	CH, GH, PE, sPE	ICD-9-CM.	Race and ethnicity based on hospital discharge records.	Logistic regression analysis.	SES.
Rao (2006-2)	US	Chinese, Filipino, Indian / Pakistani, Japanese, Korean, Pacific Islander (Tonga, Samoa, Guam, and Polynesia), Vietnamese	1998-2003	Retrospective cohort study	All singleton deliveries in Sandford Medical Center (California).	Multiples, birthweight <500 g, multiple ethnicity designations.	GH/PE	Not defined.	Self-reported.	Multivariate regression analysis.	Maternal age, maternal education, live birth order, extent of prenatal care, insurance status, GDM.
Rao (2006-1)	US	Japanese, Chinese, Filipino	1985-2001	Retrospective cohort study	All deliveries at the University of California.	NR	CH, PE	Not defined.	Self-reported.	Multivariate logistic regression analysis.	Maternal age, parity, obesity, insurance status, marital status, CH.
Odell (2006)	US	Haitian, African American	1996-2000	Population-based cohort study	African American and Haitian women who delivered in Massachusetts during the study period.	Birthweight <350g, >5999g, <20 weeks of gestation, missing data.	CH, PE	ICD-9.	Self-reported.	Percentages.	N/a.
Bryant (2005)	US	Black, White	1998	Retrospective cohort study	All women with singleton pregnancies delivered at the Brigham and Women's Hospital.	Hispanic and other races, multiple gestations, pregestational DM, preexisting cardiac disease, connective tissue disorders, chronic renal disease.	HDP, CH, GH, PE, sPE	CH: HT predating pregnancy or BP >140/90 prior to 20 weeks GA. sPE: significant elevation of BP with new proteinuria (>1 + proteinuria on dipstick, or > 300 mg protein/24h). GH: BP ≥ 140/90 on at least two occasions, appearing after 20 weeks GA, without proteinuria. PE: BP ≥ 140/90 on at least two occasions at GA >20 weeks, with proteinuria as defined above.	Self-reported.	Numbers and percentages.	N/a.

								Sev PE: PE + BP ≥ 160/110 mmHg, severe headache, visual changes, epigastric pain, transaminase levels ≥2x normal value, platelet count < 100,000/mm3, serum creatinine > 1.2 mg/dL, proteinuria > 5 g/24 hours, oliguria, or pulmonary edema.			
Wolf (2004)	US	Caucasian, Hispanic	1998-2002	Prospective cohort study	All nulliparous who received prenatal care and delivered through the Massachusetts General Hospital.	Pre-existing proteinuria, CH.	GH, PE	GH: BP ≥ 140/90 mmHg after 20 weeks of gestation.<br>PE: new onset new onset BP ≥ 140/90 mmHg after 20 weeks of gestation with proteinuria (≥2+ by dipstick test or ≥ 300mg/24h).	Self-reported.	Multivariate regression analysis.	Baseline BP, BMI, smoking, GA at the first prenatal visit, multiple gestation, preexisting DM, GDM.
Zhang (2003)	US	African American, Caucasian	1988-1997	Retrospective cohort study	All deliveries nationally.	NR	Sev PE, E, sPE	ICD-9.	Not provided.	Numbers and percentages.	N/a.
Samadi (1996)	US	African American, others	1988-1992	Retrospective cohort study	All hospital deliveries in US.	Multiples.	HDP, CH, GH, PE, E	ICD-9.	Not provided.	NR	N/a.
Urquia (2012)	Canada	Industrialized nations, Central and Eastern Europe, Hispanic America, Caribbean, Sub-Saharan Africa, Middle East and North Africa, East Asia and Pacific, South Asia	2002-2009	Population-based cohort study	Women who migrated to Ontario between 1985 and 2000 and wo delivered in Ontario during the study period.	Missing data.	Sev PE	ICD-10-CM codes.<br>Serious PE: severe PE, eclampsia, or any degree of PE with concomitant PTB, IUFD or maternal hospitalization of ≥ 7d.	Race and ethnicity based on maternal country of birth.	Logistic regression analysis.	Maternal age, number of live births, multifetal pregnancy, DM status, level of formal education, place of residence, neighborhood income quintile, duration of residence in Canada, fiscal year of delivery.
**OCEANIA**											
Al-Rubaie (2020)	Australia	Australia / New Zealand (English speaking), South Asia, Middle East / Africa, South-East Asia, North-East	2011-2014	Retrospective cohort study	All women with a birth during the study period in selected hospitals.	Use of antiplatelet therapy in the first trimester, missing variables.	PE	ISSHP classification.	Race and ethnicity based on country of birth.	Multivariate logistic regression.	Autoimmune disease, CH, chronic renal disease, DM (type 1 or 2), multiple

		Asia, Southern Asia, Sub-Saharan Africa, Latin America / Caribbean, Aboriginals / Torres Strait Islanders									pregnancy, maternal age group, BMI.
Dahlen (2013)	Australia	Australia, New Zealand, England, China, Vietnam, Lebanon, Philippines, India, other countries	1/07/ 2000- 30/06/20 08	Population-based cohort study	All singleton births >400 grams or >20 weeks in Australia.	Women who smoked during pregnancy.	HDP	Not defined.	Race and ethnicity based on country of birth.	Chi-squared tests.	N/a.
Anderso n (2012)	New Zealand	European, Māori Pacific, Chinese, Indian, Other Asian, other ethnicity	2006-2009	Retrospective cohort study	Singleton pregnancies in Auckland.	Congenital anomalies, missing data.	PE	ISSHP classification.	Self-reported.	Multivariable logistic regression analysis.	Smoking status, DM, CH and pre-existing medical conditions.
Sullivan (1997)	Australia	Australian-born Vietnam-born	1991	Retrospective cohort study	All live births and stillbirths > 20 weeks of GA or > 400 grams in Southwestern Sydney.	NR	CH, PE	NR	Race and ethnicity based on country of birth.	Numbers and percentages.	N/a.
**SUB-SAHARAN AFRICA**											
Knutzen (1977)	South Africa	Black, Colored, Whites	1972	Retrospective study	All deliveries in Peninsula Maternity Services Cape town.	NR	CH, GH, PE	CH: DBP > 90 mmHg before the 20th week of pregnancy.<br>GH: DBP > 90 mmHg on ≥ 2 occasions after the 20th week of pregnancy without proteinuria.<br>PE: DBP > 90 mmHg on ≥ 2 occasions after the 20th week of pregnancy with proteinuria.	Not provided.	Numbers and percentages, chi-squared tests.	N/a.
**INTERCONTINENTAL**											
Urquia (2014)	Australia , Canada, Spain,	Eastern Europe, Western Europe, Latin America & the Caribbean,	1995-2010	Cross-country comparative study of linked population-	All deliveries within the most recent 10-year period available to	NR	PE, E	ICD-9/10 codes. Prolonged hospital stay was defined as ≥ 7 days.	Race and ethnicity based on country of birth.	Logistic regression analysis.	Maternal age, parity.

	US, Denmar k and Sweden	North Africa & Middle East, Sub-Saharan Africa, South Asia, East-Southeast Asia		based databases.	each participating center.						

Abbreviations: AI/AN: American Indian / Alaska Native; BMI: body mass index; BP: blood pressure; CH: chronic hypertension; CS: cesarean section; DBP: diastolic blood pressure; DM: diabetes mellitus; E: eclampsia; GA: gestational age; GDM: gestational diabetes mellitus; GH: gestational hypertension; GWG: gestational weight gain; HDP: hypertensive disorders of pregnancy; HELLP: hemolysis, elevated liver enzymes and low platelets; HF: heart failure; HT: hypertension; ICD: International Classification of Diseases; ISSHP: International Society for the Study of Hypertension in Pregnancy; IUFD: intrauterine fetal demise; IVF: in vitro fertilization; MI: myocardial infarction; n/a: not applicable; NH: non-Hispanic; NHB: non-Hispanic black; NHW: non-Hispanic white; NR: not reported; OR: odds ratio; PCR: protein creatinine ratio; PE: preeclampsia; PTB: preterm birth; RR: relative rate; SBP: systolic blood pressure; sev: severe; SGA: small for gestational age; sPE: preeclampsia superimposed on chronic hypertension; UK: United Kingdom; US: United States of America; WIC: The Special Supplemental Nutrition Program for Women, Infants, and Children.

Table S4: Studies on CVD and CVD risk factors after HDP in non-White populations identified in the systematic search.										
Author (year)	Country / racial and ethnic group	Study period	Study design	Inclusion criteria	Exclusion criteria	Type of HDP	Definitions of HDP	Outcome	Analysis	Adjustment factors
**ASIA**										
Ma (2018)	China	2014-2015	Prospective cohort study	Women with PE from a single tertiary hospital with follow-up data 6 weeks postpartum.	HT, DM, thyroid or liver disease before pregnancy.	Mild PE<br>Sev PE	PE: BP ≥ 140/90 mmHg and proteinuria ≥ 300mg/24h or ≥2+ on dipstick test after 20 weeks of gestation.<br>Mild PE: DBP < 110 mmHg.<br>Sev PE: DBP ≥ 110 mmHg.	HT	Chi-squared test, multivariate regression analysis.	Clinical and demographic variables.
Wang (2017)	China	Ex: 2005-2009<br>Ou: 2009 - 2011	Retrospective cohort study	Pregnant women who were diagnosed with GDM in the Tianjin Gestational Diabetes Mellitus Prevention Program between 2005 and 2009, and who finished a health survey between August 2009 and July 2011.	Age at follow-up <20y or ≥50y, FPG ≥ 7.0 mmol/L or 2hPG ≥ 11.1, chronic disease that could seriously reduce the life expectancy or the ability to participate in the trial, currently pregnant or planning to become pregnant in the next 2 years, CH.	HDP	Identified from questionnaires. HDP defined as doctors-diagnosed HT after 20 gestational weeks, including GH, PE, severe PE or eclampsia.	HT	Chi-squared test, Cox proportional hazards regression analysis.	Age, number of births in the index pregnancy, education, family income, family history of HT, smoking status, drinking status, leisure-time physical activity, sitting time, total energy intake, dietary fiber intake, dietary sodium intake, dietary potassium intake, carbohydrates intake, and energy percent of monounsaturated fat, polyunsaturated fat and saturated fat intake, pre-pregnancy BMI, weight change.
Lu (2011)	China	Ex: 2005 - 2008<br>Ou: 2009	Retrospective cohort study	Women with severe PE who delivered at Peking University Third Hospital during the study period.	Chronic HT before pregnancy, metabolic diseases such as hyperthyroidism or hypothyroidism, DM or GDM, or PCOS, or other diseases affecting the women's life such as congenital heart disease, or addiction to smoking or alcohol.	Sev PE	Severe PE: BP ≥ 160/110 mmHg and proteinuria ≥ 1000mg/24h or ≥2+ on dipstick test, elevated AST/ALT, elevated LDH, creatinine >106 umol/L, persistent headache, central nerve dysfunction, blurred vision or persistent epigastric discomfort, after 20 weeks of gestation.	MetS	Numbers and percentages.	N/a

Keepanasseril (2020)	India	2018 - 2019	Prospective cohort study	Women with PE admitted to the Women and Children's Hospital during the study period.	CH.	<u>PE/E</u><br>*E*<br>*HELLP*	ACOG 2013 criteria.	HT	Chi-squared test, Fisher's exact test. Multivariate logistic regression analysis.	Age, parity, highest BP in labor, eclampsia, gestation at birth, birthweight, antihypertensive at 7 days.
Fatma (2017)	India	Ex: NR<br>Ou: 2014 - 2016	Retrospective cohort study	Women with PE at the Department of Obstetrics and Gynecology from the Era's Lucknow Medical College and age-matched controls from the same hospital during the study period.	DM, HT or CKD diagnosed before pregnancy, malignancy, end stage diseases, chronic inflammatory diseases or autoimmune disorders.	PE	PE: DBP ≥ 90 mmHg and proteinuria > 300mg/24h, > 1000g/L or ≥2+ on dipstick test after 20 weeks of gestation.	HT, T2DM, DL, MetS	Chi-squared test.	N/a
Ernawati (2019)	Indonesia	2013 - 2014	Retrospective cohort study	Women with early- and late-onset severe PE who underwent pregnancy termination in Dr. Soetomo General Hospital during the hospital period.	CH, DM, obesity, congenital heart disease, rheumatism, autoimmune disease, living outside the city of Surabaya, death.	PE	Not defined.	HT, T2DM, DL, MetS	Chi-squared test, Fisher's exact test.	N/a
Watanabe (2020)	Japan	2018 - 2020	Case-control study	Hypertensive women recruited from three settings: (1) outpatients to the Department of Nephrology and HT at Dokkyo Medical University Hospital; (2) outpatients to the Department of Cardiovascular Medicine and Nephrology, Dokkyo Medical University Nikko Medical Center; and (3) residents who completed an annual health check-up in the town of Mibu (cases). Normotensive residents identified in the annual health check-up in the town of Mibu (controls).		<u>HDP</u><br>GH<br>PE	ACOG 2013 criteria. Early onset: < 34 week of gestation.	HT	T-test, Fishers exact test, univariate and multivariable logistic regression.	Age, BMI, age at first delivery.

Wagata (2020)	Japan	Ex: NR<br>Ou: 2013 - 2016	Cross-sectional population-based study	Parous women from the prospective Tohoku Medical Megabank CommCohort Study.	Age at delivery ≤9y, missing data on age at delivery, no data on BP.	HDP	Identified from questionnaires. Not defined.	HT	Chi-squared test, Fisher's exact test, multivariate logistic regression analysis.	Age, BMI, family history of HT, alcohol consumption.
Mito (2018)	Japan	Ex: 2003 - 2005<br>Ou: 2008-2010	Prospective cohort study	Women who received antenatal care at National Center for Child Health and Development and the Showa University Hospital Mother and Child Health Center from the first trimester during the study period and delivered singletons.	Women with a stillbirth, a miscarriage, CH, DM or kidney disease before pregnancy, SBP ≥ 140/90 <20 weeks of gestation, no documented BP <20 weeks' gestation, inability to confirm that the BP was less than 140/90 within 12 weeks after the index delivery, onset of HT during labor, pregnancy or breastfeeding during follow-up visit.	GH/PE	PE: BP ≥ 140/90 mmHg and proteinuria ≥ 300mg/24h or ≥2+ on dipstick test or ≥ 1+ on 2 separate occasions between 20 weeks of gestation and 12 weeks postpartum.<br>GH: BP ≥ 140/90 between 20 weeks of gestation and 12 weeks postpartum without proteinuria.	HT, T2DM, DL, CKD, MetS	Chi-squared test, Fisher's exact test, logistic regression analysis.	Age at delivery, BMI, family history of HT and salt intake.
Oishi (2017)	Japan	Ex: NR<br>Ou: 2011 - Juni 2015	Population-based cohort study	Women who participated in het Iwaki Health Promotion Project, aged >35y, with complete antenatal records of their first singleton pregnancy, with at least five BP measurements during the pregnancy.		GH<br>PE<br>sPE	HDP: BP ≥ 140/90 mmHg with or without proteinuria between 20 weeks of gestation and 12 weeks postpartum.	HT, T2DM, DL, CKD	Chi-squared test, Fisher's exact test, multivariate logistic regression analysis.	Current age, BMI, age at delivery, HT, DL, DM.
Watanabe (2015)	Japan	Ex: NR<br>Ou: 2012 - 2013	Retrospective cohort study	Parous women who underwent health checkup at a periodic health examination facility in Aichi, Japan, during the study period.	HT (BP ≥ 140/90) or proteinuria <20 weeks of pregnancy or >12 weeks after delivery.	GH/PE	Identified from maternal and child health handbooks and questionnaires.<br>BP ≥ 140/90 mmHg with or without proteinuria (≥ 300mg/24h) between 20 weeks of gestation and 12 weeks postpartum.	HT, T2DM, DL	Fisher's exact test, multivariate logistic regression analysis.	Age, BMI, menopauzal status.
Kurabayashi (2013)	Japan	2001-2007	Cross-sectional study	Parous women from the Japan Nurses' Health Study, aged ≥45y at baseline.	BP ≥ 140/90 mmHg or proteinuria either <20th gestational week or >12 weeks after delivery.	GH/PE	Identified from questionnaires.<br>GH/PE: BP ≥ 140/90 mmHg with and without proteinuria.	HT, T2DM	Multivariable logistic regression analysis.	Age at survey, current BMI, smoking, alcohol consumption.

Nohira (2013)	Japan	E: 1998-2001<br>Ou: NR	Prospective cohort study	Women with severe PE (cases) and normotensive women (controls), matched for age, parity, pre-pregnancy BMI, smoking habits and family history of DM, CVD and PE, who delivered at Hachioji Medical Center of Tokyo Medical University during the study period.	CVD before pregnancy.	Sev PE	Severe PE: BP ≥ 160/110 mmHg and proteinuria ≥2+ on dipstick test.	HT	Chi-squared test.	N/a
Cho (2019-1)	Republic of Korea	E: 2004<br>Ou: 2005-2012	Retrospective cohort study	Primigravid women with PE who delivered in 2004 and who underwent a national health screening examination within 1-2 years before their first delivery.	CH.	PE	ICD-10.	HT	Cumulative incidence.	N/a
Cho (2019-2)	Republic of Korea	E: 2011 - 2012	Prospective cohort study	Primigravid women who delivered between Jan 2011 and Dec 2012, who underwent a national health screening examination 1-2 years before delivery and withing 2 years after delivery.	CH.	HDP*	ICD-10.	HT, T2DM, DL, MetS	Chi-squared test, multivariate logistic regression analysis.	Age, pre-pregnancy BMI, smoking status, insurance premium, time before and time after pregnancy.
Park (2018)	Republic of Korea	Ex: 2010 - 2012<br>Ou: through 2013	Prospective cohort study	Women with a delivery in the study period.	CVD before pregnancy, missing data on one or more components of the Charlson comorbidity index before pregnancy.	PE	ICD-10.	CVD	Chi-squared test, multivariate logistic regression analysis.	NR
Yang (2015)	Republic of Korea	Ex: NR<br>Ou: 2004-2008	Prospective cohort study	Parous women from the HEXA cohort.	Pre-pregnancy disorders, missing data on PE history, age at first pregnancy, past/current metabolic disorders and anthropometry measures.	PE	Identified from interviews. Not defined.	HT, T2DM, DL, MetS	Hierarchical multivariate logistic regression analysis. Stratified analysis by age.	Age at enrollment, age at first pregnancy, number of childbirths, BMI before pregnancy, history of spontaneous abortion, history of artificial abortion, breastfeeding experience, use of OCP, menopausal status, education level, smoking

										status, drinking status, physical exercise.
Li (2018)	Singaporean *56% White* *15% Black* *28% Brown*	2009 - 2010	Prospective cohort study	Women from the population-based GUSTO birth cohort with GDM and matched women without GDM, who attended both baseline and 5y follow-up visits.	CH or DM at baseline, women who were pregnant at the 5y follow-up visit.	GH/PE	Not defined.	HT, MetS	Poisson regression analysis.	Age at enrolment, maternal ethnicity, maternal college education, pre-pregnancy BMI, cumulative parity.
Hung (2022)	Taiwanese	Ex: 2000-2017 Ou: 2000-2017	Population-based cohort study	Women, aged 18-45y, obtained from NHIRD with a pregnancy complicated by HDP between 2000 and 2017 and parous women without a history of HDP, matched by age, delivery type, gravidity, hospital level, delivery season, living area, family income and comorbidities (CH, GDM, anemia, APH, PPH) in a 4:1 ratio.	History of stroke.	HDP	ICD-9-CM/ICD-10-CM.	CVD	Cox proportional hazards regression analysis.	Type of delivery, multiple gestation, multiple HDP, hospital level, season during delivery, comorbidities (CH, GDM, anemia, delivery conditions incl APH and PPH), sociodemographic variables.
Wu (2021)	Taiwanese	Ex: 2006 - 2013 Ou: through 2015	Population-based cohort study	Women, aged 20-40y, obtained from NHIRD with a pregnancy complicated by HDP between Jan 2006 and Dec 2013 and parous women without a history of HDP, matched for age and date of delivery in 1:4 ratio.	Key information missing, history of CKD, CH or CAD before diagnosis of HDP, use of aspirin or LMWH for >30d in the 3 months after HDP.	GH/PE	ICD-9-CM.	CKD, CVD	Cox proportional hazards regression analysis.	Cancer, DM, HL, obesity, PCOS, systemic autoimmune rheumatic diseases, Charlson comorbidity index, geographic location.
Huang (2020)	Taiwanese	2000 - 2013	Case-control study	Women with HDP and without HDP, matched 1:3 by index day and age.	History of stroke prior to tracking, aged <12y, HDP before index date, peripartum and postpartum (<3months) stroke.	HDP	ICD-9.	CVD	Cox proportional hazards regression analysis.	Age group, area of residence, urbanization level of residence, annual income, insured premium, DM, HT, HL, obesity, heart disease, CKD, season, level of care.
Kuo (2018)	Taiwanese	1996 - 2010	Retrospective cohort study	Random selection of one million women from NHIRD with data entry at least 12 months before	Previous history of MI, congestive HF, peripheral vascular disease, cerebrovascular disease,	PE E	ICD-9-CM.	HT, T2DM, DL, CVD	Chi-squared test, Fisher's exact tests, Cox proportional	Age, DM, DL.

				pregnancy, of whom were selected women with PE and parous women without a history of PE, matched for age and date of delivery, in a 1:4 ratio.	DM, DL and HT before pregnancy.				hazards regression analysis.	
Hwu (2016)	Taiwanese	Ex: 1996-2011 (cases) / 2000-2011 (controls) Ou: through 2011	Cohort study	Women with a pregnancy complicated by HDP, aged 15-45y, identified from NHIRD (cases; divided in HDP/non-GDM and HDP/GDM). Additionally, pregnant women from a random selection of one million patients from NHIRD, aged 15-45y, without HDP and without GDM (controls), matched by age- and year of pregnancy in a 1:4 ratio with the cases.	Diagnosis of HDP before 2000, history of HT, DM or HDP and GDM.	GH<br>PE	ICD-9-CM.	HT, T2DM, CKD, CVD	Chi-squared test, Fisher's exact test, analysis of variance.	Renal disease, urbanization level.
Yeh (2014)	Taiwanese	E: 1998-2009 Ou: through 2009	Population-based cohort study	Random selection of one million women from NHIRD enrolled between Jan 1997 and Dec 2009, of whom were selected women with at least one pregnancy complicated by HDP between Jan 1998 and Dec 2009, and parous women without a history of HDP, matched for age and date of delivery in 1:4 ratio.	History of any CVD requiring hospitalization or HT in the 12 months before delivery.	GH<br>PE<br>E	ICD-9-CM.	HT, T2DM, DL, CVD	Chi-squared test, Cox proportional hazards regression analysis. Stratified analysis by time of onset of DHP and types of HDP. Logistic regression to estimate the synergy index of HDP and incident HT.	DM and DL at baseline.
Wu (2014)	Taiwanese	Ex: 1998-2002 Ou: through 2008	Population-based cohort study	Random selection of one million women from NHIRD registered from 1996 to 2008, of whom were selected women with a pregnancy between 1998 and 2002.	Multiple HDP, other possible causes of ESRD (DM, TMA, HUS, SLE, glomerulopathy, other nephritis or nephropathy and HT secondary to renal disease that complicated	HDP<br>GH<br>CH<br>PE/E<br>sPE	ICD-9-CM.	CKD	Chi-squared test, Poisson regression analysis, Cox proportional hazards	Age, delivery type, number of deliveries, complications from delivery.

					pregnancy, childbirth and the puerperium).				regression analysis.	
Wang (2013)	Taiwanese	Ex: 1998-2009<br>Ou: through 2009	Prospective cohort study	Random selection of one million women from NHIRD registered from 1996 to 2009, of whom were selected primigravid women, aged 19-40y, with a pregnancy complicated by HDP between 1998 and 2009 (cases), and primigravid women without a history of HDP (controls), matched for age and index year, in 1:8 ratio.	History of HT, DM, renal disease or SLE before the index data, women in the control group who developed HDP in a subsequent pregnancy before the development of ESKD.	GH<br>PE	ICD-9-CM.	CKD	Chi-squared test, Cox proportional hazards regression analysis.	Urban status, CAD, congestive HF, HL, placental abruption.
Wang (2012)	Taiwanese	Ex: 1997-2003<br>Ou: through 2008		Random selection of one million women from NHIRD registered from 1997 to 2003, of whom were selected primigravid women, aged 19-40y, with a pregnancy complicated by HDP (cases) between 1997 and 2003, and primigravid women without a history of HDP (controls), matched for age and index year, in 1:4 ratio.	GDM, DM or HT before the date of HDP diagnosis.	GH<br>PE	ICD-9.	T2DM	Incidence rate, Cox proportional hazards regression analysis, logistic regression analysis, simple linear regression analysis.	Age, occupation, obesity and HL.
Lin (2011)	Taiwanese	Ex: 1999-2003<br>Ou: 1996-2004	Population-based cohort study	Women who delivered between 1999 and 2003 that could be successfully linked to National Health Insurance hospital discharge data.	Maternal age ≤15y or ≥50y, infant birthweight ≤600 or ≥6000g, gestational week ≤19 or ≥45, parity ≥11, history of MACE before pregnancy.	(s)PE/E	ICD-9-CM.	CVD	Cox proportional hazards regression analysis.	Age, education, marital status, parity, infant sex, birthweight, gestational week, pregnancy and obstetric complications (multiple gestations, DM, long-term HT, pregnancy related HT, anemia, APH, PPH, CS, SLE)
Wang (2011)	Taiwanese	Ex: 2000-2004<br>Ou: through 2008	Population-based cohort study	Random selection of one million women from NHIRD registered from 1996 to 2008, of whom were selected primigravid	History of stroke or HT before pregnancy, missing information for age or sex.	GH/PE	ICD-9-CM.	T2DM, DL, CVD	Chi-squared test, Cox proportional hazards	Age, urbanization level.

				women with a pregnancy complicated by HDP between Jan 2000 and Dec 2004 (cases), and primigravid women without a history of GDP, frequency-matched in the same year (controls), in 1:4 ratio.					regression analysis.	
Soonthornpun (2009)	Thai	NR	Retrospective cohort study	Women with a history of severe PE at least 6 months prior to inclusion and age- and BMI-matched women with a history of a normal pregnancy at least 6 months prior to inclusion, in a ratio of 1:2.		Sev PE	Severe PE: BP ≥ 160/10 mmHg and proteinuria ≥ 500mg/24h or ≥2+ on dipstick test.	T2DM	Numbers and percentages.	N/a
**EUROPE**										
Neuman (2021)	Netherlands / Black	2012-2016	Prospective cohort study	Women with suspected or confirmed PE admitted to obstetrics department of the Erasmus Medical Center, with confirmed PE diagnoses within 1 week of study entry, and who attended a follow-up appointment within 9-15 months postpartum.	CH and/or proteinuria, chromosomal/fetal anomalies.	PE	ISSHP 2018 classification.	HT	Numbers and percentages.	N/a
**MIDDLE AND SOUTH AMERICA**										
Facca (2018)	Brazil<br>*56% White*<br>*15% Black*<br>*28% Brown*	Ex: 1976-2016<br>Ou: NR	Retrospective cohort study	Women with and without a history of HDP who presented voluntarily to the department.	Women with confounding factors prior to pregnancy, such as history of DM, systemic HT, nephropathy, kidney transplant, cardiopathy or collagenosis, multiple pregnancies or women currently suffering from urinary infection.	GH<br>PE	ISSHP 2018 classification.	HT, MetS	Chi-squared test.	N/a

Henriques (2014)	Brazil	Ex: 1992 - 2002<br>Ou: NR	Retrospective cohort study	Women with a HDP and a similar number of women without PE who gave birth at the Assis Chateaubriand Maternity Teaching Hospital during the study period.		Mild PE<br>Sev PE<br>CH<br>GH<br>sPE<br>E	Not defined. Identified from medical records.	HT, T2DM, DL	Chi-squared test.	N/a
Da Silva (2014)	Brazil	Ex: NR<br>Ou: 2011 - 2011		Women selected from the database of the women's health research group of the Federal University of Rio Grande do Norte with a history of PE and normotensive women who had their deliveries at the University Maternity Ward, five years prior to the initiation of the research.	Mental deficit, not being a resident of the city of Nadal, history of CVD, cognitive or neurological sequelae before pregnancy, incomplete information regarding PE diagnosis in medical records.	PE	Not defined. Identified from medical records.	HT, CVD	Chi-squared test.	N/a
Tornes (2020)	Cuba	2017 - 2020	Prospective cohort study	Women with PE admitted to HT unit of Obstetrics and Gynaecology department of Carlos Manuel de Cèspedes Teaching Hospital during the study period.	Pregnancy duration <24 weeks, CH, sPE, kidney disease.	PE	ACOG 2016 criteria.	HT	Binary and multivariate logistic regression analysis.	Age, doppler measurements of arteria uterina, serum PGT at baseline, serum creatinine at baseline, multiparity, BMI>30.
**NORTH AFRICA AND MIDDLE EAST**										
Behboudi-Gandevani (2020)	Iran	Ex: <1999<br>Ou: 1999-2014	Population-based cohort study	Women, aged 20-50y, with at least one pregnancy at the start of the study, from the Tehran Lipid and Glucose Study, a prospective population-based cohort study among inhabitants of Tehran.	CKD or HT at baseline (1999-2001), <1 follow-up visit.	PE	Identified using questionnaires.<br>PE: BP ≥ 140/90 mmHg and proteinuria ≥ 300mg/24h after 20 weeks of gestation.	CKD	Chi-squared test, multivariate logistic regression analysis, Cox proportional hazards regression analysis.	Age, BMI, SBP, DPB, smoking.
Amiri (2019)	Iran	Ex: <1999<br>Ou: 1999-2014	Population-based cohort study	Women, aged 20-50y, with at least one pregnancy at the start of the study, from the Tehran Lipid and Glucose Study, a prospective population-	HT at baseline, no follow-up visits.	PE	Identified by interviews or hospital records. ISSHP 2018 classification.	HT	Chi-squared test, pooled logistic regression analysis, multivariable	Age, BMI, TG, HDL, parity.

				based cohort study among inhabitants of Tehran.					time-dependent cox proportional hazard regression analysis.	
Hashemi (2013)	Iran	Births: <1998<br>Ou: 1998 - 2011	Population-based cohort study	Women, aged >15y, with history of HDP and age- and BMI-matched women with no history of HDP and at least one term pregnancy, from the Tehran Lipid and Glucose Study, a prospective population-based cohort study among inhabitants of Tehran.	CH at baseline.	GH/PE	Obstetric history obtained through interviews and from medical documents.<br>GH/PE: BP > 140/90 mmHg with or without proteinuria >300mg/24h after 20 weeks of gestation.	HT, T2DM, DL	Chi-squared test, ANCOVA general linear regression analysis.	T2DM, DL and HT at follow-up.
Borna (2012)	Iran	2009-2010	Case-control study	Women aged 35-65y with a high probability of CAD who underwent coronary angiography in the Tehran Heart Center during the study period. Those with positive findings (>50% stenosis in one of the three main coronary arteries) were identified as cases; those without positive findings were controls.	History of delivering a baby with any kind of abnormalities or malformations, history of severe PE or HELLP syndrome.	Mild PE	PE: BP ≥ 140/90 mmHg and proteinuria ≥ 300mg/24h or ≥1+ on dipstick test after 20 weeks of gestation<br>LBW: birthweight <2500g	CVD	Chi-squared test, Fisher's exact test, multivariate regression analysis.	Cigarette smoking, HT, HL, DM, age, BMI, WC.
Shahbazian (2011)	Iran	Ex: 2001 - 2003<br>Ou: 2008		Women with PE (cases) and normotensive women (controls), matched for year of delivery and age in a 1:1 ratio, who delivered at Razi Hospital and Emam Khomeini Hospital during the study period.		PE	PE: BP ≥ 140/90 mmHg and proteinuria ≥ 300mg/24h after 20 weeks of gestation	HT	Numbers and percentages.	N/a
Shammas (2000)	Jordan	Ex: 1988<br>Ou: 1999	Retrospective cohort study	Women with HDP and normotensive patients who delivered at the King Hussein Medical Centre during the study period.		GH<br>PE	Not defined.	HT, CVD	Percentages and numbers. Statistical test not described.	N/a

Qasim (2016)	Pakistan	Ex: NR<br>Ou: 2012 - 2013	Case-control study	Parous women that visited the outpatient medicine clinics of the Aga Khan University Hospital during the study period and were diagnosed with HT or pre-HT (cases) and those without HT (controls).	HT secondary to other disorders such as renal disease, hypo or hyperthyroidism, unmarried patients.	HDP	Obstetric history identified using questionnaire. HDP not defined.	HT	Univariate and multiple logistic regression analysis.	Age, family history of HT, physical activity, BMI.
Fadalallah (2016)	Sudan	2014 - 2014	Prospective cohort study	Women with PE that presented at Wad Medani tertiary hospital during the study period.	Thyroid disease, HT, renal disease, DM, liver disease.	Sev PE<br>Mild PE	PE: BP ≥ 140/90 mmHg and proteinuria ≥ 300mg/24h or ≥2+ on dipstick test after 20 weeks of gestation.<br>Mild PE: DBP <110 mmHg.<br>Severe PE: DBP ≥ 110 mmHg.	HT	Chi-squared test, multivariate logistic regression analysis.	Age, parity, duration of pregnancy, BMI, Hb, RBC, WBC, PC, AST, ALT, uric acid, neonatal weight, previous spontaneous delivery, severe PE, cesarean delivery, sex of neonate.
Aykas (2015)	Turkey	Ex: <2008<br>Ou: 2013	Retrospective cohort study	Women with PE who presented at the Department of Obstetrics and Gynecology of Kayseri Education and Research Hospital during the study period. Controls were randomly selected among women who had uncomplicated pregnancies during the same time period.		PE	PE: BP ≥ 140/90 mmHg and proteinuria ≥ 300mg/24h after 20 weeks of gestation.	HT, T2DM	Chi-squared test, Fisher's exact test.	N/a
**NORTH AMERICA**										
Malek (2021-1)	US, South Carolina / NHB	Ex: 2004-2016<br>Ou: 2004-2017	Retrospective cohort study	Women identified from statewide hospital discharge records and emergency department visit data, who delivered a singleton liveborn infant in a hospital in South Carolina.	History of maternal kidney transplantation, residence outside of SC, age <12y or >49y, pre-pregnancy weight <92lbs or >320lbs, pre-pregnancy BMI <16.0 or >52.8kg/m2, newborn weight <500 or >6000g, implausible size for GA at delivery, 'other' race/ethnicity, maternal prior diagnosis of HF, maternal congenital heart defect.	GH/PE<br>CH<br>sPE	ICD-9-CM/ICD-10-CM.	CVD	Cox proportional hazards regression analysis.	Sociodemographic (maternal age, education, rural/urban residence, median income, payer, Women, Infants and Children), behavioral (smoking during pregnancy), and clinical characteristics (pre-pregnancy BMI, change in BMI after delivery, DM or GDM, GA at delivery, mode of delivery, induced labor, number of pregnancies

										prior to index pregnancy, previous CS, previous PTB, Revised-Graduated Prenatal Care Utilization Index).
Malek (2021-2)	US, South Carolina / NHB, Hispanic	Ex: 2004-2016<br>Ou: 2004-2017	Retrospective cohort study	Women identified from statewide hospital discharge records and emergency department visit data, who delivered a singleton liveborn infant in a hospital in South Carolina.	History of maternal kidney transplantation, residence outside of SC, age <12y or >49y, pre-pregnancy weight <92lbs or >320lbs, pre-pregnancy BMI <16.0 or >52.8kg/m2, newborn weight <500 or >6000g, implausible size for GA at delivery, 'other' race/ethnicity.	GH/PE<br>CH<br>sPE	ICD-9-CM/ICD-10-CM.	CVD	Cox proportional hazards regression analysis, stratified by race/ethnicity. Interaction of race/ethnicity tested.	Sociodemographic (maternal age, education, rural/urban residence, median income, payer, women, infants and children), behavioral (pre-pregnancy smoking) and clinical characteristics (pre-pregnancy BMI, T2DM or GDM).
Lederer (2020)	US, Florida / NHB, Black Hispanic, White Hispanic	Ex: 2012 - 2013<br>Ou: 2014 - 2016	Retrospective cohort study	Women diagnosed with PE at Jackson Memorial Hospital during the study period.	Previous diagnosis of stroke, <18y.	PE	ACOG criteria/ICD-9.	HT	Numbers and percentages.	N/a
Wichmann (2019)	US, South Carolina / Black	Ex: NR<br>Ou: 2005 - 2014	Case-control study	Black women who had undergone CCTA between June 2005 and May 2014 in hospital within the Medical University of South Caroline health network, with a pregnancy complicated by either PTB, PE, GDM or with an uncomplicated pregnancy (1:1 ratio complicated vs uncomplicated).	History of multiple or repeated pregnancy complications, missing data about pregnancy complications and baseline characteristics, abortion or miscarriage, nonspontaneous PTB including induced PTB to treat PE, history of any myocardial or coronary disease before pregnancy, occurrence of a MACE before CCTA, any cardiac intervention including coronary catheter angiography before CCTA, CCTA examinations which were deemed nondiagnostic because of either severe motion or beam-hardening artifacts, or poor contrast	PE	Not defined.	CVD	Chi-squared test, univariate and multivariate logistic regression analysis.	BMI, smoking status, T2DM, HT, HL, age at first birth, age when undergoing the CCTA examination, number of pregnancies (any CAD), age at first birth (any CAD).

					opacification of the coronary arteries.					
Cirillo (2015)	US, California / African American	Ex: 1959-1967<br>Ou: through 2011	Prospective cohort study	Women receiving prenatal care from the Kaiser Health Plan at its clinics in Alamada County during the study period.	Missing parity or termination in multiple births, abortion, fetal death <20 weeks' gestation, maternal heart disease at baseline.	GH	ISSHP 2018 classification.	CVD	Cox proportional hazards regression analysis. All pregnancy complications were tested for interaction with race (African American versus all other), and race specific associations were examined in models stratified by race.	Age, race, parity, BMI, cigarette smoking status at observed pregnancy.
**SUB-SAHARAN AFRICA**										
Nganou-Gnindjio (2021)	Cameroon	2011 - 2016	Retrospective cohort study	Women, 18-45y, diagnosed with PE/E during pregnancy or postpartum at the Yaoundé Central Hospital and Yaoundé Gynaeco-Obstetric and Paediatric Hospital during the study period.<br>We included all women aged 18 to 45 years during five years (from December 2011 to December 2016) in the selected hospitals.	No available contact information, incomplete records, <6 months postpartum, pregnant at follow-up.	PE/E	Not defined.	HT	Numbers and percentages.	N/a
Taa Nguimbis Esseme (2019)	Cameroon	2012-2017	Case-control study	Women diagnosed with arteriosclerotic CVD at the Yaoundé General Hospital and Yaoundé Gynaeco-Obstetric and Paediatric Hospital (cases) and mothers of children who sought pediatric care	Incomplete administrative data, not residing in Yaoundé during the study period, <18y, >60y, DM, CVD before first pregnancy, <6months pregnant before 5th Oct	<u>HDP</u><br>GH<br>PE	Identified from interviews. GH: high BP without proteinuria, seizures or loss of consciousness. PE: high BP with proteinuria, seizures or loss of consciousness.	CVD	Crude and adjusted logistic regression analysis.	Smoking status, age, multiple gestation, family history of CVD, total number of pregnancies, marital status, level of education.

				at the Yaoundé Gynaeco-Obstetric and Paediatric Hospital (controls) during the study period.	2017, not speaking French or English.					
Amougou (2019)	Cameroon	2009 - 2016	Retrospective cohort study	Women with PE that presented at Yaoundé University Teaching Hospital during the study period.	Follow-up period <1y, sPE.	PE	Not defined.	HT	Chi-squared test, multivariable logistic regression analysis.	Age, profession, HT in siblings, GA at onset of PE, gravidity, obesity.
Kaze (2014)	Cameroon	2010 - 2012	Prospective cohort study	Women with severe PE and eclampsia, who attended at least three antenatal visits and subsequently delivered at the central maternity unit of the Yaoundé Central Hospital during the study period, and attend the follow-up visit 6 months after delivery.	History of HT, DM, kidney disease, HIV infection, viral hepatitis B or C, toxoplasmosis, syphilis, rubella, CMV, HSV, coxsackie virus, VZV and parvovirus B19.	Sev PE<br>E	Severe PE: BP ≥ 160/110 mmHg and proteinuria ≥ 5000mg/24h after 20 weeks of gestation<br>Eclampsia: onset of seizures in a patient with PE.	HT, CKD	Numbers and percentages.	
Osoti (2020)	Kenya	2016 - 2018	Prospective cohort study	Postpartum women, aged ≥28y, with or without GH or PE, who were stable and ready for discharge after delivery at Kenyatta National Hospital during the study period.	HIV infection, intention to become pregnant <3 years, planning to live >50 km from the hospital in the next 6 months, conditions suggestive of pre-pregnancy MetS such as pregestational DM, CH, malignancy, renal, hepatic or biliary diseases, use of statins, insulin, oral hypoglycemic agents, or antihypertensives before pregnancy, delivery <28 weeks, missing medical record, GDM.	GH/PE	ISSHP 2018 classification.	HT, T2DM, DL, MetS	Generalized linear model regression analysis with a Poisson distribution link.	Maternal age, level of education, hormonal contraception, breastfeeding, BMI, marital status, employment status.
Ishaku (2021-1, 2021-2, 2021-3) †	Nigeria	Ex: 2017 - 2018<br>Ou: through 2019	Prospective cohort study	Women who delivered in one of the eight participating tertiary hospitals in Nigeria during the study period.	Aged <18y, multiple pregnancies, medical disorders in pregnancy other than HDP (e.g. GDM, sickle cell disease, heart disease, kidney or	GH<br>CH<br>PE<br>E	ISSHP 2018 classification.	HT, CKD, MetS	Numbers and percentages.	N/a

					other connective tissue disease).					
Olagbuji (2012)	Nigeria	2009 - 2010	Prospective cohort study	Women who were managed for new-onset HDP, who had antenatal, intrapartum and postpartum follow-up care, at the Department of Obstetrics and Gynaecology, University of Benin Teaching Hospital.	CH, unclassified HT or HT developed during the puerperium.	GH/PE	ISSHP 2007 classification.	HT	Numbers and percentages.	N/a
Ntlemo (2021)	South Africa<br>*87% Black African*<br>*8% Coloured*<br>*2% White*<br>*2% Indian*	2019 - 2020	Prospective cohort study	Women with PE identified at daily morning ward rounds in two tertiary hospitals, and normotensive controls identified at a district-level hospital, during the study period.		PE	ISSHP 2018 classification.	HT, MetS	Numbers and percentages.	N/a
Mooij (2021)	Tanzania	Ex: 2011 - 2012<br>Ou: 2018 - 2018	Prospective cohort study	Women with severe PE or eclampsia and women with normotensive pregnancies who were treated in Ndala Hospital during the study period.	Aged <18 years, severe mental disability, participation constituting more than a minor burden, delivery <6 weeks before the interviews were conducted. Among the cases: severe PE or eclampsia in a subsequent pregnancy. Among the controls: HDP in a previous pregnancy.	Sev PE<br>E	Not defined.	HT	Numbers and percentages.	N/a
Nakimuli (2013)	Uganda	2009 - 2011	Prospective cohort study	Women admitted with PE or eclampsia at Mulago Hospital labour ward and postnatal clinics during the study period, who attended the 3 months postnatal visit.	CH before and during first 20 weeks of pregnancy, renal disease before or during pregnancy, residing outside of the region.	PE	PE: BP ≥ 140/90 mmHg and proteinuria ≥2+ on dipstick test after 20 weeks of gestation<br>Mild PE: BP < 160/110 mmHg and proteinuria ≤ 2+<br>Severe PE: BP ≥ 160/110 and proteinuria ≥ 3+<br>Eclampsia: PE with generalized tonic-clonic convulsions	HT	Chi-squared test, multivariable logistic regression analysis.	Age, parity, KIR genotype, GA.

Ndayambagye (2010)	Uganda	2008 - 2009	Prospective cohort study	Women admitted with PE or eclampsia at Mulago Hospital labour ward and postnatal clinics during the study period, who were followed up to the end of puerperium.	Essential HT, CH, HT or PE in a prior pregnancy, a history of HT in the first trimester, renal disease.	Mild PE<br>Sev PE<br>E<br>HELLP	PE: BP ≥ 140/90 mmHg or rise in SBP of ≥ 30 mmHg and DBP of ≥ 15 mmHg and proteinuria ≥ 1+ on dipstick test after 20 weeks of gestation<br>Mild PE: BP < 160/110 mmHg and proteinuria ≤ 2+<br>Severe PE: BP ≥ 160/110 and proteinuria ≥ 3+<br>Eclampsia: PE with convulsions (tonic-clonic seizures)<br>HELLP syndrome: BP ≥ 140/90 mmHg or rise in SBP of ≥ 30 mmHg and DBP of ≥ 15 mmHg, elevated HDL (> 600 IU/L), elevated AST/ALT (>50 IU/L) and platelet count < 150,000 cells/mm3.	HT	Numbers and percentages.	N/a

Abbreviations: 2hPG: 2 hour plasma glucose; ACOG: The American College of Obstetricians and Gynecologists; AI/AN: American Indian / Alaska Native; ALT: alanine aminotransferase; APH: antepartum hemorrhage; AST: aspartate aminotransferase; BMI: body mass index; BP: blood pressure; CAD: coronary artery disease; CCTA: coronary computed tomography angiography; CH: chronic hypertension; CKD: chronic kidney disease; CMV: cytomegalovirus; CS: cesarean section; CVD: cardiovascular disease; DBP: diastolic blood pressure; DL: dyslipidemia; DM: diabetes mellitus; E: eclampsia; ESKD: end-stage kidney disease; ESRD: end-stage renal disease; Ex: exposure; FPG: fasting plasma glucose; GA: gestational age; GDM: gestational diabetes mellitus; GH: gestational hypertension; Hb: hemoglobin; HDL: high-density lipoprotein; HDP: hypertensive disorders of pregnancy; HELLP: hemolysis, elevated liver enzymes and low platelets; HF: heart failure; HL: hyperlipidemia; HSV: herpes simplex virus; HT: hypertension; HUS: hemolytic uremia syndrome; ICD: International Classification of Diseases; ISSHP: International Society for the Study of Hypertension in Pregnancy; KIR: Killer Immunoglobulin-like receptors; LBW: low birthweight; LDH: lactate dehydrogenase; LMWH: low molecular weight heparin; MACE; major adverse cardiovascular event; MetS: metabolic syndrome; MI: myocardial infarction; n/a: not applicable; NHB: non-Hispanic black; NHIRD: National Health Insurance Research Database (Taiwan); NHW: non-Hispanic white; NR: not reported; OCP: oral contraceptive pill; OR: odds ratio; Ou: outcome; PC: platelet count; PCOS: polycystic ovary syndrome; PE: preeclampsia; PGT: pyruvate glutamic transaminase; PPH: postpartum hemorrhage; PTB: preterm birth; RBC: red blood cell count; RR: relative rate; SBP: systolic blood pressure; SC: South Caroline; sev: severe; SLE: systemic lupus erythematosus; sPE: preeclampsia superimposed on chronic hypertension; T2DM: type 2 diabetes mellitus; TG: triglycerides; TMA: thrombotic microangiopathy; UK: United Kingdom; US: United States of America; VZV: varicella zoster virus; WBC: white blood cell count; WC: waist circumference. * Identified by original authors as PE, redefined as HDP based on ICD-10-CM codes mentioned in the article. † Three separate articles by Ishaku et al, published in 2021, using the exact same population, reporting on three different outcome parameters.

Table S5: Prevalence / incidence and outcome of HDP in non-White populations.												
Author (year)	Country	Race, ethnicity or origin	Number	Socio-demographic	Prevalence / incidence of HDP (%)							Severity / complications of HDP*
					Any HDP	CH	GH	PE	E	sPE	Comparison (95% CI)	Maternal or neonatal
**ASIA**												
Loi (2007)	Singapore	Chinese	17099	NR				Sev: 0.3			p<0.001	
		Malay	9294	NR				Sev: 0.4			[ref]	
		Indian	2995	NR				Sev: 0.2			p=0.17	
		Others	2313	NR				Sev: 0.1			p=0.09	
Tan (2006)	Singapore	Chinese	1155	NR				3.5			p<0.001	
		Malay	760	NR				4.2			[ref]	
		Indian	164	NR				2.6			NR	
		Others	134	NR				3.0			NR	
**EUROPE**												
Bastola (2022)	Finland	Finland	350548	Obesity: 13%<br>Sm: 16%<br>Upper-level employee: 21%<br>P0: 30%	4.6		2.3	1.6			[ref]	
		Western high-income countries	2290	NR	2.7		0.8	1.6			HDP aRR: 0.60 (0.47–0.78)<br>GH aRR: 0.34, p<0.05<br>PE aRR: 1.00, NS	

	Eastern Europe	2566	Obesity: 10%<br>Sm: 9%<br>Upper-level employee: 34%<br>P0: 39%	2.3	0.9	1.1	HDP aRR: 0.54 (0.42–0.70)<br>GH aRR: 0.44, p<0.05<br>PE aRR: 0.69, NS
	Russia	11994	Obesity: 8%<br>Sm: 17%<br>Upper-level employee: 12%<br>P0: 33%	2.3	1.0	1.0	HDP aRR: 0.56 (0.50–0.64)<br>GH aRR: 0.45, p<0.05<br>PE aRR: 0.69 (0.58-0.83)
	South Asia	1904	Obesity: 8%<br>Sm: 17%<br>Upper-level employee: 10%<br>P0: 36%	2.7	1.1	1.5	HDP aRR: 0.63 (0.48–0.83)<br>GH aRR: 0.48, p<0.05<br>PE aRR: 0.91, NS
	East Asia	4948	Sm: 1%	1.3	0.3	0.9	HDP aRR: 0.33 (0.26–0.43)<br>GH aRR: 0.19, p<0.05<br>PE aRR: 0.61 (0.45-0.83)
	Sub-Saharan Africa	3548	Obesity: 8%<br>Upper-level employee: 16%<br>P0: 43%	4.2	1.1	3.0	HDP aRR: 0.84 (0.71–1.00)<br>GH aRR: 0.41 (0.30-0.56)<br>PE aRR: 1.77 (1.44-2.17)
	Middle East / North Africa	3465	Obesity: 2%<br>Sm: 4%<br>Upper-level employee: 15%<br>Nullipara: 41%	1.9	0.6	1.2	HDP aRR: 0.39 (0.30–0.50)<br>GH aRR: 0.23, p<0.05<br>PE aRR: 0.76, NS
	Latin America / Caribbean	739	Obesity: 21%<br>Sm: 2%<br>Upper-level employee: 4%<br>P0: 25%	2.2	0.8	1.2	HDP aRR: 0.52 (0.32–0.85)<br>GH aRR: 0.40, p<0.05<br>PE aRR: 0.79, NS

Siddiqui (2020)	France	European	4947	Language barrier: 3%<br>Social deprivation: 21%		Sev: 0.9	[ref]
		North Africa	2106	Language barrier: 16%<br>Social deprivation: 39%		Sev: 0.6	aOR: 0.80 (0.33-1.94)
		Sub-Saharan Africa	1558	Language barrier: 15%<br>Social deprivation: 60%		Sev: 1.6	aOR: 2.53 (1.39-4.58)
		Other	855	Language barrier: 36%<br>Social deprivation: 44%		Sev: 1.2	aOR: 1.34 (0.59-3.06)
Sole (2018)	Norway	Norway	724102	NR	P0: 2.4<br>P1+: 1.5	P0: 5.3<br>P1+: 2.4	[ref]
		Europe / EEA	54859	NR	P0: 2.0<br>P1+: 1.2	P0: 3.5<br>P1+: 1.6	GH - *P0* aOR: 0.78 (0.70-0.86)<br>- *P1+* aOR: 0.77 (0.68-0.86)<br>PE - *P0* aOR: 0.71 (0.66-0.77)<br>- *P1+* aOR: 0.68 (0.62-0.76)
		Europe / non EEA	21533	NR	P0: 1.3<br>P1+: 0.9	P0: 3.1<br>P1+: 1.9	GH - *P0* aOR: 0.57 (0.47-0.69)<br>- *P1+* aOR: 0.57 (0.47-0.71)<br>PE - *P0* aOR: 0.59 (0.52-0.67)<br>- *P1+* aOR: 0.78 (0.68-0.89)
		North America	3983	NR	P0: 1.7<br>P1+: 1.2	P0: 4.1<br>P1+: 1.1	GH - *P0* aOR: 0.64 (0.43-0.95)<br>- *P1+* aOR: 0.75 (0.51-1.11)<br>PE - *P0* aOR: 0.84 (0.65-1.09)<br>- *P1+* aOR: 0.44 (0.29-0.67)
		Latin America / Caribbean	7847	NR	P0: 1.2<br>P1+: 1.0	P0: 4.6<br>P1+: 2.0	GH - *P0* aOR: 0.45 (0.33-0.62)<br>- *P1+* aOR: 0.67 (0.48-0.92)<br>PE - *P0* aOR: 0.86 (0.73-1.02)<br>- *P1+* aOR: 0.78 (0.61-0.98)

		Middle East / North Africa	19552	NR		P0: 1.1<br>P1+: 0.8	P0: 2.7<br>P1+: 1.5	GH - *P0* aOR: 0.48 (0.38-0.62)<br>- *P1*+ aOR: 0.51 (0.41-0.64)<br>PE - *P0* aOR: 0.49 (0.41-0.57)<br>- *P1*+ aOR: 0.56 (0.48-0.66)	
		Sub-Saharan Africa	24029	NR		P0: NR<br>P1+: 0.8	P0: 5.7<br>P1+: 2.5	GH - *P0* aOR: 0.50 (0.39-0.63)<br>- *P1*+ aOR: 0.48 (0.39-0.59)<br>PE - *P0* aOR: 1.00 (0.90-1.12)<br>- *P1*+ aOR: 0.95 (0.85-1.07)	
		Transcaucasia / Central Asia	806	NR		P0: 1.4<br>P1+: 0.7	P0: 1.7<br>P1+: 1.6	GH - *P0* aOR: 0.50 (0.19-1.34)<br>- *P1*+ aOR: 0.48 (0.15-1.50)<br>PE - *P0* aOR: 0.26 (0.10-0.69)<br>- *P1*+ aOR: 0.73 (0.35-1.54)	
		South Asia	19388	NR		P0: 1.6<br>P1+: 1.1	P0: 3.4<br>P1+: 2.1	GH - *P0* aOR: 0.60 (0.48-0.75)<br>- *P1*+ aOR: 0.72 (0.60-0.87)<br>PE - *P0* aOR: 0.63 (0.54-0.73)<br>- *P1*+ aOR: 0.75 (0.65-0.86)	
		East Asia / Pacific	25457	NR		P0: 1.2<br>P1+: 0.9	P0: 3.5<br>P1+: 1.8	GH - *P0* aOR: 0.49 (0.41-0.58)<br>- *P1*+ aOR: 0.57 (0.47-0.69)<br>PE - *P0* aOR: 0.61 (0.55-0.68)<br>- *P1*+ aOR: 0.71 (0.62-0.81)	
		Oceania	535	NR		P0: 2.7<br>P1+: 1.1	P0: 3.1<br>P1+: 2.2	GH - *P0* aOR: 1.29 (0.60-2.74)<br>- *P1*+ aOR: 0.79 (0.25-2.48)<br>PE - *P0* aOR: 0.66 (0.31-1.42)<br>- *P1*+ aOR: 0.88 (0.36-2.13)	
Nilsen (2018)	Norway	Norwegian	1123762	P0: 41%<br>Income <p25: 25%<br>Primary school or below: 22%<br>Sm: 18%	0.5		3.5	[ref]	[PE <37w]<br>0.8%<br>[PE <34w]<br>0.3%
		Immigrant	163508				2.5	NR	
		Immigrant from Nordic country	22594	P0: 46%<br>Income <p25: 16%<br>Primary school or below: 11%<br>Sm: 12%			3.2	aOR: 0.87 (0.80-0.94)	

	Refugees	29422	P0: 32%<br>Income <p25: 37%<br>Primary school or below: 58%<br>Sm: 8%	2.7	aOR: 0.81 (0.75-0.88)	[PE <37w] 0.9%<br>[PE <34w] 0.5%
	Family immigants	89523	P0: 42%<br>Income <p25: 35%<br>Primary school or below: 39%<br>Sm: 6%	2.3	aOR: 0.62 (0.59-0.65	[PE <37w] 0.7%<br>[PE <34w] 0.3%
	Labour immigrants	13618	P0: 61%<br>Income <p25: 13%<br>Primary school or below: 8%<br>Sm: 9%	2.2	aOR: 0.55 (0.49-0.62)	[PE < 37w] 0.5%<br>[PE < 34w] 0.2%
	Immigrant students	8351	P0: 59%<br>Income <p25: 24%<br>Primary school or below: 13%<br>Sm: 3%	2.9	aOR: 0.75 (0.65-0.86)	[PE <37w] 0.8%<br>[PE <34w] 0.4%
	Vietnam	4937	NR	1.2	aOR: 0.32 (0.24-0.42)	
	China	2026	NR	1.4	aOR: 0.36 (0.25-0.53)	
	Morocco	2565	NR	1.6	aOR: 0.43 (0.31-0.60)	
	Iran	3015	NR	1.8	aOR: 0.44 (0.33-0.58)	
	Great Britain	1964	NR	1.7	aOR: 0.44 (0.31-0.63)	
	Lithuania	3462	NR	1.6	aOR: 0.44 (0.34-0.58)	
	Thailand	5830	NR	2.0	aOR: 0.51 (0.42-0.62)	

	Germany	3992	NR	1.9	aOR: 0.51 (0.40-0.64)
	Netherlands	1183	NR	1.9	aOR: 0.51 (0.32-0.82)
	United states	1946	NR	2.0	aOR: 0.53 (0.38-0.73)
	Ukraine	1138	NR	2.1	aOR: 0.55 (0.36-0.84)
	Afghanistan	2815	NR	1.9	aOR: 0.55 (0.40-0.75)
	Romania	1701	NR	2.2	aOR: 0.56 (0.40-0.77)
	F Yugoslavia	11040	NR	2.0	aOR: 0.56 (0.49-0.65)
	Latvia	983	NR	2.2	aOR: 0.60 (0.39-0.93)
	Turkey	4025	NR	2.3	aOR: 0.61 (0.49-0.76)
	Iraq	9052	NR	2.2	aOR: 0.61 (0.52-0.71)
	Sri Lanka	4963	NR	2.5	aOR: 0.62 (0.51-0.75)
	Denmark	5680	NR	2.2	aOR: 0.62 (0.52-0.75)
	Pakistan	7671	NR	2.3	aOR: 0.65 (0.55-0.77)
	Poland	9014	NR	2.6	aOR: 0.69 (0.60-0.79)
	Russia	5464	NR	2.5	aOR: 0.70 (0.59-0.83)
	Estonia	737	NR	2.8	aOR: 0.75 (0.46-1.24)
	Eritrea	2177	NR	2.8	aOR: 0.76 (0.58-1.01)
	Brazil	1628	NR	3.1	aOR: 0.78 (0.58-1.05)
	India	2249	NR	2.9	aOR: 0.79 (0.61-1.02)
	Finland	2575	NR	3.1	aOR: 0.82 (0.64-1.05)
	Myanmar	628	NR	3.2	aOR: 0.90 (0.55-1.45)
	Chile	816	NR	3.6	aOR: 0.93 (0.63-1.38)

		Sweden	12726	NR		3.6	aOR: 0.96 (0.86-1.06)	
		Ethiopia	1970	NR		3.7	aOR: 1.02 (0.79-1.31)	
		Somalia	12429	NR		3.2	aOR: 1.05 (0.93-1.20)	
		Philippines	5986	NR		4.2	aOR: 1.06 (0.93-1.22)	
		Ghana	906	NR		4.3	aOR: 1.13 (0.79-1.62)	
		Iceland	1677	NR		3.6	aOR: 1.13 (0.85-1.50)	
		Kenya	498	NR		4.2	aOR: 1.15 (0.73-1.82)	
		Nigeria	465	NR		4.3	aOR: 1.20 (0.79-1.84)	
		Burundi	355	NR		5.9	aOR: 1.75 (1.05-2.91)	
		Congo	538	NR		5.9	aOR: 1.93 (1.28-2.90)	
		Bangladesh	269	NR		7.8	aOR: 2.15 (1.32-3.50)	
		Tanzania	298	NR		7.4	aOR 2.21 (1.43-3.41)	
Panaitescu (2017)	UK	Caucasian	82797	NR	0.9		[ref]	
		African Caribbean	17888	NR	3.3		OR: 3.03 (2.71-3.39)	
		South Asia	4535	NR	1.2		OR: 1.93 (1.46-2.54)	
		East Asia	2094	NR	0.7		NR	
		Mixed	2618	NR	0.7		NR	
Naimy (2015)	Norway	Norway	1062078	Age ≥ 35y: 12%<br>P0: 42%		3.7	[ref]	[PTB]<br>23.9%, [ref]
		Pakistan	11297	Age ≥ 35y: 10%<br>P0: 30%		2.8	aOR: 0.83 (0.74-0.93)	[PTB]<br>34.1%, p<0.001

		Vietnam	6161	Age ≥ 35y:<br>13%<br>P0: 38%		1.4	aOR: 0.38 (0.31-0.47)	[PTB]<br>44.9%, p<0.001
		Somalia	5401	Age ≥ 35y:<br>14%<br>P0: 25%		4.0	aOR: 1.25 (1.09-1.43)	[PTB]<br>37.7%, p<0.001
		Sri Lanka	4918	Age ≥ 35y:<br>13%<br>P0: 44%		3.1	aOR: 0.77 (0.66-0.91)	[PTB]<br>42.9%, p<0.001
		Philippines	4651	Age ≥ 35y:<br>20%<br>P0: 46%		3.7	aOR: 0.93 (0.80-1.09)	[PTB]<br>32.1%, p<0.01
		Iraq	3818	Age ≥ 35y:<br>13%<br>P0: 36%		2.2	aOR: 0.57 (0.46-0.70)	[PTB]<br>39.5%, p<0.01
		Thailand	3202	Age ≥ 35y:<br>17%<br>P0: 40%		2.2	aOR: 0.55 (0.43-0.70)	[PTB]<br>26.5%, p=0.73
		Afghanistan	663	Age ≥ 35y:<br>10%<br>P0: 32%		2.0	aOR: 0.54 (0.31-0.94)	[PTB]<br>38.5%, p=0.36
		All migrants	NR	NR		2.7	aOR: 0.76 (0.71-0.81)<br>- < 5y of residence: aOR 0.64 (0.59-1.70)<br>- ≥ 5y of residence: aOR 0.91 (0.84-0.99)	[PTB]<br>35.5%, p<0.001
Bouthoorn (2012)	The Netherlands	Dutch	3886	BMI: 23.2<br>Low ed: 17%<br>Sm: 17%<br>P0: 59%	5.2	1.9	[ref]	
		Surinamese-Creoles	232	BMI: 24.6<br>Low ed: 36%<br>Sm: 20%<br>P0: 56%	5.5	2.4	GH aOR: 0.86 (0.45-1.56)<br>PE aOR: 1.13 (0.43-3.06)	

		Surinamese-Hindustani	250	BMI: 23.2<br>Low ed: 38%<br>Sm: 14%<br>P0: 57%	3.4	3.8		GH aOR: 0.63 (0.29-1.36)<br>PE aOR: 1.80 (0.84-3.87)
		Turkish	718	BMI: 24.7<br>Low ed: 52%<br>Sm: 30%<br>P0: 45%	1.7	1.6		GH aOR: 0.31 (0.16-0.59)<br>PE aOR: 0.75 (0.36-1.55)
		Moroccan	534	BMI: 25.1<br>Low ed: 54%<br>Sm: 6%<br>P0: 40%	1.5	0.8		GH aOR: 0.28 (0.13-0.61)<br>PE aOR: 0.38 (0.13-1.11)
		Cape Verdean	331	BMI: 23.6<br>Low ed: 50%<br>Sm: 23%<br>P0: 57%	3.2	4.2		GH aOR: 0.64 (0.32-1.29)<br>PE aOR: 2.06 (1.04-4.09)
		Antillean	264	BMI: 24.5<br>Low ed: 44%<br>Sm: 20%<br>P0: 58%	2.9	3.7		GH aOR: 0.47 (0.21-1.06)<br>PE aOR: 1.87 (0.86-4.06)
Zwart (2008)	The Netherlands	Dutch	280899	NR			0.1	[ref]
		Morocco / Turkey	29384	NR			0.1	NR
		Surinam / Dutch Antilles	13226	NR			0.1	RR 2.5 (1.3-4.9)
		Other non-Western	20577	NR			0.1	[Non-Western vs Dutch]<br>RR 1.8 (1.3-2.4)<br>[Sub-Saharan African vs Dutch]<br>RR 6.2 (3.6-10.6)
		Other Western	32821	NR			<0.1	NR

Lydakis (1998)	UK	White	64	BMI 28+-7<br>P0: 25%<br>Sm: 21%			[overall] p=0.58	[sPE among women with CH]<br>CH 17.2%<br>[perinatal mortality]<br>CH 1.6%<br>[PTB]<br>CH 25.0%<br>[SGA]<br>CH 35.9%
		Black / African Caribbean	79	BMI 28+6<br>P0: 11%<br>Sm: 8%				[sPE among women with CH]<br>CH 12.7%<br>[perinatal mortality]<br>CH 3.8%<br>[PTB]<br>CH 32.9%<br>[SGA]<br>CH 36.7%
		Indo Asian	70	BMI 27+5<br>P0: 13%<br>Sm: 0 %				[sPE among women with CH]<br>CH 18.6%<br>[perinatal mortality]<br>CH 10.0%<br>[stillbirth]‡<br>CH RR 9.4 (3.7-23.8)<br>[PTB]<br>CH 41.4%<br>[SGA]<br>CH 54.3%
**NORTH AFRICA AND THE MIDDLE EAST**								
Segal (1996)	Israel	Israeli	269	No ed:<br>1%<br>P5+: 2%		Mild: 7.1<br>Sev: 0.0	[ref]	
		Ethiopian immigrant	269	No ed:<br>79%<br>P5+: 9%		Mild: 11.9<br>Sev: 3.0	Mild: p<0.05<br>Sev: p<0.05	
**NORTH AMERICA**								
Boakye (2021) †	US	NHW<br>- US-born<br>- Foreign-born	997<br>- 776<br>- 221	NR	US-b:<br>3.0<br>F-b: 2.3	US-b: 7.1<br>F-b: 7.2	[ref]<br>[US-b vs F-b]<br>PE aOR: 0.98 (0.49-1.96)<br>- *Resid < 10y* aOR: 1.20 (0.48-3.02)	

							- *Resid* ≥ *10y* aOR: 1.18 (0.31-4.48)	
		Hispanic<br>- US-born<br>- Foreign-born	2400<br>- 556<br>- 1844	NR	US-b: 2.5<br>F-b: 2.8	US-b: 7.9<br>F-b: 9.1	[Hispanic vs NHW]<br>PE aOR: 1.16 (0.84-1.59)<br>[US-b vs F-b]<br>PE aOR: 1.07 (0.72-1.60)<br>- *Resid < 10y* aOR: 1.04 (0.67-1.59)<br>- *Resid* ≥ *10y* aOR: 1.11 (0.65-1.88)	
		NHB<br>- US-born<br>- Foreign-born	2699<br>- 1607<br>- 1092	Obesity: US-b 28%, F-b 20%<br>Sec ed or below: US-b 7%, F-b 19%<br>Sm: US-b 20%, F-b 5 %	US-b: 7.8<br>F-b: 7.2	US-b: 12.2<br>F-b: 9.2	[NHB vs NHW]<br>PE aOR: 1.17 (0.87-1.56)<br>[US-b vs F-b]<br>PE aOR: 0.74 (0.55-1.00)<br>- *Resid < 10y* aOR: 0.62 (0.41-0.93)<br>- *Resid* ≥ *10y* aOR: 0.64 (0.38-1.09)	
Gyamfi-Bannerman (2021)	US	NHW	47684	Lowest income: 23%				[E among women with PE]<br>PE: 1.3%<br>[Severe maternal morbidity]<br>PE 6.1%, [ref]
		NHB	20454	Lowest income: 49%				[E among women with PE]<br>PE 1.7%<br>[Severe maternal morbidity]<br>PE 9.8%, aOR: 1.36 (1.28-1.45)<br>[Maternal mortality]<br>[Black vs non-Black]<br>PE 121.8 vs 24.1 /100.000 deliveries, p<0.01
		Hispanic	19557	Lowest income: 38%				[E among women with PE]<br>PE 1.6<br>[Severe maternal morbidity]<br>PE 7.7%, aOR: 1.10 (1.03-1.18)

		Asian / Pacific Islander	3317	Lowest income: 13%				[E among women with PE]<br>PE 1.4<br>[Severe maternal morbidity]<br>PE 7.5%, aOR: 1.18 (1.04-1.34)
		Native American	971	Lowest income: 43%				[Severe maternal morbidity]<br>PE aOR: 1.23 (0.99-1.54)
		Other	4303	Lowest income: 29%				[Severe maternal morbidity]<br>PE aOR: 1.22 (1.09-1.37)
		Unknown	5455	Lowest income: 22%				[Severe maternal morbidity]<br>PE aOR: 1.00 (0.89-1.11)
Shah (2021)	US	NHW<br>- US-born<br>- Foreign-born	4744665<br>- 4431830<br>- 312835	College graduate: US-b 73%, F-b 82%<br>BMI: US-b 24.6, F-b 22.9	[GH/PE]<br>US-b: 9.2<br>F-b: 4.7	US-b: 0.7<br>F-b: 0.4	[F-b vs US-b]<br>GH/PE RR: 0.51 (0.50-0.52)	
		NHB<br>- US-born<br>- Foreign-born	1207480<br>- 1034029<br>- 173451	College graduate: US-b 51%, F-b 68%<br>BMI: US-b 26.4, F-b 24.9	[GH/PE]<br>US-b: 10.5<br>F-b: 7.1	US-b: 1.1<br>F-b: 0.8	[F-b vs US-b]<br>GH/PE RR: 0.68 (0.66-0.69)	
		NH Asian / Pacific Islander<br>- US-born<br>- Foreign-born	690304<br>- 151127<br>- 539177	College graduate: US-b 84%, F-b 84%<br>BMI: US-b 23.4, F-b 22.1	[GH/PE]<br>US-b: 6.0<br>F-b: 3.9	US-b: 0.8<br>F-b: 0.7	[F-b vs US-b]<br>GH/PE RR: 0.65 (0.64-0.67)	
		Asian Indian<br>- US-born<br>- Foreign-born	197834<br>- 19572<br>- 178262	College graduate: US-b 94%, F-b 91%<br>BMI: US-b 23.0, F-b 23.4	[GH/PE]<br>US-b: 5.1<br>F-b: 4.4	US-b: 1.0<br>F-b: 0.6	[F-b vs US-b]<br>GH/PE RR: 0.88 (0.81-0.96)	

	Chinese<br>- US-born<br>- Foreign-born	165051<br>- 22387<br>- 142664	College graduate: US-b 97%, F-b 87%<br>BMI: US-b 22.1, F-b 20.8	[GH/PE]<br>US-b: 4.0<br>F-b: 2.0	US-b: 1.1<br>F-b: 0.4	[F-b vs US-b]<br>GH/PE RR: 0.50 (0.44-0.58)
	Filipino<br>- US-born<br>- Foreign-born	71797<br>- 20760<br>- 51037	College graduate: US-b 88%, F-b 87%<br>BMI: US-b 24.4, F-b 22.8	[GH/PE]<br>US-b: 8.1<br>F-b: 6.9	US-b: 2.3<br>F-b: 1.8	[F-b vs US-b]<br>GH/PE RR: 0.85 (0.80-0.90)
	Japanese<br>- US-born<br>- Foreign-born	17493<br>- 4897<br>- 12596	College graduate: US-b 95%, F-b 91%<br>BMI: US-b 22.9, F-b 20.7	[GH/PE]<br>US-b: 5.8<br>F-b: 2.4	US-b: 1.8<br>F-b: 0.4	[F-b vs US-b]<br>GH/PE RR: 0.42 (0.30-0.61)
	Korean<br>- US-born<br>- Foreign-born	40307<br>- 9630<br>- 30677	College graduate: US-b 96%, F-b 95%<br>BMI: US-b 22.1, F-b 21.5	[GH/PE]<br>US-b: 4.6<br>F-b: 3.6	US-b: 1.0<br>F-b: 0.9	[F-b vs US-b]<br>GH/PE RR: 0.79 (0.62-1.00)
	Vietnamese<br>- US-born<br>- Foreign-born	51114<br>- 11777<br>- 39337	College graduate: US-b 87%, F-b 68%<br>BMI: US-b 22.7, F-b 20.7	[GH/PE]<br>US-b: 4.9<br>F-b: 2.5	US-b: 1.9 F-b: 0.4	[F-b vs US-b]<br>GH/PE RR: 0.51 (0.45-0.58)
	Hispanic / Latina<br>- US-born<br>- Foreign-born	1792676<br>- 1090071<br>- 702605	College graduate: US-b 50%, F-b 41%<br>BMI: US-b 25.6, F-b 24.5	[GH/PE]<br>US-b: 7.8<br>F-b: 6.1	US-b: 0.8<br>F-b: 0.7	[F-b vs US-b]<br>GH/PE RR: 0.77 (0.76-0.78)

		Mexican<br>- US-born<br>- Foreign-born	986397<br>- 657738<br>- 328659	College graduate: US-b 47%, F-b 34%<br>BMI: US-b 25.7, F-b 24.7	[GH/PE]<br>US-b: 7.9<br>F-b: 6.1	US-b: 0.8<br>F-b: 0.8			[F-b vs US-b]<br>GH/PE RR: 0.77 (0.76-0.79)	
		Puerto Rican<br>- US-born<br>- Foreign-born	149388<br>- 114300<br>- 35088	College graduate: US-b 51%, F-b 53%<br>BMI: US-b 25.5, F-b 25.1	[GH/PE]<br>US-b: 8.2<br>F-b: 8.3	US-b: 1.0<br>F-b: 1.2			[F-b vs US-b]<br>GH/PE RR: 1.01 (0.97-1.06)	
		Cuban<br>- US-born<br>- Foreign-born	60794<br>- 25938<br>- 34856	College graduate: US-b 73%, F-b 48%<br>BMI: US-b 25.0, F-b 23.8	[GH/PE]<br>US-b: 7.5<br>F-b: 6.6	US-b: 0.6<br>F-b: 0.6			[F-b vs US-b]<br>GH/PE RR: 0.88 (0.83-0.94)	
		Central / South American<br>- US-born<br>- Foreign-born	284519<br>- 68673<br>- 215846	College graduate: US-b 65%, F-b 44%<br>BMI: US-b 25.6, F-b 24.1	[GH/PE]<br>US-b: 6.8<br>F-b: 5.4	US-b: 0.7<br>F-b: 0.5			[F-b vs US-b]<br>GH/PE RR: 0.79 (0.76-0.82)	
Ananth (2021)	US	White	NR	NR						[HDP-related maternal mortality rate per 100.000 live births]<br>1.4
		Black	NR	NR						[HDP-related maternal mortality rate per 100.000 live births]<br>5.4
Maric (2019)	US	NHW	623306	Obesity: 8%<br>Sm: 5%<br>Ed sec or less: 6%<br>P0: 45%			2.6	0.3		

		NH African American	126155	Obesity: 15%<br>Sm: 5%<br>Ed sec or lower: 16%<br>P0: 42%			4.2		1.0	
		Asian	217144	Obesity: 4%<br>Sm: 0.6%<br>Ed sec or lower: 5%<br>P0: 48%			1.5		0.2	
		Hispanic	1171407	Obesity: 15%<br>Sm: 0.8%<br>Ed sec or lower: 40%<br>P0: 36%			3.0		0.4	
Ananth (2019)	US	Black	20 357 421	NR		1.2				RR: 2.31 (2.30-2.32)
		White	93 632 802	NR		0.5				[ref]
Hu (2019)	US	African American	385,973	NR	[GH/PE]<br>6.6					aRR: 1.25 (1.23 - 1.27)
		White	1,733,300	NR	[GH/PE]<br>4.7					[ref]
Singh (2018)	US	NHW<br>- US-born<br>- Foreign-born	4322169<br>- 4,031,903<br>- 282,556	Obesity: 23%<br>Sm: 12%<br>Ed ≥ 16y: 40%	[HDP]<br>All: 7.2<br>US-b: 7.4<br>F-b: 4.1<br>[GH/PE]<br>5.7	1.5		0.2		[NHW vs Chinese]<br>HDP aOR: 1.92 (1.82-2.00)<br>- *US-b aOR*: 2.77 (2.65-2.90)<br>- *F-b aOR*: 1.73 (1.65-1.82)<br>CH aOR: 1.59 (1.45-1.75)<br>HDP aOR: 1.96 (1.87-2.05)

NHB<br>- US-born<br>- Foreign-born	1188014<br>- 1,004,997<br>- 177,299	Obesity: 35%<br>Sm: 7%<br>Ed ≥ 16y: 16%	[HDP]<br>All: 9.8<br>US-b: 10.3<br>F-b: 7.1<br>[GH/PE]<br>6.6	3.3	0.4	[NHB vs Chinese]<br>HDP aOR: 2.42 (2.32-2.52)<br>- *US-b aOR*: 3.47 (3.31-3.65)<br>- *F-b aOR*: 2.63 (2.50-2.76)<br>CH aOR: 2.98 (2.71-3.27)<br>GH/PE aOR: 2.05 (1.96-2.15)<br>[NHB vs NHW]<br>HDP Sig
American Indian / Alaska Native	75204	Obesity: 36%<br>Sm: 18%<br>Ed ≥ 16y: 9%	[HDP]<br>All: 8.9<br>[GH/PE]<br>6.8	2.2	0.4	[AI/AN vs Chinese]<br>HDP aOR: 2.00 (1.91-2.10)<br>CH aOR: 2.04 (1.84-2.27)<br>GH/PE aOR: 1.96 (1.86-2.07)<br>[AI/AN vs NHW]<br>HDP Sig
Chinese<br>- US-born<br>- Foreign-born	116439<br>- 14,497<br>- 101,753	Obesity: 3%<br>Sm: <1%<br>Ed ≥ 16y: 68%	[HDP]<br>All: 2.2<br>US-b: 4.3<br>F-b: 1.9<br>[GH/PE]<br>1.8	0.4	0.1	[US-b vs F-b]<br>HDP aOR: 2.12 (1.93-2.33)
Japanese<br>- US-born<br>- Foreign-born	14177<br>- 3,893<br>- 10,270	Obesity: 5%<br>Sm: 1%<br>Ed ≥ 16y: 67%	[HDP]<br>All: 3.5<br>US-b: 6,5<br>F-b: 2.3<br>[GH/PE]<br>2.7	0.8	0.2	[Japanese vs Chinese]<br>HDP aOR: 1.50 (1.36-1.66)<br>- *US-b aOR*: 3.02 (2.63-3.46)<br>- *F-b aOR*: 1.28 (1.11-1.46)<br>CH aOR: 1.47 (1.19-1.82)<br>GH/PE aOR: 1.50 (1.34-1.68)<br>[Japanese vs NHW]<br>HDP Sig
Filipino<br>- US-born<br>- Foreign-born	63160<br>- 17,216<br>- 45,845	Obesity: 12%<br>Sm: 1%<br>Ed ≥ 16y: 53%	[HDP]<br>All: 7.7<br>US-b: 8.0<br>F-b: 7.6<br>[GH/PE]<br>5.9	1.9	0.3	[Filipino vs Chinese]<br>HDP aOR: 2.94 (2.79-3.08)<br>- *US-b aOR*: 3.43 (3.19-3.69)<br>- *F-b aOR*: 3.53 (3.33-3.73)<br>CH aOR: 3.43 (3.08-3.82)<br>GH/PE aOR: 2.74 (2.59-2.89)<br>[Filipino vs NHW]<br>HDP Sig
Hawaiian	1725	Obesity: 37%<br>Sm: 12%<br>Ed ≥ 16y: 18%	[HDP]<br>All: 7.0<br>[GH/PE]<br>5.6	1.3	0.5	[Hawaiian vs Chinese]<br>HDP aOR: 1.58 (1.30-1.91)<br>CH aOR: 1.24 (0.81-1.89)<br>GH/PE aOR: 1.67 (1.35-2.07)

Asian Indian<br>- US-born<br>- Foreign-born	131594<br>- 13,323<br>- 118,026	Obesity: 10%<br>Sm: <1%<br>Ed ≥ 16y: 78%	[HDP]<br>All: 4.0<br>US-b: 4.5<br>F-b: 3.9<br>[GH/PE]<br>3.3	0.7	0.1	[Asian Indian vs Chinese]<br>HDP aOR: 1.55 (1.48-1.63)<br>- *US-b aOR*: 1.96 (1.79-2.16)<br>- *F-b aOR*: 1.75 (1.65-1.84)<br>CH aOR: 1.51 (1.35-1.69)<br>GH/PE aOR: 1.56 (1.48-1.64)<br>[Asian Indian vs NHW]<br>HDP Sig
Korean<br>- US-born<br>- Foreign-born	30390<br>- 6,296<br>- 23,313	Obesity: 5%<br>Sm: 1%<br>Ed ≥ 16y: 77%	[HDP]<br>All: 3.6<br>US-b: 3.8<br>F-b: 3.6<br>[GH/PE]<br>2.8	0.8	0.1	[Korean vs Chinese]<br>HDP aOR: 1.52 (1.42-1.64)<br>- *US-b aOR*: 1.89 (1.65-2.17)<br>- *F-b aOR*: 1.78 (1.64-1.94)<br>CH aOR: 1.74 (1.49-2.03)<br>GH/PE aOR: 1.46 (1.35-1.59)<br>[Korean vs NHW]<br>HDP Sig
Vietnamese<br>- US-born<br>- Foreign-born	41211<br>- 7,713<br>- 33,453	Obesity: 4%<br>Sm: 1%<br>Ed ≥ 16y: 40%	[HDP]<br>All: 2.9<br>US-b: 4.6<br>F-b: 2.6<br>[GH/PE]<br>2.4	0.5	0.1	[Viatnamese vs Chinese]<br>HDP aOR: 1.28 (1.19-1.37)<br>- *US-b aOR*: 2.22 (1.97-2.49)<br>- *F-b aOR*: 1.32 (1.22-1.43)<br>CH aOR: 1.19 (1.01-1.40)<br>GH/PE aOR: 1.31 (1.22-1.42)
Samoan	4316	Obesity: 64%<br>Sm: 8%<br>Ed ≥ 16y: 7%	[HDP]<br>All: 8.6<br>[GH/PE]<br>6.8	1.8	0.5	[Samoan vs Chinese]<br>HDP aOR: 1.62 (1.44-1.81)<br>CH aOR: 1.48 (1.16-1.89)<br>GH/PE aOR: 1.66 (1.46-1.89)<br>[Samoan vs NHW]<br>HDP Sig
Other Asian / Pacific Islander<br>- US-born<br>- Foreign-born	140061<br>- 51,424<br>- 87,782	NR	[HDP]<br>All: 4.5<br>US-b: 5.1<br>F-b: 4.1<br>[GH/PE]<br>3.5	0.9	0.2	[Other Asian / Pacific Islander vs Chinese]<br>HDP aOR: 1.55 (1.47-1.62)<br>- *US-b aOR*: 2.09 (1.97-2.22)<br>- *F-b aOR*: 1.78 (1.68-1.88)<br>CH aOR: 1.49 (1.34-1.66)<br>GH/PE aOR: 1.55 (1.47-1.63)
Mexican<br>- US-born<br>- Foreign-born	1092146<br>- 554,774<br>- 536,471	Obesity: 29%<br>Sm: 2%<br>Ed ≥ 16y: 9%	[HDP]<br>All: 5.2<br>US-b: 5.8<br>F-b: 4.5<br>[GH/PE]<br>4.3	0.9	0.2	[Mexican vs Chinese]<br>HDP aOR: 1.48 (1.42-1.54)<br>- *US-b aOR*: 2.06 (1.96-2.16)<br>- *F-b aOR*: 1.70 (1.62-1.79)<br>CH aOR: 1.11 (1.01-1.22)<br>GH/PE aOR: 1.56 (1.49-1.63)<br>[Mexican vs NHW]

									HDP Sig
		Puerto Rican<br>- mainland US-born<br>- Puerto Rico-born	140866<br>- 121,554<br>- 18,530	Obesity: 30%<br>Sm: 7%<br>Ed ≥ 16y: 15%	[HDP]<br>All: 6.4<br>US-b: 6.3<br>PR-b: 7.0<br>[GH/PE]<br>4.7	1.6		0.2	[Puerto Rican vs Chinese]<br>HDP aOR: 1.70 (1.62-1.78)<br>- *US-b aOR*: 2.34 (2.22-2.46)<br>- *PR-b aOR*: 2.55 (2.37-2.74)<br>CH aOR: 1.67 (1.50-1.85)<br>GH/PE aOR: 1.67 (1.58-1.75)<br>[Puerto Rican vs NHW]<br>HDP Sig
		Cuban<br>- US-born<br>- Foreign-born	41270<br>- 19,864<br>- 21,389	Obesity: 20%<br>Sm: 2%<br>Ed ≥ 16y: 27%	[HDP]<br>All: 5.9<br>US-b: 6.2<br>F-b: 5.6<br>[GH/PE]<br>4.8	1.1		0.1	[Cuban vs Chinese]<br>HDP aOR: 1.82 (1.72-1.93)<br>- *US-b aOR*: 2.29 (2.13-2.47)<br>- *F-b aOR*: 2.34 (2.17-2.52)<br>CH aOR: 1.40 (1.23-1.60)<br>GH/PE aOR: 1.88 (1.76-2.00)<br>[Cuban vs NHW]<br>HDP Sig
		Central / South American<br>- US-born<br>- Foreign-born	278905<br>- 47,914<br>- 230,721	Obesity: 21%<br>Sm: 1%<br>Ed ≥ 16y: 18%	[HDP]<br>All: 4.5<br>US-b: 5.3<br>F-b: 4.4<br>[GH/PE]<br>3.6	1.0		0.2	[C/SA vs Chinese]<br>HDP aOR: 1.61 (1.54-1.68)<br>- *US-b aOR*: 2.08 (1.95-2.21)<br>- *F-b aOR*: 1.84 (1.75-1.94)<br>CH aOR: 1.33 (1.21-1.47)<br>GH/PE aOR: 1.64 (1.56-1.72)<br>[C/SA vs NHW]<br>HDP Sig
		Other Hispanic<br>- US-born<br>- Foreign-born	284926<br>- 202,960<br>- 81,468	NR	[HDP]<br>All: 6.0<br>US-b: 6.0<br>F-b: 5.9<br>yy<br>4.9	1.1		0.2	[Other Hispanic vs Chinese]<br>HDP aOR: 1.69 (1.61-1.76)<br>- *US-b aOR*: 2.21 (2.10-2.32)<br>- *F-b aOR*: 2.41 (2.28-2.54)<br>CH aOR: 1.40 (1.27-1.54)<br>GH/PE aOR: 1.72 (1.64-1.80)<br>[Other Hispanic vs NHW]<br>HDP Sig
Zamora (2016)	US	White	62987	Obesity: 20%<br>Sm: 11%<br>Ed < high school 12%			7.9		[ref]

		American Indian / Alaska Native	7109	Obesity: 28%<br>Sm: 20%<br>Ed < high school: 36%			8.9	aOR: 1.05 (0.95-1.16)
Haghighat (2016)	US	Asian	8489	NR			2.7	p<0.01
		Black	5576	NR			4.1	[ref]
		Hispanic	22995	NR			3.2	p<0.01
		NHW	23400	NR			3.0	
		Other	1187	NR			4.1	
		Unknown	553	NR			2.4	
Masho (2016)	US	NHW	162742	NR	12.5			[ref]
		NHB	37793	NR	15.8			OR: 1.31 (1.25-1.37)
		Hispanic	38564	NR	9.1			OR: 0.69 (0.65-0.74)
		Other NH	18253	NR	8.9			OR: 0.69 (0.64-0.74)
Nakagawa (2016)	Hawaii	Chinese	9289	Sm: 1%<br>Obesity: <1%<br>P0: >99%		0.1	2.0	PE aOR: 0.64, p<0.001
		Filipino	53122	Sm: 2%<br>Obesity: 1%<br>P0: 99%		0.3	4.6	PE aOR: 1.55, p<0.001
		Native Hawaiian	62933	Sm: 4%<br>Obesity: 2%<br>P0: 99%		0.3	4.6	PE aOR: 1.54, p<0.001
		Japanese	30992	Sm: 2%<br>Obesity: <1%<br>P0: >99%		0.3	2.8	PE aOR: 0.91, p=0.077

		Other Asian	13853	Sm: 2%<br>Obesity: 1%<br>P0: >99%		0.2	2.5		PE aOR: 0.88, p=0.076	
		Other Pacific Islanders	22245	Sm: 4%<br>Obesity: 5%<br>P0: 97%		0.2	4.5		PE aOR: 1.40, p<0.001	
		Other	27876	Sm: 2%<br>Obesity: 1%<br>P0: 99%		0.2	4		PE aOR: 1.32, p<0.001	
		White	51259	Sm: 2%<br>Obesity: 1%<br>P0: >99%		0.2	2.9		[ref]	
Shahul (2015)	US	White	568124	Income <p25: 22%			3.8		[overall]<br>p<0.001	[ref]
		Black	235007	Income <p25: 50%			6.0			[Maternal inpatient mortality]<br>aOR: 2.85 (1.38-5.53)<br>[IUFD]<br>aOR: 2.45 (2.14-2.82)
		Hispanic	268014	Income <p25: 40%			2.6			[Maternal inpatient mortality]<br>aOR: 1.44 (0.74-2.79)<br>[IUFD]<br>aOR: 0.96 (0.82-1.13)
Chang (2014)	US	NHW	1718634	Sm: 13%	[GH/PE] 4.5	1.3		0.3		
		NHB	402472	Sm: 8%	[GH/PE] 5.1	2.8		0.4		
		NH American Indian	34348	Sm: 19%	[GH/PE] 5.3	1.8		0.6		
		NH Asian / Pacific Islander	192141	Sm: 2%	[GH/PE] 2.4	0.8		0.2		

		Hispanic	765569	Sm: 2.%	[GH/PE]<br>3.1	0.7			0.2		
Ghosh<br>(2014)	US	NHW	304999	Sm: 7 %		0.8	4.5	Mild: 3.2<br>Sev: 1.6	0.1	0.3	[ref]
		NHB	11584	Sm: 5%		1.8	3.6	Mild: 4.8<br>Sev: 3.5	0.1	1	CH aOR: 1.43 (1.11–1.84)<br>GH aOR: 0.80 (0.69–0.92)<br>mPE aOR: 1.26 (1.10-1.45)<br>sevPE aOR: 1.31 (1.10-1.57)<br>E aOR: 0.81 (0.27-2.42)<br>sPE aOR: 1.98 (1.40-2.80)
		Hispanic	10476	Sm: 3%		0.7	2.4	Mild: 3.5<br>Sev: 2.4	0.1	0.5	CH aOR: 0.66 (0.48–0.90)<br>GH aOR: 0.66 (0.57–0.78)<br>mPE aOR: 1.10 (0.95-1.26)<br>sevPE aOR: 0.83 (0.69-1.01)<br>E aOR: 0.73 (0.24-2.20)<br>sPE aOR: 1.08 (0.72-1.62)
		Asian / Pacific Islander	2696	Sm: 2%		0.3	1.7	Mild: 2.0<br>Sev: 1.7	0.1	0.2	CH aOR: 0.49 (0.25–0.96)<br>GH aOR: 0.61 (0.45–0.83)<br>mPE aOR: 0.62 (0.46-0.82)<br>sevPE aOR: 0.96 (0.68-1.35)<br>E aOR: 0.96 (0.12-7.73)<br>sPE aOR: 0.53 (0.19-1.45)
		Multiracial / other	1362	Sm: 5%		1.0	2.0	Mild: 3.0<br>Sev: 2.0	0.1	0.8	CH aOR: 0.67 (0.37–1.22)<br>GH aOR: 0.71 (0.47–1.06)<br>mPE aOR: 0.92 (0.66-1.29)<br>sevPE aOR: 0.92 (0.64-1.31)<br>E aOR: 0.89 (0.10-8.24)<br>sPE aOR: 1.32 (0.66-2.66)
Liu<br>(2014)	US	NHW	77980	NR	[GH/PE]<br>5.8						[ref]
		NHB	43671	NR	[GH/PE]<br>6.3						Underweight aOR: 1.64 (1.14-2.36)<br>Normal weight aOR 1.28 (1.15-1.42)<br>Overweight aOR: 0.97 (0.85-1.10)<br>Obese aOR: 0.96 (0.88, 1.05)

		Hispanic	12198	NR	[GH/PE]<br>3.5				Underweight aOR: 0.53 (0.23-1.24)<br>Normal weight aOR 0.86 (0.7-1.02)<br>Overweight aOR: 0.61 (0.48-0.77)<br>Obese aOR: 0.66 (0.55-0.79)	
Marshall (2014)	US	Caucasian	258934	High 55%<br>Sm: 20 %<br>BMI ≥ 30: 19%<br>P0: 39%			8.0			[ref]
		African American	43523	High 34%<br>Sm: 12 %<br>BMI ≥ 30: 27%<br>P0: 38%			7.3			[Neonatal composite morbidity]<br>*Obese women* aOR: 1.1 (1.1-1.2)
Gold (2014)	US	Hispanic	[GH/PE]<br>560572<br>[E]<br>1716992	NR	[GH/PE]<br>P0 ≤ 19y: 3.5-3.6<br>P1+ ≤ 19y: 1.8-3.0			P0 ≤ 19y: 0.3-0.4<br>P1+ ≤ 19y: 0.2-0.4	[ref]	
		NHW	[GH/PE]<br>661540<br>[E]<br>2354436	NR	[GH/PE]<br>P0 ≤ 19y: 4.9-5.5<br>P1+ ≤ 19y: 2.9-4.3			P0 ≤ 19y: 0.4-0.5<br>P1+ ≤ 19y: 0.2-0.3	[NHW vs Hispanic]<br>GH/PE Sig (except P1+ < 15y)<br>E Sig (except P1+ < 15y)	
		NHB	[GH/PE]<br>400997<br>[E]<br>1397137	NR	[GH/PE]<br>P0 ≤ 19y: 5.1-5.5<br>P2+ ≤ 19y: 2.9-3.7			P0 ≤ 19y: 0.5-0.7<br>P1+ ≤ 19y: 0.3-0.5	[NHB vs Hispanic]<br>GH/PE Sig (except P1+ < 15y)<br>[NHB vs NHW]<br>E Sig (except P1+ < 15y)	
Breathett (2014)	US	African American	111309	NR			5.6		POR: 1.52 (1.50-1.55)	

		Caucasian	433933	NR	3.4		[ref]	
Lisonkova (2013)	US	NHW	323552	NR	<34w: 0.4<br>≥34w: 2.8		[ref]	
		African American	20045	NR	<34w: 0.8<br>≥34w: 3.6		*<34w* aHR: 1.75 (1.45–2.12)<br>*≥34w* aHR: 1.20 (1.11–1.31)	
		Hispanic	56615	NR	<34w: 0.4<br>≥34w: 2.8		*<34w* aHR: 1.07 (1.01–1.13)<br>*≥34w* aHR: 1.07 (1.01–1.13)	
		Native American	10625	NR	<34w: 0.4<br>≥34w: 3.6		*<34w* aHR: 1.36 (1.22–1.51)<br>*≥34w* aHR: 1.36 (1.22–1.51)	
		Other	44032	NR	<34w: 0.4<br>≥34w: 2.0		*<34w* aHR: 0.98 (0.82–1.16)<br>*≥34w* aHR: 0.68 (0.63–0.73)	
Fong (2013)	US	Caucasian	1057420	NR		0.1	[ref]	
		Black	121017	NR		0.1	aOR: 1.81 (1.51-2.17)	
		Native American / Eskimo / Aleut	3687	NR		0.1	aOR: 0.73 (0.18-2.95)	
		Asian / Pacific Islander	241582	NR		<0.1	aOR: 0.83 (0.67-1.04)	
		Hispanic	1347165	NR		0.1	aOR: 1.27 (1.14-1.42)	
Cripe (2012)	US	Cambodian	3489	Ed > 12y: 29%<br>Sm: 2.5%<br>P0: 33%	2.8		[Cambodian / Laotian / Vietnamese vs Japanese] p=0.41<br>[Cambodian / Laotian / Vietnamese / Japanese vs White] p<0.001	[PTB] [Cambodian vs Japanese] aOR: 3.06 (0.89-9.45)<br>[Cambodian vs White] aOR: 1.21 (0.59-2.50)<br>[LBW] [Cambodian vs Japanese] aOR: 1.81 (0.67-4.88)<br>[Cambodian vs White] aOR: 2.34 (1.22-4.49)<br>[Neonatal stay>72h] [Cambodian vs Japanese] aOR: 1.38 (0.58-3.29)<br>[Cambodian vs White] aOR: 2.09 (1.11-3.92)

		Laotian	2038	Ed >12y: 29%<br>Sm: 2.2%<br>P0: 34%	2.6		[PTB] [Laotian vs Japanese] aOR: 5.2 (1.63-16.58)<br>[Laotian vs White] aOR: 2.23 (1.00-4.94)<br>[LBW] [Laotian vs Japanese] aOR: 2.55 (0.85-7.66)<br>[Laotian vs White] aOR: 2.97 (1.32-6.69)<br>[Neonatal stay>72h] [Laotian vs Japanese] aOR: 2.02 (0.77-5.29)<br>[Laotian vs White] aOR: 3.18 (1.61-6.28)
		Vietnamese	11618	Ed > 12y: 38%<br>Sm: 0.9%<br>P0: 46%	2.3		[PTB] [Vietnamese vs Japanese] aOR: 2.70 (1.04-6.99)<br>[Vietnamese vs White] aOR: 1.10 (0.73-1.65)<br>[LBW] [Vietnamese vs Japanese] aOR: 1.04 (0.45-2.42)<br>[Vietnamese vs White] aOR: 1.18 (0.76-1.85)<br>[Neonatal stay>72h] [Vietnamese vs Japanese] aOR: 0.98 (0.49-1.95)<br>[Vietnamese vs White] aOR: 1.35 (0.92-1.98)
		Japanese	3083	Ed > 12y: 80%<br>Sm: 2.9%<br>P0: 52%	2.8	[ref]	[ref]
		US-born White	33088	Ed > 12y: 56%<br>Sm: 16.2 %<br>P0: 42%	6.2	[ref]	[ref]
Gong (2012)	US	NHW	264210	NR	2.0	[ref]	
		African-American	138818	NR	4.6	[AA vs NHW] aOR: 2.3 (2.2-2.3)	
		North Africa	5108	NR	2.2	[North Africa vs NHW] aOR: 1.1 (0.9-1.3)<br>[F-b vs US-b] aOR: 0.6 (0.3-1.3)	
		Morocco	*1279*	*NR*	*1.5*	*[Morocco vs NHW] aOR: 0.7 (0.4-1.1)*	

	Egypt	*3190*	*NR*	*2.2*	*[Egypt vs NHW]*<br>*aOR: 1.1 (0.9-1.4)*
	Other North Africa	*639*	*NR*	*3.8*	*[Other North Africa vs NHW]*<br>*aOR: 1.9 (1.3-2.9)*
	Sub-Saharan Africa	16344	NR	3.5	[Sub-Saharan Africa vs NHW]<br>aOR: 1.8 (1.6-1.9)<br>[F-b vs US-b]<br>aOR: 1.0 (0.5-1.9)
	Nigeria	*3222*	*NR*	*3.6*	*[Nigeriavs NHW]*<br>*aOR: 1.8 (1.5-2.2)*
	Ghana	*2794*	*NR*	*3.8*	*[Ghana vs NHW]*<br>*aOR: 1.8 (1.5-2.2)*
	Guinea	*1394*	*NR*	*2.7*	*[Guinea vs NHW]*<br>*aOR: 1.5 (1.1-2.1)*
	Senegal	*1286*	*NR*	*2.9*	*[Senegal vs NHW]*<br>*aOR: 1.5 (1.1-2.0)*
	Gambia	*1164*	*NR*	*2.9*	*[Gambia vs NHW]*<br>*aOR: 1.9 (1.3-2.6)*
	Ivory Coast	*1001*	*NR*	*4.2*	*[Ivory Coast vs NHW]*<br>*aOR: 2.1 (1.6-2.9)*
	Mali	*1049*	*NR*	*3.9*	*[Mali vs NHW]*<br>*aOR: 2.0 (1.5-2.8)*
	Other West Africa	*1307*	*NR*	*4.2*	*[Other West Africa vs NHW]*<br>*aOR: 2.2 (1.7-2.9)*
	Central / East / Southern Africa	*3127*	*NR*	*3.3*	*[Central / East / Southers Africa vs NHW]*<br>*aOR: 1.6 (1.3-2.0)*
	East Asia	54458	NR	1.4	[East Asia vs NHW]<br>aOR: 0.8 (0.7-0.8)<br>[F-b vs US-b]<br>aOR: 1.0 (0.7-1.4)
	China	*40553*	*NR*	*1.4*	*[China vs NHW]*<br>*aOR: 0.8 (0.7-0.8)*<br>*[F-b vs US-b]*<br>*aOR: 1.0 (0.7-1.4)*

	Hong Kong	*1100*	*NR*	*1.2*	*[Hong Kong vs NHW]*<br>*aOR: 0.6 (0.4-1.1)*
	Taiwan	*1072*	*NR*	*0.9*	*[Taiwan vs NHW]*<br>*aOR: 0.5 (0.3-0.9)*
	Korea	*7769*	*NR*	*1.4*	*[Korea vs NHW]*<br>*aOR: 0.8 (0.6-0.9)*
	Japan	*2941*	*NR*	*1.2*	*[Japan vs NHW]*<br>*aOR: 0.6 (0.4-0.9)*
	Other East Asia	*1023*	*NR*	*2.4*	*[Other East Asia vs NHW]*<br>*aOR: 1.3 (0.8-1.9)*
	Southeast Asia / Pacific Islands	11117	NR	3.3	[Southeast Asia / Pacific Islands vs NHW]<br>aOR: 1.8 (1.7-2.1)<br>[F-b vs US-b]<br>aOR: 1.8 (1.0-3.1)
	Vietnam	*1748*	*NR*	*1.7*	*[Vietnam vs NHW]*<br>*aOR: 0.9 (0.6-1.4)*
	Malaysia	*742*	*NR*	*1.9*	*[Malaysia vs NHW]*<br>*aOR: 1.0 (0.6-1.7)*
	Philippines	*7249*	*NR*	*4.0*	*[Philippines vs NHW]*<br>*aOR: 2.3 (2.1-2.6)*<br>*[F-b vs US-b]*<br>*aOR: 2.2 (1.2-4.0)*
	Other Southeast Asia	*1378*	*NR*	*1.9*	*[Other Southeast Asia vs NHW]*<br>*aOR: 1.0 (0.7-1.5)*
	South Central Asia	29966	NR	2.2	[South Central Asia vs NHW]<br>aOR: 1.3 (1.2-1.4)<br>[F-b vs US-b]<br>aOR: 1.0 (0.7-1.6)
	India	*12543*	*NR*	*2.2*	*[India vs NHW]*<br>*aOR: 1.2 (1.1-1.4)*<br>*[F-b vs US-b]*<br>*aOR: 0.9 (0.5-1.5)*
	Bangladesh	*6486*	*NR*	*2.7*	*[Bangladesh vs NHW]*<br>*aOR: 1.6 (1.4-1.9)*

	Pakistan	*6899*	*NR*	*2.5*	*[Pakistan vs NHW]*<br>*aOR: 1.4 (1.2-1.6)*
	Afghanistan	*1182*	*NR*	*1.4*	*[Afghanistan vs NHW]*<br>*aOR: 0.8 (0.5-1.3)*
	Iran	*1405*	*NR*	*0.6*	*[Iran vs NHW]*<br>*aOR: 0.3 (0.2-0.7)*
	Other South Central Asia	*1451*	*NR*	*1.7*	*[Other South Central Asia vs NHW]*<br>*aOR: 1.0 (0.6-1.4)*
	NH Caribbean	67023	NR	4.2	[NH Caribbean vs NHW]<br>aOR: 2.0 (1.9-2.1)<br>[F-b vs US-b]<br>aOR: 1.0 (0.8-1.2)
	Jamaica	*24014*	*NR*	*4.1*	*[Jamaica vs NHW]*<br>*aOR: 2.0 (1.9-2.2)*<br>*[F-b vs US-b]*<br>*aOR: 0.9 (0.6-1.2)*
	Haiti	*14372*	*NR*	*4.7*	*[Haiti vs NHW]*<br>*aOR: 2.2 (2.0-2.4)*<br>*[F-b vs US-b]*<br>*aOR: 1.1 (0.8-1.4)*
	Trinidad and Tobago	*10999*	*NR*	*3.9*	*[Trinidad and Tobago vs NHW]*<br>*aOR: 1.9 (1.7-2.1)*
	Grenada	*2256*	*NR*	*3.9*	*[Grenada vs NHW]*<br>*aOR: 1.8 (1.5-2.2)*
	Barbados	*2129*	*NR*	*4.2*	*[Barbados vs NHW]*<br>*aOR: 1.9 (1.5-2.4)*
	St Vincent	*1529*	*NR*	*3.7*	*[St Vincent vs NHW]*<br>*aOR: 1.7 (1.3-2.2)*
	Antigua and Barbuda	*1277*	*NR*	*4.0*	*[Antigua and Barbuda vs NHW]*<br>*aOR: 1.9 (1.4-2.5)*
	St Lucia	*1019*	*NR*	*4.0*	*[St Lucia vs NHW]*<br>*aOR: 1.9 (1.4-2.7)*

	Virgin Island	*626*	*NR*	*4.2*	*[Virgin Island vs NHW]*<br>*aOR: 2.1 (1.4-3.2)*
	Other NH Caribbean	*8802*	*NR*	*4.2*	*[Other NH Caribbean vs NHW]*<br>*aOR: 2.1 (1.9-2.4)*
	Hispanic Caribbean	170567	NR	3.9	[Hispanic Caribbean vs NHW]<br>aOR: 2.0 (2.0-2.1)<br>[F-b vs US-b]<br>aOR: 1.1 (1.0-1.2)
	Dominican Republic	*78856*	*NR*	*4.2*	*[Dominican Republic vs NHW]*<br>*aOR: 2.3 (2.2-2.4)*<br>*[F-b vs US-b]*<br>*aOR: 1.1 (1.0-1.2)*
	Puerto Rico	*89232*	*NR*	*3.7*	*[Puerto Rico vs NHW]*<br>*aOR: 1.9 (1.8-2.0)*<br>*[F-b vs US-b]*<br>*aOR: 1.0 (0.9-1.1)*
	Cuba	*2479*	*NR*	*2.5*	*[Cuba vs NHW]*<br>*aOR: 1.2 (0.9-1.5)*<br>*[F-b vs US-b]*<br>*aOR: 1.0 (0.6-1.7)*
	Mexico	42146	NR	5.0	[Mexico vs NHW]<br>aOR: 2.9 (2.7-3.1)<br>[F-b vs US-b]<br>aOR: 0.9 (0.7-1.2)
	South America	60247	NR	3.3	[South America vs NHW]<br>aOR: 1.8 (1.7-1.9)<br>[F-b vs US-b]<br>aOR: 1.0 (0.8-1.1)
	Guyana	*42146*	*NR*	*3.3*	*[Guyana vs NHW]*<br>*aOR: 1.8 (1.6-2.0)*
	Ecuador	*18125*	*NR*	*3.4*	*[Ecuador vs NHW]*<br>*aOR: 2.0 (1.8-2.1)*<br>*[F-b vs US-b]*<br>*aOR: 1.0 (0.8-1.3)*

	Colombia	*20765*	*NR*	*3.4*	*[Colombia vs NHW]*<br>*aOR: 1.7 (1.5-1.9)*<br>*[F-b vs US-b]*<br>*aOR: 0.9 (0.6-1.2)*
	Peru	*10347*	*NR*	*3.3*	*[Peru vs NHW]*<br>*aOR: 1.7 (1.4-2.0)*
	Brazil	*3983*	*NR*	*3.1*	*[Brazil vs NHW]*<br>*aOR: 1.5 (1.1-2.0)*
	Argentina	*1663*	*NR*	*2.6*	*[Argentina vs NHW]*<br>*aOR: 1.3 (0.9-1.8)*
	Venezuela	*1446*	*NR*	*3.3*	*[Venzuela vs NHW]*<br>*aOR: 1.7 (1.2-2.2)*
	Other South America	*1326*	*NR*	*2.8*	*[Outher South America vs NHW]*<br>*aOR: 1.4 (1.1-1.8)*
	Central America	21980	NR	3.7	[Central America vs NHW]<br>aOR: 2.0 (1.8-2.1)<br>[F-b vs US-b]<br>aOR: 1.2 (0.9-1.5)
	Honduras	*6882*	*NR*	*3.7*	*[Honduras vs NHW]*<br>*aOR: 2.0 (1.8-2.3)*<br>*[F-b vs US-b]*<br>*aOR: 0.9 (0.6-1.3)*
	El Salvador	*5873*	*NR*	*3.3*	*[El Salvador vs NHW]*<br>*aOR: 1.8 (1.5-2.1)*
	Guatemala	*3368*	*NR*	*3.4*	*[Guatemala vs NHW]*<br>*aOR: 1.9 (1.6-2.3)*
	Panama	*2763*	*NR*	*4.5*	*[Panama vs NHW]*<br>*aOR: 2.2 (1.8-2.7)*<br>*[F-b vs US-b]*<br>*aOR: 1.6 (1.0-2.7)*
	Belize	*1045*	*NR*	*4.1*	*[Belize vs NHW]*<br>*aOR: 2.1 (1.6-2.9)*
	Nicaragua	*1154*	*NR*	*3.9*	*[Nicaragua vs NHW]*<br>*aOR: 2.1 (1.5-2.8)*

		Other Central America	*895*	*NR*		*4.5*		*[Other Central America vs NHW]*<br>*aOR: 2.3 (1.6-3.1)*	
		Other Hispanic	13930	NR		3.4		[Other Hispanic vs NHW]<br>aOR: 1.7 (1.6-1.9)	
		Other ethnicity	6180	NR		3.3		[Other ethnicity vs NHW]<br>aOR: 1.6 (1.4-1.9)	
Miranda (2010)	US	NHW	NR	NR	[GH/PE]<br>8.4				[ref]
		NHB	NR	NR	[GH/PE]<br>8.7				[LBW]<br>aOR: 2.24 (2.00-2.51)<br>[PTB]<br>aOR: 1.73 (1.40-2.13)
		Hispanic	NR	NR	[GH/PE]<br>5.5				[LBW]<br>aOR: 1.48 (1.37-1.60)<br>[PTB]<br>aOR: NS
Wong (2008)	US	Chinese	31803	Sm: <1%<br>Ed: 14y<br>P0: 51%	[GH/PE]<br>1.1		0.1	[ref]	
		Japanese	7660	Sm: 3%<br>Ed: 15y<br>P0: 52%	[GH/PE]<br>1.8		0.2	GH/PE aOR: 1.44 (1.14-1.83)<br>E aOR: 2.30 (1.15-4.63)	
		Filipino	30725	Sm: 2%<br>Ed: 15y<br>P0: 43%	[GH/PE]<br>2.9		0.2	GH/PE aOR: 2.80 (2.39-3.28)<br>E aOR: 3.03 (1.77-5.19)	
		Asian Indian	20074	Sm: <1%<br>Ed: 15y<br>P0: 52%	[GH/PE]<br>1.8		0.1	GH/PE aOR: 1.75 (1.49-2.06)<br>E aOR: 1.24 (0.60-2.11)	
		Korean	10710	Sm: 3%<br>Ed: 15y<br>P0: 51%	[GH/PE]<br>1.4		0.1	GH/PE aOR: 1.55 (1.22-1.98)<br>E aOR: 1.05 (0.39-2.83)	

		Vietnamese	16235	Sm: 1%<br>Ed: 13y<br>P0: 46%	[GH/PE]<br>1.0				0.1		GH/PE aOR: 1.06 (0.82-1.37)<br>E aOR: 1.41 (0.63-3.16)
		Soamoan	1835	Sm: 13%<br>Ed: 12y<br>P0: 34%	[GH/PE]<br>2.7				0.3		GH/PE aOR: 3.90 (2.63-5.79)<br>E aOR: 6.13 (2.23- 16.87)
		Guamanian	30353	Sm: 9%<br>Ed: 13y<br>P0: 28%	[GH/PE]<br>2.3				0.3		GH/PE aOR: 2.21 (1.88-2.60)<br>E aOR: 4.15 (2.46-7.0)
		Hawaiian	1302	Sm: 4%<br>Ed: 13y<br>P0: 43%	[GH/PE]<br>2.9				0.2		GH/PE aOR: 1.92 (1.30-2.85)<br>E aOR: 1.77 (0.51-6.17)
Yeo (2007)	US	Hispanic	559	NR	[GH/PE]<br>1.3						RR: 0.24
		Non-Hispanic	2241	NR	[GH/PE]<br>5.3						[ref]
Tanaka (2007)	US	White	1297460	NR		1.1	1.8	2.0		0.2	
		Other	512653	NR		1.1	1.2	2.3		0.3	
		Hispanic	310858	NR		0.9	1.2	3.0		0.3	
		Black	450098	NR		2.1	1.5	3.3		0.7	
Rao (2006-2)	US	Chinese	1054	P0: 57%<br>Ed: 18y	[GH/PE]<br>1.8						[Chinese vs all others]<br>OR: 0.47 (0.28-0.79)
		Filipino	618	P0: 50%<br>Ed: 15y	[GH/PE]<br>6.3						[Filipino vs all others]<br>OR: 2.21 (1.49-3.29)
		Indian / Pakistani	1015	P0: 58%<br>Ed: 18y	[GH/PE]<br>3.3						[Indian / Pakistani vs all others]<br>OR: 1.16 (0.76-1.78)
		Japanese	272	P0: 51%<br>Ed: 17y	[GH/PE]<br>1.8						[Japenese vs all others]<br>OR: 0.57 (0.23-1.42)

		Korean	170	P0: 59%<br>Ed: 17y	[GH/PE]<br>1.2					[Korean vs all others]<br>OR: 0.38 (0.09-1.58)	
		Pacific Islander	545	P0: 29%<br>Ed: 12y	[GH/PE]<br>4.8					[Pacific Islander vs all others]<br>OR: 1.27 (0.68-2.39)	
		Vietnamese	105	P0: 62%<br>Ed: 16y	[GH/PE]<br>1.0					[Viatnamese vs all others]<br>OR: 0.26 (0.04-1.91)	
Rao (2006-1)	US	Japanese	617	Obesity: 2%		2.1		3.7		[overall]<br>CH p<0.52<br>[ref]	
		Chinese	3201	Ed some college 76%		1.6		4.0		PE aOR: 1.53 (0.81-2.92)	
		Filipino	2693	P0: 56%		1.9		6.8		PE aOR: 2.78 (1.48-5.24)	
Odell (2006)	US	African American	12258	P0: 40%<br>Ed <12y: 26%<br>Income <$30,000 per year: 29%<br>Sm: 16%		2.1		3.3			
		Haitian	4320	P0: 39%<br>Ed <12y: 15%<br>Income <$30,000 per year: 6%<br>Sm: 1%		2.7		3.1			
Bryant (2005)	US	White	981	P0: 64%	7.4	2.0	2.5	2.9	0.3		[SBP≥160 mmHG among GH/PE]<br>7%, [ref]<br>[Sev among GH/PE]<br>36%, [ref]
		Black	374	P0: 46%	7.4	1.3	1.3	4.8	0.8		[SBP≥160 mmHG among GH/PE]<br>43%, p=0.01<br>[Sev among GH/PE]<br>48%, p=0.4

Wolf (2004)	US	Caucasian	2381	Sm: 37%<br>Income $58604+-20879<br>Ed 16+-3 y			8.5	3.7			[ref]	
		Hispanic	863	Sm: 46%<br>Income $34548+-9579<br>Ed 11+-4 y			1.6	3.8			PE aRR: 1.9 (1.1-3.3)<br>GH aRR: 0.39 (0.22-0.72)	
Zhang (2003)	US	African American	NR	NR				Sev: 0.8	0.2	0.4	- Greater risk of most severe complications and operative procedures;<br>- stillbirth rate more than doubled.	[PTB] 34%
		Caucasian	NR	NR				Sev: 0.6	0.1	0.1	[ref]	[PTB] 29%
Samadi (1996)	US	African American	1770959	NR	[HDP] 6.4<br>[GH/PE] 3.9	2.5	1.3	Mild: 1.8<br>Sev: 0.7	NR		HDP NS<br>GH/PE NS<br>CH Sig<br>PE NS	Risk of PTB and FGR increased in all HDP, regardless of race.
		Other	10610636	NR	[HDP] 4.9<br>[GH/PE] 3.8	1.1	1.5	Mild: 1.7<br>Sev: 0.5	0.1		[ref]	
Urquia (2012)	Canada	Industrialized nations, not Canada	18161	P0: 44%<br>Income, lowest quintile: 16%<br>No high school: 52%				Sev: 0.2			[ref]	
		Central / Eastern Europe	8835	P0: 44%<br>Income, lowest quintile: 22%<br>No high school: 34%				Sev: 0.2			aOR: 1.21 (0.69-2.11)	

	Hispanic America	7098	P0: 40%<br>Income, lowest quintile: 31%<br>No high school: 65%	Sev: 0.6	aOR: 3.11 (1.97-4.88)
	Caribbean	15218	P0: 38%<br>Income, lowest quintile: 40%<br>No high school: 75%	Sev: 0.7	aOR: 3 .34 (2.25-4.96)
	Sub-Saharan Africa	9130	P0: 28%<br>Income, lowest quintile: 53%<br>No high school: 54%	Sev: 0.7	aOR: 3.14 (2.04-4.83)
	Middle East / North Africa	8552	P0: 31%<br>Income, lowest quintile: 33%<br>No high school: 44%	Sev: 0.2	aOR: 1.00 (0.56-1.80)
	East Asia / Pacific	21581	P0: 41%<br>Income, lowest quintile: 27%<br>No high school: 45%	Sev: 0.3	aOR: 1.59 (1.05-2.41)
	South Asia	30274	P0: 30%<br>Income, lowest quintile: 33%<br>No high school: 44%	Sev: 0.3	aOR: 1.30 (0.86-1.97)
**OCEANIA**					

Al-Rubaie (2020)	Australia	Australian / New Zealand born English speakers	15422	NR		4.8	[ref]
		Middle Eastern / African	6977	NR		2.6	aOR: 0.55 (0.47–0.66)<br>- *<34w* aOR: 0.45 (0.24-0.86)
		Southeast Asian	2725	NR		4.0	aOR: 0.98 (0.79–1.23)
		Northeast Asian	4470	NR		1.1	aOR: 0.33 (0.25–0.45)<br>- *<37w* aOR: 0.59 (0.36–0.96)
		Southern Asian	8762	NR		2.6	aOR: 0.73 (0.62–0.85)
		Sub-Saharan African	1030	NR		3.5	aOR: 0.63 (0.44–0.90)
		Latin American / Caribbean	290	NR		3.4	aOR: 0.64 (0.33–1.26)
		Aboriginal / Torres Strait Islanders	774	NR		5.7	aOR: 1.28 (0.92-1.78)
Dahlen (2013)	Australia	Australian-born	496668	Sm: 18.3%<br>P0: 41.2%	6.0		[Australia-born vs non-Australia-born] p<0.001
		New Zealand-born	17293	Sm: 22.6%<br>P0: 39.6%	6.5		
		England-born	15218	Sm: 8.3%<br>P0: 45.6%	6.3		
		China-born	14526	Sm: 0.5%<br>P0: 50%	2.8		
		Vietnam-born	13835	Sm: 1.3%<br>P0: 43.2%	2.5		
		Lebanon-born	12451	Sm: 8.6%<br>P0: 27.7%	3.1		

		Philippines-born	9684	Sm: 3.9%<br>P0: 40.5%	6.8								
		India-born	8301	Sm: 1.6%<br>P0: 52.9%	4.6								
		Born in other country	103761	Sm: 4.8%<br>P0: 43.3%	4.8								
Anderson (2012)	New Zealand	European	13079	NR				3.1			[ref]		
		Māori	1913	NR				4.7			aOR: 1.51 (1.16-1.96)		
		Pacific	3685	BMI 32.0 (27.4–36.9)				4.5			aOR: 1.25 (0.99-1.57)		
		Chinese	3148	BMI 21.3 (19.6–23.4)				1.5			aOR: 0.56 (0.41-0.76)		
		Indian	1869	NR				4.2			aOR: 1.20 (0.92-1.56)		
		Other Asian	1668	NR				3.1			aOR: 1.02 (0.76-1.38)		
		Other ethnicity	892	NR				1.8			aOR: 0.64 (0.38-1.06)		
Sullivan (1997)	Australia	Australia-born	7226	P0: 40.8%		0.6		7.6					
		Vietnam-born	730	P0: 40.4%		0.3		2.9					
**SUB-SAHARAN AFRICA**													
Knutzen (1977)	South Africa	Black	2302	NR		1.1	8.8	2.9				[Perinatal mortality]<br>[CH vs NT] Increased, p<0.01<br>[GH vs NT] NS<br>[PE vs NT] Increased, significance NR	
		Colored	11194	NR		1.7	10.9	2.9				[CH vs NT] Increased, significance NR<br>[GH vs NT] NS<br>[PE vs NT] Increased, p<0.001	

		Whites	1755	NR		0.6	14.1	2.9				[CH vs NT] NR<br>[GH vs NT] NS<br>[PE vs NT] Increased, p<0.001
**INTERCONTINENTAL**												
Urquia (2014)	Australia, Canada, Spain, US, Denmark and Sweden	Western Europe	184314	NR				1.8	0.1		[ref]	
		Eastern Europe	89754	NR				1.9	<0.1		PE aOR: 0.99 (0.93-1.05)<br>E aOR: 0.61 (0.41-0.90)	
		Latin America / Caribbean	1728364	NR				2.8	0.1		PE aOR: 1.63 (1.57-1.69)<br>E aOR: 1.55 (1.26-1.91)	
		South Asia	221136	NR				1.8	<0.1		PE aOR: 1.01 (0.96-1.06)<br>E aOR: 1.07 (0.83-1.38)	
		East / Southeast Asia	509088	NR				1.7	0.1		PE aOR: 0.90 (0.87-0.94)<br>E aOR: 0.80 (0.64-1.01)	
		North Africa / Middle East	200757	NR				1.4	0.1		PE aOR: 0.83 (0.79-0.87)<br>E aOR: 1.09 (0.76-1.57)	
		Sub-Saharan Africa	95986	NR				2.8	0.1		PE aOR: 1.72 (1.63-1.80)<br>E aOR: 2.12 (1.61-2.79)	

Abbreviations: AA: African American; aHR: adjusted hazards ratio; AI/AN: American Indian / Alaska Native; aOR: adjusted odds ratio; aRR: adjusted relative rate / risk; BMI: body mass index; C/SA: Central / South America(n); CC: Caucasian; CI: confidence interval; CH: chronic hypertension; E: eclampsia; ed: education; EEA: European Economic Area; F-b: foreign-born; FGR: fetal growth restriction; GH: gestational hypertension; HDP: hypertensive disorders of pregnancy; IUFD: intrauterine fetal demise; LBW: low birthweight; NH: non-Hispanic; NHB: non-Hispanic black; NHW: non-Hispanic white; NR: not reported; NS: not significant; NT: normotensive; OR: odds ratio; P0: nulliparous; P1+: primiparous or multiparous; PE: preeclampsia; POR: prevalence odds ratio; PR-b: Puero Rico-born; PTB: preterm birth; ref: reference group; resid: residence; RR: relative rate; SBP: systolic blood pressure; sec: secondary sev: severe; SGA: small for gestational age; sig: significant; sm: smoking; sPE: preeclampsia superimposed on chronic hypertension; UK: United Kingdom; US-b: US-born; US: United States of America; vs: versus. * Outcomes related to severity of disease and (potentially) HDP related adverse outcomes, including severe maternal morbidity, maternal mortality, PTB, SGA, perinatal death, neonatal morbidity and NICU admission. OR/RR/HRs are calculated in women with HDP, comparing racial and ethnic groups, unless indicated otherwise. † High risk cohort with oversampling of preterm births. ‡ Study population compared to general obstetric hospital population with same race or ethnicity.

Table S6a: Risk of hypertension after HDP in non-White populations.

		Patients		Controls		Length of follow-up *	Socio-demographic characteristics during pregnancy or at follow-up †	Hypertension				
Author (year)	Country	Type	N	Type	N			Patients	Controls	RR/OR/HR (95% CI)	Sig	Definition
**ASIA**												
Ma (2018)	China	PE	173	-	-	6 w	(Preg) BMI: HT 24.7, NT 24.6	[prev] 35.3%	-	-	-	Not defined.
Wang (2017)	China	HDP	89	No HDP	1172	2.29 y Range: 1-5 y	(Preg) BMI ≥ 28: HT 26%, NT 7%<br>Ed < 13y: HT 30%, NT 22%<br>Lowest income: HT 37%, NT 27%<br>Sm: HT 2%, NT 2%	NR	NR	aHR: 2.75 (1.63-4.65)	Sig	BP ≥ 140/90 mmHg and/or Rx.
Keepanasseril (2020)	India	PE/E	177	-	-	3 m	(FU) Age: HT 25y, NT 28y<br>P0: HT 44%, NT 70%	[prev] 18.1%	-	-	-	BP ≥ 140/90 mmHg and/or Rx.
Fatma (2017)	India	PE	50	No PE	50	NR	(FU) Age: Pt 35y, Co 36y	[prev]<br><5y: 50%<br>5-10y: 73%<br>>10y: 75%	NR	NR	NR	Not defined.
Ernawati (2019)	Indonesia	PE	42	-	-	5 y	(Preg) Age: EO 32y, LO 28y<br>P1: EO 29%, LO 44%<br>BMI: EO 25.1, LO 23.7	[prev]<br>EO PE: 76.5%<br>LO PE: 56.0%	-	-	-	BP ≥ 140/90 mmHg and/or Rx.
Watanabe (2020)	Japan	GH	17	No GDH	111	NR	(FU) Age: HT 63y, NT 58y<br>BMI: HT 24.1, NT 21.6	[prev]<br>Early onset GH: NR<br>Late onset GH: NR	NR	aOR: 3.6 (0.7-19.3)<br>aOR: 7.1 (0.8-63.7)	p=0.13<br><br>p=0.08	BP ≥ 140/90 mmHg and/or Rx.
Wagata (2020)	Japan	HDP	1585	No HDP	31827	35 y	(FU) Age: 60y<br>BMI ≥ 25: 26%	[prev] 51.4%	[prev] 36.8%	cRR: 1.4 (95% CI NR)	p<0.01	BP ≥ 140/90 mmHg and/or Rx.
Mito (2018)	Japan	GH/PE	25	No GH/PE	746	5 y	(Preg) Age: Pt 35y, Co 34y<br>BMI: Pt 20.4, Co 20.1<br>P0: Pt 60%, Co 56%<br>(FU) Sm: Pt 8%, Co 5%<br>Ed > Junior college: Pt 33%, Co 25%	[prev] All: 4.0%	[prev] 2.5%	aOR: 7.1 (2.0-25.6)	P=0.003	BP ≥ 140/90 mmHg.

							BMI: Pt 21.5, Co 20.6					
Oishi (2017)	Japan	HDP	14	No HDP	298	30.7 y	(Preg) Age: Pt 27y, Co 26y<br>(FU) Age: Pt 56y, Co 54y<br>BMI: Pt 24.6, Co 22.3<br>Sm: Pt 14%, Co 4%	[prev] 57.1%	[prev] 23.8%	cRR: 2.4 (95% CI NR)	p=0.01	BP ≥ 140/90 mmHg and/or Rx.
Watanabe (2015)	Japan	GH/PE	101	No GH/PE	1084	NR	(FU) Age: Pt 48y, Co 6y<br>BMI: Pt 21.9, Co 21.0<br>Sm: Pt 11%, Co 9%	[prev] 13.9%	[prev] 2.9%	aOR: 4.28 (2.14-8.57)	p<0.001	Use of Rx.
Kurabayashi (2013)	Japan	GH/PE	1285	No GH/PE	9171	NR	(FU) Age ≥ 45y<br>BMI ≥ 30: 2%<br>Sm: 13%	NR	NR	aOR: 2.59 (2.20-3.05)	p<0.001	Not defined.
Nohira (2013)	Japan	Sev PE	58	NT women	61	Pt: 12.3 y (3.2)<br>Co: 12.7 y (3.3)	(Preg) BMI Pt 24.7, Co 23.8<br>(FU) Pt 38y, Co 40y<br>Sm: Pt 10%, Co 10%<br>BMI: Pt 28.9, Co 25.4	[prev] 53.4%	[prev] 11.5%	cRR: 4.7 (95% CI NR)	p=0.01	Not defined.
Cho (2019-1)	Republic of Korea	PE	1910	-	-	8 y	(Preg) Age: HT 30y, NT 29y<br>Sm: HT 3%, NT 2%<br>BMI: HT 23.0, HT 20.9	[prev] 7.7%	-	-	-	BP ≥ 140/90 mmHg and/or Rx.
Cho (2019-2)	Republic of Korea	HDP‡	3391	No HDP	45674	Pt: 10.0 m (5.4)<br>Co: 9.9 m (5.3)	(Preg) Age: Pt 31y, Co 31y<br>Sm: Pt 3%, Co 3%<br>Lowest income: Pt 7%, Co 6%	[prev] 10.3%	[prev] 4.9%	cRR 2.1 (95% CI NR)	p<0.001	BP ≥ 130/85 mmHg and/or Rx.
Yang (2015)	Republic of Korea	PE	2590	No PE	47190	NR (approximately 23 y)	(FU) Age: Pt 52y, Co 53y<br>Ed ≥ high school: Pt 58%, Co 55%	[prev] 45.1%	[prev] 37.4%	aOR 1.53 (1.41-1.67)	Sig	BP ≥ 130/85 mmHg and/or Rx.
Li (2018)	Singapore<br>*55% Chinese*<br>*22% Malay*<br>*23% Indian*	GH/PE	23	No GH/PE	253	5 y	NR	NR	NR	aRR: 3.6 (1.5-8.6)	Sig	BP ≥ 140/90 mmHg and/or Rx.
Kuo (2018)	Taiwan	PE	1144	No PE/E	5180	Median: 9.8 y	(Preg) Age: Pt 30y, Co 30y	[incidence rate per 1000 person-years] 40.31	[incidence rate per 1000 person-years]	cHR: 7.31 (6.07-8.80)	p<0.001	ICD-9-CM diagnosis.

		E	151			(IQR 5.1-12.7)		42.39	4.62	cHR: 6.52 (4.49-9.47)	p<0.001	
Hwu (2016)	Taiwan	GH/PE	6347	No GH/PE	12594	Max: 12 y	(Preg) No GDM<br>Age: Pt 33y, Co 33y	[incidence rate per 1000 person-years] 8.82	[incidence rate per 1000 person-years] 0.79	aHR: 11.2 (8.19-15.2)	Sig	ICD-9-CM diagnosis.
		GH	*2727*					9.16		aHR: 11.6 (8.18-16.5)	Sig	
		PE	*3620*					8.60		aHR: 10.9 (7.83-15.1)	Sig	
Yeh (2014)	Taiwan	HDP	1260	No HDP	5040	Median: 5.8 y	(Preg) Age: Pt 30y, Co 30y	[incidence rate per 1000 person-years] 24.93	[incidence rate per 1000 person-years] 3.36	cHR: 8.29 (6.30-10.91)	Sig	ICD-9-CM diagnosis.
		GH	*725*					NR		aHR: 7.40 (4.95-111.06)	Sig	
		PE	*493*					NR		aHR: 7.65 (5.40-10.83)	Sig	
		E	*42*					NR		aHR: 6.41 (2.28-18.01)	Sig	
**EUROPE**												
Neuman (2021)	Netherlands / Black	PE	9	-	-	1 y	NR	[prev] 33.3%	-	-	-	BP ≥ 140/90 mmHg and/or Rx.
**MIDDLE AND SOUTH AMERICA**												
Facca (2018)	Brazil<br>*56% White*<br>*15% Black*<br>*28% Brown*	GH/PE	25	No GH/PE	60	Pt: 13.7 y (9.1)<br>Co: 17.3 y (11.8)	(FU) Age: Pt 44y, Co 47y<br>BMI: Pt 31.8, Co 26.5<br>Sm: Pt 24%, Co 27%	[prev] 72.0%	[prev] 36.7%	cRR: 2.0 (95% CI NR)	p=0.003	BP ≥ 140/90 mmHg and/or Rx.
Henriques (2014)	Brazil	HDP	30	No HDP	30	15.2 y (3.5)<br>Range: 10-20 y	(Preg) Age: Pt 28y, Co 25y<br>(FU) Age: Pt 43y, Co 40y<br>BMI: Pt 31.2, Co 28.5	[prev] 43.3%	[prev] 6.7%	cRR: 6.5 (95% CI NR)	p=0.001	Not defined.
Tornes (2020)	Cuba	PE	162	-	-	12 w	(Preg) Age ≥ 35y: 28%<br>BMI ≥ 30: 17%<br>P0: 48%	[prev]<br>Early onset PE: 93%<br>Late onset PE: 12%	-	-	-	BP ≥ 140/90 mmHg.
**NORTH AFRICA AND THE MIDDLE EAST**												

Amiri (2019)	Iran	PE	355	No PE	2667	Median, exposed: 9.9 y (IQR 5.5-12.8) Median, controls: 11.2 y (IQR 7.2-13.1)	(Baseline) Age: Pt 34y, Co 36y<br>BMI: Pt 28.1, Co 27.3<br>Sm: Pt 6%, Co 4%	[incidence rate per 1000 person-years] 34	[incidence rate per 1000 person-years] 22	aHR: 3.62 (2.70-4.62)	p<0.001	BP ≥ 140/90 mmHg and/or Rx.
Hashemi (2013)	Iran	GH/PE	226	No GH/PE	226	10 y	(Baseline) Age: Pt 36y, Co 36y<br>BMI: Pt 28.3, Co 28.3<br>(FU) BMI: Pt 30.0, Co 29.5	[cumulative incidence] 41.4%	[cumulative incidence] 19.4%	aRR: 2.80 (1.30-5.71)	p=0.005	BP ≥ 140/90 mmHg and/or Rx.
Shahbazian (2011)	Iran	PE	35	NT women	35	5.7 y Range: 5.2-7.3 y	(Preg) Age: Pt 26y, Co 25y<br>BMI: Pt 21.4, Co 20.9	[prev] 28.6%	v2.9%	cRR: 9.9 (1.4-74.0)	p=0.003	BP ≥ 140/90 mmHg and/or Rx.
Shammas (2000)	Jordan	GH	54	Uneventful vaginal deliveries	46	10 y	(Preg) Age: GH 32y, PE 27y, Co 31y<br>P0: GH 43%, PE 68%, Co 45%	[prev] 39%	[prev] 3%	cRR 13.0 (95% CI NR)	Sig	DBP ≥ 90 mmHg and/or Rx.
		PE	47					[prev] 23%		cRR 7.7 (95% CI NR)	Sig	
Qasim (2016)	Pakistan	HDP	66	No HDP	286	NR	(FU) Age: HT 54y, NT 37y<br>BMI: HT 31.5, NT 28.4	NR	NR	aOR: 2.2 (1.0-4.5)	[0.04)	BP ≥ 140/90 mmHg.
Fadalallah (2016)	Sudan	PE	165	-	-	6 w	(Preg) Age: HT 28y, NT 29y<br>BMI: HT 24.8, NT 24.5	[prev] 35.2%	-	-	-	Not defined.
Aykas (2015)	Turkey	PE	25	Uncomplicated pregnancies	20	Min: 5 y Pt: 6.1 y (3.6) Co: 6.1 y (4.1)	(Preg) Age Pt 27y, Co 27y<br>Sm: Pt 12%, Co 0%<br>BMI: Pt 29.1, Co 27.9<br>(FU) BMI: Pt 30.1, Co 28.6	[prev] 32%	[prev] 0%	NR	p=0.001	BP ≥ 140/90 mmHg and/or Rx.
**NORTH AMERICA**												
Lederer (2020)	US, Florida / NHB	PE	88	-	-		Age: 32y	[prev] 20.5%	-	-	-	BP ≥ 140/90 mmHg and/or Rx.

	US, Florida / Black Hispanic	PE	15	-	-	2.9 y Range 0.1-4.7 y		NR	-	-	-	
	US, Florida / White Hispanic	PE	63	-	-			[prev] 15.9%	-	-	-	
**SUB-SAHARAN AFRICA**												
Nganou-Gnindjio (2021)	Cameroon	PE/E	92	-	-	Min: 6 m	(Preg) Age ≥ 30y: Pt 83%, Co 27%<br>BMI ≥ 25: Pt 93%, Co 81%<br>Sm: Pt 0%, Co 2%	[prev] 32.6%	-	-	-	BP ≥ 140/90 mmHg.
Amougou (2019)	Cameroon	PE	140	-	-	3.7 y	(Preg) Age: HT 37y, NT 31y<br>BMI: HT 28.8, NT 25.9	[incidence rate per year] 2.85	-	-	-	BP ≥ 140/90 mmHg.
Kaze (2014)	Cameroon	Sev PE/E	54	-	-	6 m	(Preg) Age: 26y<br>BMI: 26.3<br>P0: 69%	[prev] 14.8%	-	-	-	Continuous use of Rx to maintain BP <140/90 mmHg.
		Sev PE	37					[prev] 16.2%		-	-	
		E	17					[prev] 11.8%		-	-	
Osoti (2020)	Kenya	GH/PE	63	No GH/PE	131	6 m	(Preg) Age: Pt 33y, Co 33y<br>Ed ≥ secondary: Pt 65%, Co 73%<br>BMI: Pt 31.9, Co 30.7<br>(FU) BMI: Pt 32.8, Co 31.9	[prev] 23.8%	[prev] 9.1%	cRR: 2.6 (95% CI NR)	p=0.006	BP ≥ 140/90 mmHg.
Ishaku (2021-1)	Nigeria	*GH*	*69*	-		1 y	(Preg) Age: GH 33y, PE 28y, E 25y<br>BMI: GH 31.6, PE 27.7, E 24.9<br>P0: GH 12%, PE 23%, E 40%	[prev] 22%	-	-	-	BP ≥ 140/90 mmHg.
		PE/E	*167*					[prev] 61%		-	-	
Olagbuji (2012)	Nigeria	GH/PE	198	-	-	6 w	(Preg) Age ≥ 35y: HT 31%, NT 15%<br>P0: HT 41%, NT 43%<br>Sm: HT 0%, NT 0%	[prev] 25.8%	-	-	-	Not defined.

Ntlemo (2021)	South Africa<br>*87% Black African*<br>*8% Coloured*<br>*2% White*<br>*2% Indian*	PE	150	NT women	150	6 w	(Preg) Age: Pt 26y, Co 23y	[prev] 32.7%	[prev] 22.7%	cRR: 1.4 (95% CI NR)	NR	BP ≥ 130/85 mmHg and/or Rx.
Mooij (2021)	Tanzania	Sev PE/E	24	NT women	72	5-7 y	(FU) Age (median): Pt 26, Co 32<br>Ed, none: Pt 25%, Co 8%	[prev] 29%	[prev] 13%	cRR 2.2 (95% CI NR)	NR	Not defined.
		Sev PE	*9*					[prev] 44%		cRR 3.4 (95% CI NR)	NR	
Nakimuli (2013)	Uganda	PE	188	-	-	3 m	(FU) Age: HT 27y, NT 24y<br>P0: HT 27%, NT 58%	[prev] 34%	-	-	-	BP ≥ 140/90 mmHg and/or Rx.
Ndayambagye (2010)	Uganda	PE/E	195	-	-	6 w	(Preg) Age ≥ 35y: HT 15%, HT 4%<br>Ed, none: HT 1%, NT 3%<br>Sm: HT 2%, NT 3%	[prev] 27.7%				Not defined.

Abbreviations: aHR: adjusted hazard ratio; aOR; adjusted odds ratio; aRR: adjusted risk ratio; BMI: body mass index; BP: blood pressure; cHR: crude hazard ratio; Co: control group; cRR: crude risk ratio; DBP: diastolic blood pressure; E: eclampsia; Ed: education level; FU: follow-up; GDM: gestational diabetes mellitus; GH: gestational hypertension; HDP: hypertensive disorders of pregnancy; HT: hypertensive; ICD: International Classification of Diseases; IQR: interquartile range; m: months; NHB: non-Hispanic Black; NR: not reported; NS: not significant; NT: normotensive; P0: nulliparous; PE: preeclampsia; Preg: pregnancy; prev: prevalence; Pt: patient group; Rx: medical treatment; SD: standard deviation; sev: severe; sig: significant; Sm: smoking; w: weeks; y: years. * Mean length of follow (SD) unless otherwise indicated, in patients and controls. † Socio-demographic characteristics during pregnancy or at follow-up among patients and controls, or among hypertensive or normotensive patients at follow-up; mean or percentages unless otherwise indicated. ‡ Identified by original authors as PE, redefined as HDP based on ICD-10-CM codes mentioned in the article.

Table S6b: Risk of type 2 diabetes mellitus and prediabetes after HDP in non-White populations.

		Patients		Controls				Type 2 diabetes mellitus				
Author (year)	Country	Type	N	Type	N	Length of follow-up *	Socio demographic characteristics †	Patients	Controls	RR/OR/HR (95% CI)	Sig	Definition
**ASIA**												
Fatma (2017)	India	PE	50	No PE	50	NR	(FU) Age: Pt 35y, Co 36y	[prev] <5y: 25%<br>5-10y:33%<br>>10y: 50%	NR	NR	NR	Not defined.
Ernawati (2019)	Indonesia	PE	42	-	-	5 y	(Preg) Age: EO 32y, LO 28y<br>P1: EO 29%, LO 44%<br>BMI: EO 25.1, LO 23.7	[prev]<br>Early onset PE: 23.5%<br>Late onset PE: 16.0%	NR	NR	NR	FPG ≥ 7.0 mmol/L.
Mito (2018)	Japan	GH/PE	25	No GH/PE	746	5 y	(Preg) Age: Pt 35y, Co 34y<br>BMI: Pt 20.4, Co 20.1<br>P0: Pt 60%, Co 56%<br>(FU) Sm: Pt 8%, Co 5%<br>Ed > Junior college: Pt 33%, Co 25%<br>BMI: Pt 21.5, Co 20.6	[prev] 0%	[prev] 0.8%	NR	NR	FPG ≥ 7.0 mmol/L, HbA1c ≥ 6.5%, or Rx.
Oishi (2017)	Japan	HDP	14	No HDP	298	30.7 y	(Preg) Age: Pt 27y, Co 26y<br>(FU) Age: Pt 56y, Co 54y<br>BMI: Pt 24.6, Co 22.3<br>Sm: Pt 14%, Co 4%	[prev] 0.0%	[prev] 1.7%	NR	p=0.79	FPG > 7.0 mmol/L and HbA1c > 6.5%, or Rx.
Watanabe (2015)	Japan	GH/PE	101	No GH/PE	1084	NR	(FU) Age: Pt 48y, Co 6y<br>BMI: Pt 21.9, Co 21.0<br>Sm: Pt 11%, Co 9%	[prev] 1.0%	[prev] 1.1%	aOR: 0.57 (0.07-4.62)	p=0.6	Use of Rx.
Kurabayashi (2013)	Japan	GH/PE	1285	No GH/PE	9171	NR	(FU) Age ≥ 45y<br>BMI ≥ 30: 2%<br>Sm: 13%	[prev] NR	[prev] NR	aOR: 1.34 (0.95-1.88)	p=0.09	Not defined.
Cho (2019-2)	Republic of Korea	HDP‡	3391	No HDP	45674	Pt: 10.0 m (5.4)<br>Co: 9.9 m (5.3)	(Preg) Age: Pt 31y, Co 31y<br>Sm: Pt 3%, Co 3%<br>Lowest income: Pt 7%, Co 6%	[prev] 11.5%	[prev] 9.5%	cRR: 1.2	p<0.001	FPG ≥ 5.6mmol/L

Yang (2015)	Republic of Korea	PE	2590	No PE	47190	NR (approximately mean 23 y)	(FU) Age: Pt 52y, Co 53y<br>Ed ≥ high school: Pt 58%, Co 55%	[prev] 22.5%	[prev] 21.3%	aHR: 1.13 (1.03-1.25)	Sig	FPG ≥ 5.6mmol/L or Rx.
Kuo (2018)	Taiwan	*PE*	*1144*	No PE/E	5180	Median: 9.8 y (IQR 5.1-12.7)	(Preg) Age: Pt 30y, Co 30y	[incidence rate per 1000 persons-years] 11.27	[incidence rate per 1000 persons-years] 2.45	cHR: 5.42 (4.01-7.32)	p<0.001	ICD-9-CM diagnosis.
		E	*151*					7.30		cHR: 3.84 (1.99-7.40)	p<0.001	
Hwu (2016)	Taiwan	GH/PE	6347	No GH/PE	12594	Max: 12 y	(Preg) No GDM<br>Age: Pt 33y, Co 33y	[incidence rate per 1000 persons-years] 8.06	incidence rate per 1000 persons-years] 2.55	aHR: 3.15 (2.55-3.89)	Sig	ICD-9-CM diagnosis.
		GH	*2727*					8.52		aHR: 3.31 (2.52-4.35)	Sig	
		PE/E	*3620*					7.78		aHR: 3.05 (2.40-3.88)	Sig	
Yeh (2014)	Taiwan	HDP	1260	No HDP	5040	Median: 5.8 y	(Preg) Age: Pt 30y, Co 30y	[prev] 4.29%	[prev] 1.41%	cRR: 3.0	p<0.001	ICD-9-CM diagnosis.
Wang (2012)	Taiwan	GH/PE	1139	No GH/PE	4527	Pt: 8.1 y (2.1)<br>Co: 8.2 y (2.0)	(Preg) Age: 29y	[incidence rate per 1000 persons-years] 4.24	[incidence rate per 1000 persons-years] 0.83	aHR: 3.42 (2.07-5.64)	p<0.001	ICD-9-CM diagnosis.
		GH	*488*					2.07		aHR: 1.73 (0.78-3.81)	NS	
		PE	*651*					5.81		aHR: 4.15 (2.48-6.95)	p<0.001	
Wang (2011)	Taiwan	GH/PE	1092	No GH/PE	4715	Pt: 6.6 y (1.6)<br>Co: 6.4 (1.6)	(Preg) Age: Pt 30y, Co 28y	[prev] 2.8%	[prev] 0.8%	aOR: 2.74 (1.67-4.50)	p<0.001	ICD-9-CM diagnosis.
Soonthornpun (2009)	Thai	Sev PE	13	Uncompl	26	Pt: 2.6 y (1.6)<br>Co: 4.5 y (2.8)	(FU) Age: Pt 31y, Co 32y<br>BMI: Pt 25.8, Co 24.6	[prev] 46.2%	[prev] 11.5%	cRR: 4.0	p=0.04	IGT: 2-hPC 7.77-11.1 mmol/L.
**MIDDLE AND SOUTH AMERICA**												
Henriques (2014)	Brazil	HDP	30	No HDP	30	15.2 y (3.5)	(Preg) Age: Pt 28y, Co 25y<br>(FU) Age: Pt 43y, Co 40y	[prev] 23.3%	[prev] 3.3%	cRR 7.06 (95%CI NR)	p=0.02	Not defined.

							BMI: Pt 31.2, Co 28.5					
**NORTH AFRICA AND THE MIDDLE EAST**												
Hashemi (2013)	Iran	GH/PE	226	No GH/PE	226	10 y	(Baseline) Age: Pt 36y, Co 36y<br>BMI: Pt 28.3, Co 28.3<br>(FU) BMI: Pt 30.0, Co 29.5	[cumulative incidence] 37.2%	[cumulative incidence] 12.0%	aRR: 2.37 (1.03-5.47)	p=0.04	FPG ≥ 7 mmol/L, 2-hPG ≥ 11.1 mmol/L or Rx.
Aykas (2015)	Turkey	PE	25	Uncompl	20	6.1 y (3.6-4.1)	(Preg) Age Pt 27y, Co 27y<br>Sm: Pt 12%, Co 0%<br>BMI: Pt 29.1, Co 27.9<br>(FU) BMI: Pt 30.1, Co 28.6	[prev] 0%	[prev] 0%	NR	NR	Not defined.
**SUB-SAHARAN AFRICA**												
Osoti (2020)	Kenya	GH/PE	63	No GH/PE	131	6 m	(Preg) Age: Pt 33y, Co 33y<br>Ed ≥ secondary: Pt 65%, Co 73%<br>BMI: Pt 31.9, Co 30.7<br>(FU) BMI: Pt 32.8, Co 31.9	[prev] 7.9%	[prev] 1.6%	aRR: 6.20 (1.07-35.76)	p=0.03	FPG ≥ 5.6mmol/L or Rx.

Abbreviations: aHR: adjusted hazard ratio; aOR; adjusted odds ratio; aRR: adjusted risk ratio; BMI: body mass index; cHR: crude hazard ratio; Co: control group; cRR: crude risk ratio; E: eclampsia; Ed: education level; FPG: fasting plasma glucose; FU: follow-up; HbA1c: hemoglobin A1c; GH: gestational hypertension; HDP: hypertensive disorders of pregnancy; ICD: International Classification of Diseases; IGT: impaired glucose tolerance; IQR: interquartile range; m: months; NR: not reported; NS: not significant; P0: nulliparous; PE: preeclampsia; Preg: pregnancy; prev: prevalence; Pt: patient group; Rx: medical treatment; SD: standard deviation; sev: severe; sig: significant; Sm: smoking; uncompl: uncomplicated pregnancies; y: years. * Mean length of follow (SD) unless otherwise indicated, in patients and controls. † Socio-demographic characteristics during pregnancy or at follow-up among patients and controls; mean or percentages unless otherwise indicated. ‡ Identified by original authors as PE, redefined as HDP based on ICD-10-CM codes mentioned in the article.

Table S6c: Risk of dyslipidemia after HDP in non-White populations.

		Patients		Controls				Dyslipidemia				
Author (year)	Country	Type	N	Type	N	Length of follow-up *	Socio demographic characteristics †	Patients	Controls	RR/OR/HR (95%CI)	Sig	Definition
**ASIA**												
Fatma (2017)	India	PE	50	No PE	50	NR	(FU) Age: Pt 35y, Co 36y	[prev] <5y: 20%<br>5-10y: 33%<br>>10y: 50%	NR	NR	NR	Not defined.
Ernawati (2019)	Indonesia	PE	42	-	-	5 y	(Preg) Age: EO 32y, LO 28y<br>P1: EO 29%, LO 44%<br>BMI: EO 25.1, LO 23.7	[prev] High TG:<br>- Early onset PE: 58.8%<br>- Late onset PE: 40.0%	NR	NR	NR	TG ≥ 1.7 mmol/L.
								[prev] Low HDL-C:<br>- Early onset PE: 52.9%<br>- Late onset PE: 52.0%				HDL-C < 1.3 mmol/L.
Mito (2018)	Japan	GH/PE	25	No GH/PE	746	5 y	(Preg) Age: Pt 35y, Co 34y<br>BMI: Pt 20.4, Co 20.1<br>P0: Pt 60%, Co 56%<br>(FU) Sm: Pt 8%, Co 5%<br>Ed > Junior college: Pt 33%, Co 25%<br>BMI: Pt 21.5, Co 20.6	[prev] 12.0%	[prev] 14.2%	NR	NS	HDL-C ≤ 1.0 mmol/L, TG ≥ 1.7 mmol/L, LDL-C ≥ 3.6 mmol/L or Rx.
Oishi (2017)	Japan	HDP	14	No HDP	298	30.7 y	(Preg) Age: Pt 27y, Co 26y<br>(FU) Age: Pt 56y, Co 54y<br>BMI: Pt 24.6, Co 22.3<br>Sm: Pt 14%, Co 4%	[prev] 21.4%	[prev] 11.4%	cRR: 1.9 (95% CI NR)	p=0.23	HDL-C < 1.0 mmol/L, TG > 1.7 mmol/L, LDL-C > 3.6 mmol/L or Rx.
Watanabe (2015)	Japan	GH/PE	101	No GH/PE	1084	NR	(FU) Age: Pt 48y, Co 46y<br>BMI: Pt 21.9, Co 21.0<br>Sm: Pt 11%, Co 9%	[prev] 9.9%	[prev] 2.6%	aOR: 3.20 (1.42-7.22)	p=0.005	Use of Rx.
Kurabayashi (2013)	Japan	GH/PE	1285	No GH/PE	9171	NR	(FU) Age ≥ 45y<br>BMI ≥ 30: 2%<br>Sm: 13%	High TC: NR	NR	aOR: 1.42 (1.22-1.66)	p<0.001	Not defined.

Cho (2019-2)	Republic of Korea	HDP‡	3391	No HDP	45674	Pt: 10.0 m (5.4)<br>Co: 9.9 m (5.3)	(Preg) Age: Pt 31y, Co 31y<br>Sm: Pt 3%, Co 3%<br>Lowest income: Pt 7%, Co 6%	[prev] High TG: 12.9%	[prev] 9.7%	cRR 1.33 (95% CI NR)	p<0.001	TG ≥ 1.7 mmol/L.
								[prev] Low HDL-C: 25.5%	[prev] 21.1%	cRR 1.21 (95% CI NR)	p<0.001	HDL-C < 1.3 mmol/L.
Yang (2015)	Republic of Korea	PE	2590	No PE	47190	NR (approximately mean 23 y)	(FU) Age: Pt 52y, Co 53y<br>Ed ≥ high school: Pt 58%, Co 55%	[prev] High TG: 23.2%	[prev] 22.5%	aOR: 1.09 (0.99-1.20	NS	TG ≥ 1.7 mmol/L.
								[prev] Low HDL-C: 35.2%	[prev] 34.6%	aOR: 1.06 (1.97-1.15)	NS	HDL-C < 1.3 mmol/L.
Kuo (2018)	Taiwan	PE	1144	No PE/E	5180	Median: 9.8 y (IQR 5.1-12.7)	(Preg) Age: Pt 30y, Co 30y	[incidence rate per 1000 persons-years] 15.05	[incidence rate per 1000 persons-years] 4.44	cHR 3.40 (2.73-4.24)	p<0.001	ICD-9-CM diagnosis.
		E	151					[incidence rate per 1000 persons-years] 12.18		cHR: 2.75 (1.68-4.51)	p<0.001	
Yeh (2014)	Taiwan	HDP	1260	No HDP	5040	Median: 5.8 y	(Preg) Age: Pt 30y, Co 30y	[prev] 4.48%	[prev] 2.78%	cRR 1.61 (95% CI NR)	p<0.001	ICD-9-CM diagnosis.
Wang (2011)	Taiwan	GH/PE	1092	No GH/PE	4715	Pt: 6.6 y (1.6)<br>Mean, controls: 6.4 (1.6)	(Preg) Age: Pt 30y, Co 28y	[prev] 1.5%	[prev] 0.5%	aOR 2.29 (1.20-4.35)	p<0.05	ICD-9-CM diagnosis.
**MIDDLE AND SOUTH AMERICA**												
Henriques (2014)	Brazil	HDP	30	No HDP	30	15.2 y (3.5)<br>Range: 10-20 y	(Preg) Age: Pt 28y, Co 25y<br>(FU) Age: Pt 43y, Co 40y<br>BMI: Pt 31.2, Co 28.5	[prev] 20.0%	[prev] 6.7%	cRR 2.99 (95% CI NR)	p=0.12	Not defined.
**NORTH AFRICA AND THE MIDDLE EAST**												
Hashemi (2013)	Iran	GH/PE	226	No GH/PE	226	10 y	(Baseline) Age: Pt 36y, Co 36y<br>BMI: Pt 28.3, Co 28.3<br>(FU) BMI: Pt 30.0, Co 29.5	[cumulative incidence] 87.2%	[cumulative incidence] 66.3%	aRR 2.43 (1.24-4.70)	p=0.01	TC ≥ 6.2 mmol/L, LDH-C ≥ 4.1 mmol/L, TG ≥ 2.3 mmol/L or HDL-C < 0.9 mmol/L.

**SUB-SAHARAN AFRICA**												
Osoti (2020)	Kenya	GH/PE	63	No GH/PE	131	6 m	(Preg) Age: Pt 33y, Co 33y<br>Ed ≥ secondary: Pt 65%, Co 73%<br>BMI: Pt 31.9, Co 30.7<br>(FU) BMI: Pt 32.8, Co 31.9	[prev] High TG: 15.9%	[prev] 5.5%	aRR: 3.25 (1.16-9.10)	p=0.01	TG ≥ 1.7 mmol/L.

Abbreviations: aOR; adjusted odds ratio; aRR: adjusted risk ratio; BMI: body mass index; cHR: crude hazard ratio; Co: control group; cRR: crude risk ratio; E: eclampsia; Ed: education level; FU: follow-up; GH: gestational hypertension; HbA1c: hemoglobin A1c; HDL-C: high-density lipoprotein cholesterol; HDP: hypertensive disorders of pregnancy; ICD: International Classification of Diseases; IQR: interquartile range; LDL-C: low-density lipoprotein cholesterol; m: months; NR: not reported; NS: not significant; P0: nulliparous; PE: preeclampsia; Preg: pregnancy; prev: prevalence; Pt: patient group; Rx: medical treatment; SD: standard deviation; sig: significant; Sm: smoking; TC: total cholesterol; TG: triglycerides; y: years. * Mean length of follow (SD) unless otherwise indicated, in patients and controls. † Socio-demographic characteristics during pregnancy or at follow-up among patients and controls; mean or percentages unless otherwise indicated. ‡ Identified by original authors as PE, redefined as HDP based on ICD-10-CM codes mentioned in the article.

Table S6d: Risk of chronic kidney disease after HDP in non-White populations.

Author (year)	Country	Patients<br>Type	Patients<br>N	Controls<br>Type	Controls<br>N	Length of follow-up *	Socio demographic characteristics †	Chronic kidney disease<br>Patients	Chronic kidney disease<br>Controls	RR/OR/HR (95% CI)	Sig	Definition
**ASIA**												
Mito (2018)	Japan	GH/PE	25	No GH/PE	746	5 y	(Preg) Age: Pt 35y, Co 34y<br>BMI: Pt 20.4, Co 20.1<br>P0: Pt 60%, Co 56%<br>(FU) Sm: Pt 8%, Co 5%<br>Ed > Junior college: Pt 33%, Co 25%<br>BMI: Pt 21.5, Co 20.6	[prev] 0%	[prev] 0.7%	NR	NS	Not reported.
Oishi (2017)	Japan	HDP	14	No HDP	298	30.7 y	(Preg) Age: Pt 27y, Co 26y<br>(FU) Age: Pt 56y, Co 54y<br>BMI: Pt 24.6, Co 22.3<br>Sm: Pt 14%, Co 4%	[prev] 21.4%	[prev] 4.0%	aOR: 4.85 (1.04-22.6)	p=0.03	EGFR < 60 mL/min/1.73 m^2.
Wu (2021)	Taiwan	GH/PE	29852	No GH/PE	119408	5.7 y	(Preg) Age: Pt 31y, Co 31y	[incidence rate per 10.000 persons-years] 42.7	[incidence rate per 10.000 persons-years] 8.2	aHR: 4.26 (3.80-4.78)	Sig	ICD-9-CM diagnosis.
Hwu (2016)	Taiwan	GH/PE	6347	No GH/PE	12594	Max: 12 y	(Preg) No GDM<br>Age: Pt 33y, Co 33y	[prev] 0.39%	[prev] 0.17%	cRR 2.3 (95% CI NR)	p=0.09	ICD-9-CM diagnosis.
Wu (2014)	Taiwan	HDP	13633	No HDP	930841	Median: 9 y (IQR 7.1-10.0)	(Preg) Age: Pt 30y, Co 28y	[incidence rate per 10.000 persons-years] 38.8	[incidence rate per 10.000 persons-years] 2.6	aHR: 10.64 (7.53-15.05)	p<0.001	ICD-9-CM diagnosis.
		GH	*2361*					19.7		aHR: 5.82 (2.15-15.77)	Sig	
		CH	*731*					63.3		aHR: 15.99 (5.89-43.38)	Sig	
		PE/E	*8609*					33.5		aHR: 9.46 (6.10-14.68)	Sig	
		sPE	*594*					172.0		aHR: 44.72 (22.59-88.51)	Sig	

Wang (2013)	Taiwan	GH/PE	26651	No GH/PE	213397	6.3 y	(Preg) Age: Pt 30y, Co 30y	[incidence rate per 10.000 persons-years] CKD: 7.24	[incidence rate per 10.000 persons-years] CKD: 0.67 ESRD: 0.34	aHR: 9.38 (7.09-12.4)	Sig	ICD-9-CM diagnosis.
								ESRD: 4.72		aHR: 12.4 (8.54-18.0)	Sig	
		GH	8653					ESRD: 3.40		aHR: 9.03 (5.20-15.7)	Sig	
		PE/E	17998					ESRD: 5.33		aHR: 14.0 (9.43-20.7)	Sig	
**NORTH AFRICA AND THE MIDDLE EAST**												
Behboudi-Gandevani (2020)	Iran	PE	177	No PE	1674	Median, exposed: 7.8 y (IQR 5.2-10.4) Median, controls: 7.3 y (IQR 4.7-11.0)	(Baseline) Age: Pt 31y, Co 34y BMI: Pt 27.5, Co 26.5 Sm: Pt 6%, Co 4%	[cumulative incidence per 100.000 women at median follow-up time] 35	[cumulative incidence per 100.000 women at median follow-up time] 36	aHR 0.88 (0.63-1.23)	p=0.45	EGFR < 60 mL/min/1.73 m^2.
Shahbazian (2011)	Iran	PE	35	NT women	35	5.7 y	(Preg) Age: Pt 26y, Co 25y BMI: Pt 21.4, Co 20.9	[prev] 20.0%	[prev] 0%	NR	p=0.01	ACR ≥ 30mg/mmol.
**SUB-SAHARAN AFRICA**												
Kaze (2014)	Cameroon	Sev PE/E	54	-	-	6 m	(Preg) Age: 26y BMI: 26.3 P0: 69%	[prev] 1.8%	-	-	-	Proteinuria > 200 mg/24h.
		Sev PE	37					2.7%		-	-	
		E	17					0%		-	-	
Ishaku (2021-3)	Nigeria	HDP	278	-	59	1 y	(Preg) Age: GH 35y, PE 28y, E 25y BMI: GH 31.6, PE 27.7, E 24.9 P0: GH 12%, PE 23%, E 40%	[prev] 3.5%	5.5%	NR	NR	EGFR < 60 mL/min/1.73 m^2.
		GH	*69*					3.6%		NR	NR	
		CH	*42*					8.3%		NR	NR	
		PE	*136*					3.8%		NR	NR	
		E	*31*					0.0%		NR	NR	

Abbreviations: ACR: albumin creatinine ratio; aHR: adjusted hazard ratio; aOR; adjusted odds ratio; BMI: body mass index; Co: control group; CH: chronic hypertension; CKD: chronic kidney disease; E: eclampsia; Ed: education level; eGFR: estimated glomerular filtration rate; ESRD: end-stage renal disease; FU: follow-up; GH: gestational hypertension; HDP: hypertensive disorders of pregnancy; ICD: International Classification of Diseases; IQR: interquartile range; m: months; NR: not reported; NS: not significant; NT: normotensive; P0: nulliparous; PE: preeclampsia; Preg: pregnancy; prev: prevalence; Pt: patient group; SD: standard deviation; sev: severe; sig: significant; Sm: smoking; sPE: preeclampsia superimposed on chronic hypertension; y: years: * Mean length of follow (SD) unless otherwise indicated, in patients and controls. † Socio-demographic characteristics during pregnancy or at follow-up among patients and controls; mean or percentages unless otherwise indicated.

Table S6e: Risk of metabolic syndrome after HDP in non-White populations.

Author (year)	Country	Patients: Type	Patients: N	Controls: Type	Controls: N	Length of follow-up *	Socio demographic characteristics †	Metabolic syndrome: Patients	Metabolic syndrome: Controls	RR/OR/HR (95% CI)	Sig	Definition
**ASIA**												
Lu (2011)	China	Sev PE	62	-	-	1-2 y: 40%<br>2-3 y: 32%<br>>3 y: 27%	NR	[prev] 37%	NR	NR	NR	NCEP-ATP III criteria.§
								[prev] 39%				IDF standards.¶
Fatma (2017)	India	PE	50	No PE	50	NR	(FU) Age: Pt 35y, Co 36y	[prev] <5y: 50%<br>5-10y: 66%<br>>10y: 75%	NR	NR	NR	Not defined.
Ernawati (2019)	Indonesia	PE	42	-	-	5 y	(Preg) Age: EO 32y, LO 28y<br>P1: EO 29%, LO 44%<br>BMI: EO 25.1, LO 23.7	[prev]<br>Early onset PE: 58.8%<br>Late onset PE: 44.0%	NR	NR	NR	≥3 of the following criteria: 1) WC ≥ 88 cm, 2) fasting TG ≥ 1.7 mmol/L, 3) fasting HDL-C < 1.3 mmol/L or Rx, 4) BP ≥ 140/90 or Rx, or 5) FPG > 7.0 mmol/L or Rx.
Mito (2018)	Japan	GH/PE	25	No GH/PE	746	5 y	(Preg) Age: Pt 35y, Co 34y<br>BMI: Pt 20.4, Co 20.1<br>P0: Pt 60%, Co 56%<br>(FU) Sm: Pt 8%, Co 5%<br>Ed > Junior college: Pt 33%, Co 25%<br>BMI: Pt 21.5, Co 20.6	[prev] 0.0%	[prev] 0.5%	NR	NS	WC ≥ 80 cm plus ≥2 of the following: 1) TG > 1.7 mmol/L, 2) BP ≥ 130/85 mmHg, or 3) FPG > 6.1 mmol/L.
Cho (2019-2)	Republic of Korea	HDP‡	3391	No HDP	45674	Pt: 10.0 m (5.4)<br>Co: 9.9 m (5.3)	(Preg) Age: Pt 31y, Co 31y<br>Sm: Pt 3%, Co 3%<br>Lowest income: Pt 7%, Co 6%	[prev] 4.9%	[prev] 2.7%	aOR: 1.34 (1.11-1.61)	p<0.001	≥3 of the following criteria: 1) WC ≥ 85 cm, 2) fasting TG ≥ 1.7 mmol/L, 3) fasting HDL-C < 1.3 mmol/L or Rx, 4) BP ≥ 130/85 or Rx, or 5) FPG > 5.6 mmol/L or Rx.
Yang (2015)	Republic of Korea	PE	2590	No PE	47190	NR (approximate	(FU) Age: Pt 52y, Co 53y	[prev] 27.3%	[prev] 25.4%	aOR 1.23 (1.12-1.35)	Sig	≥3 of the following criteria: 1) WC ≥ 80 cm, 2) fasting TG ≥

						ly mean 23 y)	Ed ≥ high school: Pt 58%, Co 55%					1.7 mmol/L, 3) fasting HDL-C < 1.3 mmol/L or Rx, 4) BP ≥ 130/85 or Rx, or 5) FPG > 5.6 mmol/L or Rx.
Li (2018)	Singapore *55% Chinese* *22% Malay* *23% Indian*	GH/PE	23	No GH/PE	253	5 y	NR	[prev] NR	[prev] NR	aRR: 2.8 (0.8-9.9)	NS	≥3 of the following criteria: 1) WC ≥ 88 cm, 2) fasting TG ≥ 1.7 mmol/L, 3) fasting HDL-C < 1.3 mmol/L, 4) BP ≥ 130/85 or Rx, or 5) FPG ≥ 7.0 mmol/L or 2hPG ≥ 11.0mmol/L or Rx.
**MIDDLE AND SOUTH AMERICA**												
Facca (2018)	Brazil *56% White* *15% Black* *28% Brown*	GH/PE	25	No GH/PE	60	Pt: 13.7 y (9.1) Co: 17.3 y (11.8)	(FU) Age: Pt 44y, Co 47y BMI: Pt 31.8, Co 26.5 Sm: Pt 24%, Co 27%	[prev] 64.0%	[prev] 21.7%	cRR: 2.95 (95% CI NR)	p<0.001	NCEP ATP III criteria. §
**SUB-SAHARAN AFRICA**												
Osoti (2020)	Kenya	GH/PE	63	No GH/PE	131	6 m	(Preg) Age: Pt 33y, Co 33y Ed ≥ secondary: Pt 65%, Co 73% BMI: Pt 31.9, Co 30.7 (FU) BMI: Pt 32.8, Co 31.9	[prev] 34.9%	[prev] 11.5%	aRR 3.01 (1.58-5.71)	p=0.001	≥3 of the following criteria: 1) WC ≥ 88 cm, 2) fasting TG ≥ 1.7 mmol/L, 3) fasting HDL-C < 1.3 mmol/L or Rx, 4) BP ≥ 130/85 or Rx, or 5) FPG > 5.6 mmol/L or Rx.
Ishaku (2021-2)	Nigeria	HDP	278	No HDP	59	1 y	(Preg) Age: GH 33y, PE 28y, E 25y BMI: GH 31.6, PE 27.7, E 24.9 P0: GH 12%, PE 23%, E 40%	[incidence rate per 1000 person-years] 57.5	[incidence rate per 1000 person-years] 16.9	NR	NR	BMI > 30 with ≥2 of the following criteria: 1) TG ≥ 1.7mmol/L, 2) HDL < 1.29mmol/L, 3) BP ≥ 130/85 or 4) FPG ≥ 5.6 mmol/L.
		GH	*69*					71.0				
		CH	*42*					0.0				
		PE	*136*					60.0				
		E	*31*					29.0				
Ntlemo (2021)	South Africa	PE	150	NT women	150	6 w	(Preg) Age: Pt 26y, Co 23y	32.0%	22.0%	cRR: 1.45 (95% CI NR)	p=0.05	≥3 of the following criteria: 1) WC ≥ 80 cm, 2) fasting TG >

87% Black African<br>*8% Coloured*<br>*2% White*<br>*2% Indian*	1.7 mmol/L, 3) fasting HDL-C < 1.3 mmol/L, 4) BP ≥ 130/85 or Rx, or 5) FPG > 5.6 mmol/L.

Abbreviations: aOR; adjusted odds ratio; aRR: adjusted risk ratio; BMI: body mass index; BP: blood pressure; Co: control group; cRR: crude risk ratio; E: eclampsia; Ed: education level; FPG: fasting plasma glucose; FU: follow-up; GH: gestational hypertension; HDL-C: high-density lipoprotein cholesterol; HDP: hypertensive disorders of pregnancy; ICD: International Classification of Diseases; IQR: interquartile range; LDL-C: low-density lipoprotein cholesterol; m: months; NR: not reported; NS: not significant; NT: normotensive; P0: nulliparous; PE: preeclampsia; Preg: pregnancy; prev: prevalence; Pt: patient group; Rx: medical treatment; SD: standard deviation; sev: severe; sig: significant; Sm: smoking; TG: triglycerides; w: weeks; WC: waist circumference; y: years. * Mean length of follow (SD) unless otherwise indicated, in patients and controls. † Socio-demographic characteristics during pregnancy or at follow-up among patients and controls; mean or percentages unless otherwise indicated. ‡ Identified by original authors as PE, redefined as HDP based on ICD-10-CM codes mentioned in the article. § NCEP ATP III criteria: ≥3 of the following criteria: 1) waist circumference ≥ 88 cm, 2) fasting TG ≥ 1.7 mmol/L, 3) fasting HDL-C < 1.3 mmol/L, 4) BP ≥130/85 or 5) FPG ≥5.6mmol/L. ¶ IDF standards: WC ≥ 80 cm plus ≥2 of the following criteria: 1) TG ≥ 1.70 mmol/L or Rx, 2) HDL-C < 1.29 mmol/L or previously diagnosed or Rx, 3) BP ≥ 130/85 or Rx, or FPG ≥ 5.6 mmol/L or previously diagnosed T2DM.

Table S6f: Risk of cardiovascular disease after HDP in non-White populations.

Author (year)	Country	Patients Type	Patients N	Controls Type	Controls N	Length of follow-up *	Socio demographic characteristics †	Cardiovascular disease (combined) Patients	Controls	RR/OR/HR (95% CI)	Sig	Definition
**ASIA**												
Wu (2021)	Taiwan	GH/PE	29852	No GH/PE	119408	5.7 y	(<u>Preg</u>) Age: Pt 31y, Co 31y	[incidence rate per 1000 person-years] MACE: 1.36 Vascular event: 0.88 CVD mortality: 0.81	[incidence rate per 1000 person-years] MACE: 0.83 Vascular event: 0.51 CVD mortality: 0.37	aHR: 2.15 (1.89-2.45) aHR: 2.31 (1.97-2.72) aHR: 2.02 (1.63-2.49)	Sig Sig Sig	ICD-9-CM diagnosis. MACE: CVA, CAD or death. Vascular event: CVA or CAD.
Kuo (2018)	Taiwan	PE	1144	No PE/E	5180	Median: 9.8 y (IQR 5.1-12.7)	(<u>Preg</u>) Age: Pt 30y, Co 30y	[incidence rate per 1000 person-years] CHF: 1.12	[incidence rate per 1000 person-years] CHF: 0.15	cHR 7.39 (2.86-19.06)	p<0.001	ICD-9-CM diagnosis.
		E	151					CHF: 1.40		cHR: 9.07 (1.88-43.68)	p=0.006	
Yeh (2014)	Taiwan	<u>HDP</u>	<u>1260</u>	No HDP	5040	Median: 5.8 y	(<u>Preg</u>) Age: Pt 30y, Co 30y	[incidence rate per 1000 person-years] CVD: 9.74	[incidence rate per 1000 person-years] 3.99	aHR: 2.45 (1.81-3.32)	Sig	ICD-9-CM diagnosis.
		GH	*725*					NR		cHR: 2.00 (1.26-3.18)	Sig	
		PE	*493*					NR		cHR: 3.02 (2.00-4.56)	Sig	
		E	*42*					NR		cHR: 1.38 (0.28-6.83)	NS	
Lin (2011)	Taiwan	(s)PE/E	NR	No PE/E	NR	0-5 y	(<u>Preg</u>) P0: 46% Age 25-34y: 68%	[incidence rate per 1000 person-years] MACE: 1.60 MACE-related mortality: 0.17	NR	aHR: 12.6 (2.4-66.3) aHR: 6.4 (3.8-10.9)	p=0.003 p<0.001	ICD-9-CM diagnosis. MACE: MI, HF, PCI, CABG, CVA, malignant dysrhythmia, cardiac shock, thrombolysis or implantable

												cardioverter defibrillator.
**MIDDLE AND SOUTH AMERICA**												
Da Silva (2014)	Brazil	PE	65	NT women	65	5 y	(FU) Age: Pt 31y, Co 31y<br>BMI: Pt 30.7, Co 26.7	[prev] 26.15%	[prev] 6.15%	cRR 4.25 (95% CI NR)	p=0.002	Not defined.
**NORTH AMERICA**												
Malek (2020-1)	US, South Carolina / NHB	GH/PE	25924	No GH, PE or CH	102676	1-5 y	All races/ethnicities:<br>(Preg) Age: Pt 30y, Co 27y<br>Ed < high school: Pt 13%, Co 18%<br>Lowest income: Pt 35%, Co 26%<br>Sm: Pt 11%, Co 12%<br>BMI: Pt 33.4, Co 26.5	[incidence rate per 1000 person-years]<br>HF: 2.28	[incidence rate per 1000 person-years]<br>HF: 0.48	aHR: 3.74 (3.12-4.49)	Sig	HF event of death defined by ICD-9-CM diagnosis.
		CH	715					HF: 0.84		aHR: 1.11 (0.36-3.49)	NS	
		sPE	4905					HF: 4.30		aHR: 4.88 (3.78-6.29)	Sig	
Cirillo (2015)	US, California / African American	GH	NR	No GH	NR	66 y	NR	CVD mortality: NR	CVD mortality: NR	aHR: 1.8 (1.09-2.82)		ICD-7, -8, 9 and 10 diagnosis.
**SUB-SAHARAN AFRICA**												
Taa Nguimbis Esseme (2019)	Cameroon	HDP	104	No HDP	385	Min: 6 m	(FU) Sm: 3%<br>Ed < secondary: 17%	NR	NR	aOR: 0.83 (0.51-1.34)	NS	ICD-10 and drug prescriptions. CVD: IHD, CVA and hypertensive disease.
		GH	*45*					NR		aOR: 2.33 (0.99-5.50)	NS	
		PE	*55*					NR		aOR: 0.28 (0.10-0.72)	Sig	

Abbreviations: aHR: adjusted hazard ratio; aOR; adjusted odds ratio; BMI: body mass index; CABG: coronary artery bypass grafting; CAD: coronary artery disease; CHD: congestive heart failure; cHR: crude hazard ratio; Co: control group; cRR: crude relative risk; CVA: cerebrovascular accident; CVD: cardiovascular disease; E: eclampsia; Ed: education level; FU: follow-up; GH: gestational hypertension; HDP: hypertensive disorders of pregnancy; HF: heart failure; HR: hazard rate; ICD: International Classification of Diseases; IHD: ischemic heart disease; IQR: interquartile range; m: months; MACE: major adverse cardiovascular event; MI: myocardial infarction; NHB: non-Hispanic Black; NR: not reported; NS: not significant; NT: normotensive; P0: nulliparous; PCI: percutaneous coronary intervention; PE: preeclampsia; Preg: pregnancy; prev: prevalence; Pt: patient group; SD: standard deviation; sev: severe; sig: significant; Sm: smoking; sPE: preeclampsia superimposed on chronic hypertension; y: years. * Mean length of follow (SD) unless otherwise indicated, in patients and controls. † Socio-demographic characteristics during pregnancy or at follow-up among patients and controls; mean or percentages unless otherwise indicated.

Table S6g: Risk of cerebrovascular accidents (stroke) after HDP in non-White populations.

		Patients		Controls				Cerebrovascular accidents (stroke)				
Author (year)	Country	Type	N	Type	N	Length of follow-up *	Socio demographic characteristics †	Patients	Controls	RR/OR/HR (95% CI)	Sig	Definition
**ASIA**												
Park (2018)	Republic of Korea	PE	25698	No PE		1 y	(Preg) Age: CVD 31y, no CVD 31y<br>P0: CVD 47%, no CVD 53%	[incidence per 1000 persons] 4.73	[incidence per 1000 persons] 2.77	aOR: 1.64 (1.37-1.98)	Sig	ICD-10 diagnosis.
Hung (2022)	Taiwan	HDP	13617	No HDP 54468		1-17 y	(Preg) Age ≥ 35y: Pt 24%, Co 24%<br>Lowest income: Pt 9%, Co 9%	[prev] 1.53%	[prev] 1.15%	aHR: 1.71 (1.46-2.00)	p<0.001	Not defined.
		GH	*NR*					NR		aHR: 1.68 (1.13-2.52)	p<0.01	
		CH	*NR*					NR		aHR: 1.27 (0.97-1.68)	NS	
		PE/E	*NR*					NR		aHR: 2.00 (1.63-2.45)	p<0.001	
		sPE	*NR*					NR		aHR: 3.86 (1.91-7.82)	p<0.001	
Huang (2020)	Taiwan	HDP	41870	No HDP	125610	13 y	(FU) Age: Pt 35y, Co 37y	[incidence rate per 1000 person-years] 1.21	[incidence rate per 1000 person-years] 1.06	aHR: 2.1 (1.8-2.5)	p<0.001	ICD-9-CM diagnosis.
		GH	*NR*					0.93		NR	NS	
		CH	*NR*					1.94		aHR: 3.4 (2.9-4.0)	p<0.001	
		Mild / unsp PE	*NR*					1.04		NR	NS	
		Sev PE	*NR*					1.21		aHR 2.1 (1.8-2.5)	p<0.001	
		E	*NR*					2.60		aHR 4.6 (3.9-5.4)	p<0.001	

		sPE/E	*NR*					1.77		aHR: 3.1 (2.7-3.7)	p<0.001	
		Unsp HDP	*NR*					1.52		aHR 2.6 (2.3-3.1)	p<0.001	
Kuo (2018)	Taiwan	PE	1144	No PE/E	5180	Median: 9.8 y (IQR 5.1-12.7)	(Preg) Age: Pt 30y, Co 30y	[incidence rate per 1000 person-years]<br>All stroke: 0.92<br>Hemorrhagic stroke: 0.10<br>Infarction: 0.41	[incidence rate per 1000 person-years]<br>All stroke: 0.26<br>Hemorrhagic stroke: 0.11<br>Infarction: 0.09	cHR: 3.47 (1.46-8.23)<br>cHR: 0.93 (0.11-7.97)<br>cHR: 4.76 (1.19-19.03)	p=0.005<br>p=0.95<br>p=0.03	ICD-9-CM diagnosis.
		E	151					All stroke: 2.81<br>Hemorrhagic stroke: 2.11<br>Infarction: 0.70		cHR: 10.71 (3.45-33.24)<br>cHR: 19.74 (4.71-82.68)<br>cHR: 7.84 (0.88-70.14)	p<0.001<br>p<0.001<br>p=0.06	
Hwu (2016)	Taiwan	GH/PE	6347	No GH/PE	12594	Max: 12 y	(Preg) No GDM<br>Age: Pt 33y, Co 33y	[prev] 0.18%	[prev] 0.12%	cRR: 1.5 (95% CI NR)	p=0.31	ICD-9-CM diagnosis.
Lin (2011)	Taiwan	(s)PE/E	NR	No PE/E	NR	0-5 y	(Preg) P0: 46%<br>Age 25-34y: 68%	[incidence rate per 1000 person-years]<br>1.11	[incidence rate per 1000 person-years]<br>NR	aHR: 14.5 (1.3-165.1)	p=0.03	ICD-9-CM diagnosis.
Wang (2011)	Taiwan	GH/PE	1092	No GH/PE	4715	Pt: 6.6 y (1.6)<br>Co: 6.4 (1.6)	(Preg) Age: Pt 30y, Co 28y	[incidence rate per 1000 person-years]<br>3.01	[incidence rate per 1000 person-years]<br>1.28	aHR: 2.04	p<0.01	ICD-9-CM diagnosis.
**NORTH AMERICA**												
Malek (2021-2)	US, South Carolina / NHB	GH/PE	26581	No GH, PE or CH	105092	1-5 y	All races/ethnicities:<br>(Preg) Age: Pt 31y, Co 28y<br>Ed < high school: Pt 14%, Co 18%<br>Lowest income: Pt 36%, Co 27%<br>Sm: Pt 14%, Co 16%<br>BMI: Pt 33.7, Co 26.7	[incidence rate per 1000 person-years]<br>0.32	[incidence rate per 1000 person-years]<br>0.17	aHR: 1.67 (1.14-2.46)	Sig	Fatal and non-fatal CHD and stroke. ICD-9/10-CM diagnosis.
		CH	761					0.53		aHR: 2.35 (0.57-9.65)	NS	
		sPE	5350					0.97		aHR: 4.00 (2.48-6.45)	Sig	
	US, South Carolina / Hispanic	GH/PE	4087	No GH, PE or CH	36612			[incidence rate per 1000 person-years]<br>0.15	[incidence rate per 1000 person-years]<br>0.04	aHR: 2.15 (0.43-10.80)	NS	
		CH	114					NR	NR	NR	NR	

	sPE	342		NR	NR	NR	NR	

Abbreviations: aHR: adjusted hazard ratio; aOR; adjusted odds ratio; BMI: body mass index; cHR: crude hazard ratio; Co: control group; CVD: cardiovascular disease; E: eclampsia; Ed: education level; FU: follow-up; GH: gestational hypertension; HDP: hypertensive disorders of pregnancy; ICD: International Classification of Diseases; IQR: interquartile range; NHB: non-Hispanic Black; NR: not reported; NS: not significant; P0: nulliparous; PE: preeclampsia; Preg: pregnancy; prev: prevalence; Pt: patient group; SD: standard deviation; sev: severe; sig: significant; Sm: smoking; sPE: preeclampsia superimposed on chronic hypertension; unsp: unspecified; y: years. * Mean length of follow (SD) unless otherwise indicated, in patients and controls. † Socio-demographic characteristics during pregnancy or at follow-up among patients and controls, or among patients with and without CVD at follow-up; mean or percentages unless otherwise indicated.

Table S6h: Risk of ischemic heart disease after HDP in non-White populations.

		Patients		Controls				Ischemic heart disease				
Author (year)	Country	Type	N	Type	N	Length of follow-up *	Socio demographic characteristics †	Patients	Controls	RR/OR/HR (95% CI)	Sig	Definition
**ASIA**												
Hwu (2016)	Taiwan	GH/PE	6347	No GH/PE	12594	Max: 12 y	(Preg) No GDM<br>Age: Pt 33y, Co 33y	[prev] 0.08%	[prev] 0.13%	cRR: 0.6 (95% CI NR)	p=0.44	ICD-9-CM diagnosis.
Lin (2011)	Taiwan	(s)PE/E	NR	No PE/E	NR	0-5 y	(Preg) P0: 46%<br>Age 25-34y: 68%	NR	NR	aHR: 13.0 (4.6-6.3)	p<0.001	ICD-9-CM diagnosis.
Wang (2011)	Taiwan	GH/PE	1092	No GH/PE	4715	Pt: 6.6 y (1.6)<br>Co: 6.4 (1.6)	(Preg) Age: Pt 30y, Co 28y	[prev] 2.2%	[prev] 2.0%	aOR: 0.96 (0.60-1.53)	NS	ICD-9-CM diagnosis.
**NORTH AFRICA AND THE MIDDLE EAST**												
Borna (2012)	Iran	Mild PE	49	No PE	641	NR	(FU) Age: CVD 58y, no CVD 56y<br>BMI: CVD 29.4, no CVD 29.3	NR	NR	aHR: 16.92 (95% CI NR)	p<0.001	CAD: > 50% stenosis in one of the three main coronary arteries.
**NORTH AMERICA**												
Malek (2021-2)	US, South Carolina / NHB	GH/PE	26581	No GH, PE or CH	105092	1-5 y	All races/ethnicities:<br>(Preg) Age: Pt 31y, Co 28y<br>Ed < high school: Pt 14%, Co 18%<br>Lowest income: Pt 36%, Co 27%<br>Sm: Pt 14%, Co 16%<br>BMI: Pt 33.7, Co 26.7	[incidence rate per 1000 person-years] 1.52	[incidence rate per 1000 person-years] 0.59	aHR: 2.33 (1.94-2.80)	Sig	ICD-9/10-CM diagnosis.
		CH	761					1.58		aHR: 1.95 (0.86-5.39)	NS	
		sPE	5350					3.51		aHR: 4.02 (3.13-5.18)	Sig	
	US, South Carolina / Hispanic	GH/PE	4087	No GH, PE or CH	36612			0.34	0.11	aHR: 2.68 (1.06-6.81)	Sig	
		CH	114					1.77		aHR: 16.0 (2.10-122)	Sig	
		sPE	342					1.18		aHR: 9.38 (2.10-41.8)	Sig	

Wichmann (2019)	US, South Carolina / Black	PE	137	No PE / GDM / PTB	445	Pt: 21.9 y<br>Co: 23.0 y	(Preg) Age (median): Pt 22y, Co 21y<br>(FU): Age ≥ 50: Pt 23%, Co 23%<br>Sm: Pt 40%, Co 37%	NR	NR	CAD, aOR: 1.47 (0.90-2.38)<br>Ob CAD, aOR: 1.34 (0.61-2.99)	p=0.12<br>p=0.46	CAD: ≥ 20% luminal narrowing.<br>Obstructive CAD: ≥50% luminal narrowing.

Abbreviations: aHR: adjusted hazard ratio; aOR; adjusted odds ratio; BMI: body mass index; CAD: coronary artery disease; Co: control group; CVD: cardiovascular disease; E: eclampsia; Ed: education level; FU: follow-up; GDM: gestational diabetes mellitus; GH: gestational hypertension; HDP: hypertensive disorders of pregnancy; ICD: International Classification of Diseases; IQR: interquartile range; NHB: non-Hispanic Black; NR: not reported; NS: not significant; P0: nulliparous; PE: preeclampsia; preg: pregnancy; prev: prevalence; Pt: patient group; PTB: preterm birth; SD: standard deviation; sev: severe; sig: significant; Sm: smoking; sPE: preeclampsia superimposed on chronic hypertension; y: years. * Mean length of follow (SD) unless otherwise indicated, in patients and controls. † Socio-demographic characteristics during pregnancy or at follow-up among patients and controls, or among patients with and without CVD at follow-up; mean or percentages unless otherwise indicated.
